# Quantitative Particle Uptake by Cells as Analyzed by Different Methods

**DOI:** 10.1002/anie.201906303

**Published:** 2019-12-13

**Authors:** Sumaira Ashraf, Alaa Hassan Said, Raimo Hartmann, Marcus‐Alexander Assmann, Neus Feliu, Peter Lenz, Wolfgang J. Parak

**Affiliations:** ^1^ Fachbereich Physik Philipps Universität Marburg 35037 Marburg Germany; ^2^ Institute of Industrial Biotechnology Government College University Lahore Punjab 54000 Pakistan; ^3^ Electronics and Nano Devices lab (END) Department of Physics Faculty of Sciences South Valley University 83523 Qena Egypt; ^4^ Fraunhofer Institute for High-Speed Dynamics Ernst Mach Institute 79104 Freiburg Germany; ^5^ Fachbereich Physik und Chemie, CHyN Universität Hamburg 20146 Hamburg Germany; ^6^ Institute of Nano Biomedicine and Engineering Key Laboratory for Thin Film and Microfabrication Technology of the Ministry of Education Department of Instrument Science and Engineering School of Electronic Information and Electrical Engineering Shanghai Jiao Tong University Shanghai China

**Keywords:** bioanalytics, cells, nanoparticles, particle uptake

## Abstract

There is a large number of two‐dimensional static in vitro studies about the uptake of colloidal nano‐ and microparticles, which has been published in the last decade. In this Minireview, different methods used for such studies are summarized and critically discussed. Supplementary experimental data allow for a direct comparison of the different techniques. Emphasis is given on how quantitative parameters can be extracted from studies in which different experimental techniques have been used, with the goal of allowing better comparison.

## Graphical abstract

**Time for a recount**: In vitro studies on the uptake of colloidal nano‐ and microparticles by cells are summarized and critically discussed. Supplementary experimental data allow for a direct comparison of the different techniques. Emphasis is given on how quantitative parameters can be extracted from studies in which different experimental techniques have been used.

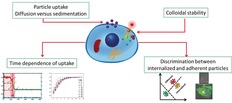

## 1 A Basic Introduction to Particle Uptake by Cells

Almost any publication dealing with nano‐ and microparticles as delivery vehicles in the context of potential nanomedicines, in the following referred to as particles, includes an uptake study of the particles in static two‐dimensional (2D) in vitro cell culture.^1^ While the relevance of such studies for later in vivo applications is matter to debate, the fact is that there are thousands of such studies published. However, as there are no commonly accepted protocols, many of those uptake studies are carried out in different ways, are rather qualitative than quantitative, which makes it hard to compare different studies. In fact, the experimental details may strongly influence results.1c Thus in this Minireview, different methods will be described and reviewed, with an emphasis on the extraction of quantitative parameters. This approach then allows for a direct comparison of experimental data as obtained from different methods. Methods will be explained by a set of data which has been recorded for this article and which is described in detail in the Supporting Information.

Uptake of particles by cells involves several different steps. First, the particle has to reach the cell and make contact. This process is driven by an interplay of particle diffusion and sedimentation.^2^ Note that sedimentation may be less relevant in vivo, but in this Minireview conditions refer to the conditions used in most studies, which is two‐dimensional static adherent in vitro cell cultures. This limitation is because not all the methods described herein could be applied in the same way to other exposure scenarios. In case cells are non‐adherent single particle tracking studies, which will be explained later, could not be applied in the same way. This is due to problems with maintaining one cell or one particle in the focus of a microscope, which is required for tracking particle entry into cells. Also in cases in which flow conditions are applied, particle tracking methods for quantifying uptake would have to be adopted. This is also due to complications of tracking particles in a flowing medium. Three‐dimensional cultures are not homogeneous entities, as for example, cells on the surface of cell spheroids^3^ are directly in contact with the medium containing the particles, whereas cells in the middle of the spheroid are not. Quantification methods based on determining the average amount of particles per cell, such as with elemental analysis, would lead to an average number of internalized particles per cell. Owing to the inhomogeneity of the spheroids, this would neglect the real situation in which cells at the surface of the spheroid would have internalized more particles than cells inside the spheroid. Also for in vivo scenarios most of the methods described herein could not be applied.

Having touched cells, in two‐dimensional static adherent in vitro cell cultures, particles may stick or migrate along the outer cell membrane. Frequently this is not mediated by defined receptor–ligand interaction, for example by ligands on the particle surface binding specifically to membrane proteins, but rather by less‐specific interactions, such as electrostatic attraction.^4^ After a certain dwelling time, particles may be endocytosed. After intracellular cascades the particles then typically reside in endosomes/lysosomes.^5^ Once a cell divides the particles are passed to both daughter cells.^6^ Particles may be also released from endosomes/lysosomes to the extracellular medium by exocytosis.^7^ Exocytosis of particles is however strongly size‐dependent,^8^ and thus is not always an efficient pathway for particle clearance from cells.6c Apart from particle uptake by endocytosis there are thus several pathways leading to the reduction of the number of intracellular particles.

## 2 Important Parameters Required to Describe Experimental Methods

To describe particle uptake by cells, most importantly all the details about the experimental set‐up need to be provided.1c, 10 Concerning the particles, this involves details about the metrics with which particle concentrations are quantified.1a This can be in the form of molar particle concentrations, mass concentrations, molar concentrations of elements, total particle volume, total particle surface, total particle absorption, total particle fluorescence, etc.^11^ In most of the cases these different metrics cannot be unequivocally converted between each other, and conversion is only possible based on certain approximations and assumptions.^12^ In fact, correlation of particle uptake to certain physicochemical parameters of the particles may strongly depend on the used metrics.^13^ Note, the conditions for a used metric may change during the actual particle exposure. To give a few examples, if particles degrade (e.g. ion release from metal particles), then the number of metal atoms per particle will diminish over time.^14^ In case fluorescence is used as quantifier, fluorophores may be pH‐dependent and thus fluorescence can be quenched/enhanced upon particle incorporation in acidic endosomes/lysosomes.^15^ Thus, it is desirable to use metrics in which the read‐out quantifying particle uptake remains unchanged along the incorporation trajectory of the particles by cells. Dose‐dependent toxic effects of the particles need to be considered.^16^ Thus, accompanying viability studies should verify that at the used exposure conditions there is no acute particle‐induced toxicity,^17^ as this obviously would massively interfere with particle uptake. Furthermore, in particular the colloidal stability of the particles should be experimentally investigated under the used exposure conditions. Particle agglomeration leads to larger effective particle sizes, causing the particles to sediment on top of cells and thus artificially enhanced uptake by cells.2a, 18 Last but not least, batch‐to‐batch variations in particle synthesis should be taken into account. In many uptake studies one batch of particles is used, and error analysis often only involves measuring several times the number of incorporated particles per cell of one and the same experiment. This error thus only corresponds to the experimental error in the used read‐out technology. Ideally, if the amount of available sample permits, exposure should be carried out with different cells on different days, also involving different particle batches. The error from such measurements will give the “real” base variation. Changes in cellular uptake upon different particle properties should be always put into context of this base variation. In case the effect is at the same order of magnitude as the base variation, this effect rather will not be significant to the varied particle properties.

Concerning cell culture, this involves providing information about i) the cell density (i.e. the number of seeded cells per surface area of the culture substrate), ii) the height of the cell culture medium above cells (i.e. when the surface area of the culture substrate is known, the added volume of medium), iii) the used cell medium, and iv) optionally also some information about the used cell line, such as proliferation rate (i.e. the reciprocal time required until subsequent cell division has led to the double number of cells),8b cell surface area and cell volume.1c, 8b This is needed, as i) particle internalization depends on cell density, that is, cells in a confluent cell layer incorporate particles differently than isolated cells. ii) While it might seem intuitive to think that having the double particle concentration in half of the cell culture results to the same exposure conditions in terms of particles added per cell, this in fact in general is not true.1c Higher particle concentration in a smaller volume allows particles to reach the adherent cells on the bottom of the culture substrate faster than in the case of lower particle concentrations in a larger volume, though the number of particles added per cell is the same.1c, 2a iii) In general there is less particle uptake under serum supplemented than under serum depleted cell culture conditions.^19^ iv) As the cells divide, intracellular particle concentrations get diluted and thus the incubation time of cells with particles need to be put into the context of the reciprocal proliferation rate.6b, 8b, 20 Cells with larger surface area and/or volume may easier come into contact with particles,8b and thus absolute particle uptake in terms of internalized particles per cell also needs to consider these parameters. Only if all these conditions are provided, can the results be quantitatively compared to the results of other studies.

## 3 Potential Errors which Complicate Quantitative Analysis

Concerning read‐out, it is important to point out that most methodologies do not distinguish between internalized particles and particles adherent only to the outer cell membrane, which are wrongfully counted as internalized particles. The time a particle resides merely attached to the cell membrane before actual endocytosis takes place can strongly vary between different cell lines^21^ and particles.^22^ Some particle geometries, for example, tend to remain stuck to the cell membrane without getting internalized.^23^ If the problem to be addressed requires uptake of particles corrected for adherent particles, there are several approaches to either remove adherent particles before measurements, or to exclude them from the uptake statistics. First, extensive washing can be performed, optionally involving also digestive enzymes, such as trypsin. However, such washing procedures may impair cells. Upon strong washing, for example, cells can be detached from the cell culture substrate. Second, the particle‐quantifying parameters required for measurements may be disabled. In case particles are quantified by elemental concentrations (e.g. via inductively coupled plasma mass spectrometry, ICP‐MS), adherent particles may be dissolved by chemical etching.^24^ In the case of fluorescent particles, a quencher may be added, which causes loss of fluorescence of the adherent particles.^25^ For colloidal quantum dots (QDs), for example, metal ions are efficient quenchers.^26^ In such cases, the cell membrane acts as barrier, so that etching/quenching only occurs for extracellular, but not for intracellular particles. Third, the intracellular location of the particles may be determined by actively labelling of the probes. This can be done by colocalization of particles with immunostained endosomes/lysosomes,^13^ or by using pH‐sensitive fluorophores associated with the particles,^27^ which allows particle location to be verified in endosomes/lysosomes by the local acidic pH.27c, 28 Also active sensing elements can be used.^29^

## 4 Useful Parameters for the Quantification of Particle Uptake

**Figure 1:**
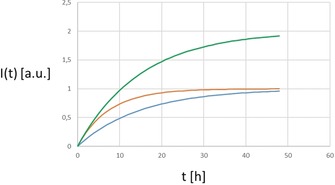
Time‐dependent particle intensity *I*(*t*) versus the time to which cells have been exposed to particles given as function *I*(*t*)=*I*_0_ (1−exp(−*t*/*T*_1/2_), with the two parameters *I*_0_ and *T*_1/2_ (red curve: *I*_0_=1, *T*_1/2_=7.5 h; blue curve: *I*_0_=1, *T*_1/2_=15 h; green curve: *I*_0_=2, *T*_1/2_=7.5 h). The kinetics of particle uptake (in terms of 1/*T*_1/2_) are twice as fast for the red and green curves compared to the blue curve. Whereas, the total amount of internalized particles under saturation conditions (in terms of *I*_0_) is double for the green as compared to the red and blue curves. To determine the curves, at least two time points are required.

Having outlined some general aspects which should be considered, now the “quantification” aspect will be discussed. In many uptake studies, results are provided in qualitative terms, such as statements like certain types of cells “better/faster/more” incorporate certain particles. However, looking at Figure 1 explains the severe limits of such statements. In Figure 1 the “intensity” *I* of particles found per cell is plotted versus the incubation time *t*. This “intensity” may be experimentally determined in different ways. In case of fluorescence‐labelled particles *I* could refer to the fluorescence intensity per cell. In the case of elemental analysis, it would refer to the amount of a certain element per cell. In general, a saturation based curve, such as shown in Figure 1, will be expected (note that there are more sophisticated analytical models available^30^). Particle concentration per cell may not rise continuously. The details may vary and a precise analysis would exceed the objective of this Minireview, but the time scales of different effects, such as diffusion‐limited supply of particles and particle translocation from the membrane into intracellular vesicles, and particle dilution/loss due to proliferation and exocytosis have to be considered.1e A cell might be, for example, “saturated” with particles at its surface and in this case doubling the extracellular particle concentration would not result in the double amount of internalized particles. Thus, quantitative uptake studies should involve at least two different particle concentrations, which verify being in the linear uptake regime, that is, doubling the particle concentration results in twice the number of incorporated particles. Different studies in the linear dose regime can be compared with each other. There should also a series of different incubation times, whereby incubation times should be extended until saturation is reached. From such a time‐dependent study, two different parameters can be extracted, see Figure 1. First, a time parameter, such as the time *T*_1/2_ which is required until a cell has taken up 1−1/*e*≈63 % of the particles as compared to saturation conditions. Second, a parameter quantifying the amount of particles, such as the particle intensity *I*_0_ under saturation conditions. How the time and quantification parameters look in detail will depend on the actual methodology used, but it is important to point out that both parameters are necessary. This leads to the statement that one concentration and one or two time points are in general not enough for quantifying particle uptake in a meaningful way other than stating that particles are internalized by cells.

Time‐dependent uptake curves in the way pointed out in Figure 1 can be obtained with different approaches.^13^, ^31^ There are, however, different considerations to be taken into account. First, for the first quantifier, the *I*‐axis in Figure 1, that is, the question “how much”, either the cells or the particles may be the reference point. When referring to cells, this could involve the percentage of cells which have internalized particles, which is, for example, frequently used in flow cytometry studies,^21^, ^32^ or the percentage of cells which have a certain number of particles incorporated, which is often used in case the number of discrete particles in a cell can be directly counted.^13^, 19a, 33 When referring to particles, this could be the percentage of particles which have been internalized, which, for example, is done when the amount of particles remaining in the cell medium and the amount of particles inside cells are quantified with elemental analysis.1a, 2a, 24a, 24c Second, also the *t*‐axis in Figure 1, the second quantifier, addressing the question “how fast”, needs to be defined. In this case the time point zero is the flexible variable. In the easiest case, *t*=0 refers to the time when the cells have been exposed to the particles and thus, *t* would refer to the incubation time. In this case however, the time is a convolution of the time particles need to reach cells and the time for actual endocytosis. In single particle experiments *t*=0 can be defined as the point of time at which a particle first touches a cell, or when endocytosis starts.^34^ In fact, changes in the definition of the time axis may lead to different response curves.^35^ Thus, it is paramount to precisely state the used metrics, to warrant comparability between different studies. In the following different techniques will be compared.

Note that in this Minireview, micrometer‐sized polyelectrolyte capsules fabricated by layer‐by‐layer assembly^36^ are used as model particle systems for the experimental part of this work. While the considerations made in this Minireview in general apply to particles ranging from nanometer to micrometer dimensions, for the purpose of better visualization, micrometer‐sized particles are used herein as the experimental model. In Figure captions the particles are thus referred to as the experimentally used capsules.

## 5 Particle Uptake as Analyzed by Single‐Particle Tracking

**Figure 2:**
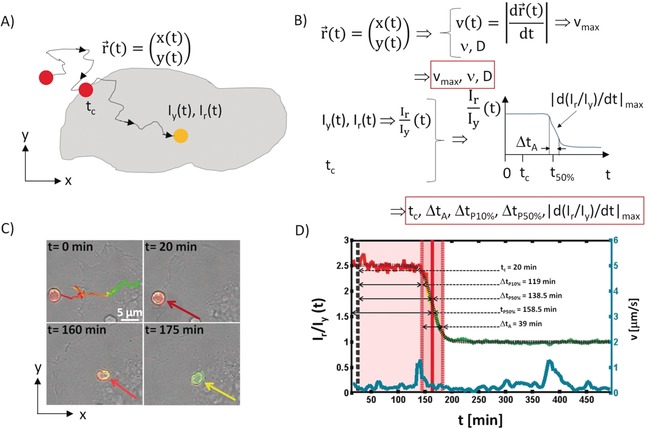
A) Sketch of the trajectory (*x*(*t*), *y*(*t*)) of a fluorescent particle, for which the fluorescence changes depending on the local pH, as measured by a ratiometric approach based on the fluorescence *I*_y_(*t*) and *I*_r_(*t*) recorded at two different emission wavelengths. The particle dose must be low enough to allow for the tracking of individual particles. B) Plot of the time‐dependent ratiometric read‐out *I*_r_/*I*_y_(*t*) originating from the traced particle over time, and the parameters which can be extracted from such traces. C) Example of 4 images from a time‐lapse series, in which HeLa cells had been incubated with capsules with integrated pH‐sensitive fluorophores as model particles, showing an overlay of phase contrast and the two (*I*_y_, *I*_r_) fluorescence channels in false colors (yellow, green). These data were obtained for positively charged capsules in the presence of serum for *N*_caps/cell_(added)=5 capsules added per cell. For details see Table 1 and the Supporting Information. D) *I*_r_/*I*_y_(*t*) data obtained from the time‐lapse series shown in (C) for the indicated capsule. The following data were extracted for this particular trajectory: *t*_c_=20 min, Δ*t*_A_=39 min, Δ*t*_P50 %_=139 min, Δ*t*_P10 %_=119 min, and |d(*I*_r_/*I*_y_)/dt)|_max_=−0.107 min^−1^.

The first methodology to be discussed is single‐particle tracking. For this approach, particles need to be able to provide sufficient contrast, so that individual particles can be detected.^37^ Note, that for single‐particle tracking in principle it is not mandatory that single particles can be resolved. In the case of particles being smaller than the lateral resolution of the detection system, it is possible to work with very dilute particle concentrations, which statistically make it extremely unlikely that two particles are so close to each other that they cannot be resolved as two individual particles and thus would be wrongly considered as just one particle.^34^, ^38^ Optical techniques conveniently allow for single‐particle tracking, such as recording the fluorescence^39^ or the scattering signal^40^ of individual particles. For quantifying uptake of particles, cells are exposed to the particles at a dilute particle concentration *N*_caps/cell_(added), referring to the number of particles (in the form of capsules in the experimental part) added per cell. The position of the particles (*x*(*t*), *y*(*t*)) is measured from the time‐lapse images, see Figure 2 B. The particles’ trajectories in general will be in three dimensions.^41^ However, often images are recorded within one focal plane, typically lying parallel to the cell culture substrate (*x*−*y*‐plane) at a height *z*, forming a cross section of the cells. Alternatively the focus can be adjusted over time, putting for each image the focal plane at the height *z* from the particles above the culture substrate. From these two‐dimensional (*x*(*t*),*y*(*t*)) trajectories, the following parameters can be directly extracted, see Figure 2 B: the time‐dependent velocity of particle movement *v*(*t*)=[(d*x*(*t*)/d*t*)^2^+(d*y*(*t*)/d*t*)^2^]^1/2^, and from this the maximum velocity *v*_max_,^34^ the fractal dimension *D* of the trajectory,^42^ and the exponent *ν* which describes how the end‐to‐end distance of the particle trajectory scales with the contour length of the trajectory.^43^ For the precise definitions of *D* and *ν* we refer to the Supporting Information. In addition, from these images the time *t*_c_ can be determined, at which, after addition of particles to cells, the observed particle first touches a cell. As the particle may dwell on the outer cell membrane for a significant time, an additional experimental parameter for verifying whether the particle is outside or inside the cell needs to be employed. This can be done by live cell staining of endosome/lysomes, which is possible via genetic transfection.^34^ Alternatively, in the case of fluorescence images obtained with pH‐sensitive fluorophores linked to the particles,27b, 44 the color of the fluorophore may indicate the location of the particle.27c, 45 The commercially available fluorophore SNARF (seminaphtharhodafluor‐1) for example, has emission in the yellow and red under acidic and neutral/alkaline conditions, respectively. Particles outside cells in the neutral/slightly alkaline cell medium thus fluoresce red (with intensity *I*_r_(*t*)), whereas upon endocytosis the local environment around the particles is acidified, and thus the color of the emission shifts to yellow (with intensity *I*_y_(*t*)). Thus, by measuring two colors, this ratiometric approach allows the change in local pH around the particle to be determined. In this way the onset of endocytosis can be determined from the time‐dependent *I*_r_/*I*_y_ data.27c Note that endocytosis is a gradual process, in which the pH subsequently decreases. This can be seen for example in Figure 2 D, which shows the acidification of the local environment around one capsule, as a model particle, upon endocytosis. During endocytosis the particle will be first engulfed by cellular membrane, before it is located in the forming intracellular membrane vesicle. Lowering of pH inside this vesicle may be delayed in regard to the actual initiation of endocytosis, that is, the start of membrane wrapping around the particle. The time‐dependent *I*_r_/*I*_y_ data thus allow the beginning of acidification to be determined, which will be used as start of endocytosis, knowing that there may be a short time gap involved. From the *I*_r_/*I*_y_(*t*) curve several parameters can be extracted, such as the maximum change in ratiometric read‐out |d(*I*_r_/*I*_y_)/dt)|_max_ and the time interval Δ*t*_A_ this change takes, and the time *t*_50 %_ when the ratiometric read‐out has changed by 50 % after the start of incubation. Also the time intervals Δ*t*_P10 %_ and Δ*t*_P50 %_ can be derived, which indicate when the ratiometric readout has dropped by 10 and 50 %, respectively, after the time point *t*_c_ of first contact with the cell. The different parameters are visualized in Figure 2 B. In Figure 2 C four images out of 179 images of a time‐laps recording are shown, in which cells have been incubated with capsules loaded with SNARF. In the first image the trajectory (together with the time‐dependent color of one capsule) of one capsule is shown and the change from red to yellow fluorescence can clearly be seen (note that false colors have been used for the overlay of the red and yellow fluorescence channels). In Figure 2 D the corresponding ratiometric read‐out *I*_r_/*I*_y_(*t*) over time is plotted and the extracted parameters are given. For each such experiment, one set of parameters is obtained. To get significant statistics, at least 20 trajectories should be investigated per data point, which makes this method time‐consuming.

The question now arises which of the extracted parameters contain relevant information. In Table 1 the parameters for experiments involving incubating HeLa cells with capsules are summarized. Refer to the Supporting Information for the experimental details. Two parameters have been varied, the sign of the charge of the capsules (+ or −), and the incubation conditions, that is, with (w) or without (w/o) the presence of serum. The number of added capsule *N*_caps/cell_(added) was kept low to conveniently allow the tracing of individual capsules. As analysis is performed on the level of individual capsules, and the capsules are expected not to interact with each other, the incubation dose of capsules should not influence the uptake time of one individual capsule. If more capsules are added, the time that one of the added capsules touches a cell is reduced, as there are more capsules present. The average time for a capsule to make contact with a cell, that is, *t*_c_, however would remain the same. Looking into the data of Table 1 allows analysis of the dependence of particle (i.e. capsule) uptake on two parameters, namely the sign of charge and presence of serum. Positively charged capsules (“+”) take longer to stick to cells than negatively (“−”) charged ones, either under serum supplemented (“w”) or serum‐free (“w/o”) culture conditions, with more than double the *t*_c_ values. It thus takes longer for the positively charged capsules to stick to the cell membrane than for the negative charged ones. The objective of this Minireview is not to analyze the reasons for this rather unexpected finding. As cells have a global net negative charge, it would be rather assumed that the positively charged capsules should have stuck faster to the cells. However, it must be taken into account that in this particular case the capsules are rather big, that is, micrometer size, which may result in different behavior as compared to smaller particles, and that this result specifically may depend on the particle/cell combination used. In the case of serum‐containing versus serum‐free culture, in case of the positively charged capsules *t*_c_ is bigger in the presence of serum, and for the negatively charged capsules *t*_c_ is not relevantly affected by the presence of serum. In case of the acidification time Δ*t*_A_ and the processing time Δ*t*_P10 %_, there is a very strong effect of the presence of serum. For positively as well as for the negatively charged capsules, the endocytosis process for one single capsule as quantified in terms of Δ*t*_A_ and Δ*t*_P10 %_ is significantly faster in case of serum‐deprived culture. This is in line with general findings by other groups.19c–19e The same can be also seen in the (|d(*I*_r_/*I*_y_)/dt|)_max_ data, which show faster acidification for serum‐free culture. It is also striking that behavior of *t*_c_ and Δ*t*_A_ goes in the opposite direction. Note, that these statements are corroborated by quantitative values. This also allows the statistical relevance of these data to be looked at. Error bars for all the data are given in the Supporting Information. As the results are quantitative, in principle values can be compared between different studies. In a previous article, the same method was applied to similar particles (2 bilayers of poly(sodium 4‐styrenesulfonate), and poly(allylamine hydrochloride) (PSS/PAH), template core diameter *d*_c_≈4 μm, positively charged (“+”), HeLa cells, serum supplemented culture (“w”)), resulting in the following values: Δ*t*_A_≈24 min, Δ*t*_P10 %_≈30 min (note that a different nomenclature had been used and the values were named *t*_A_ and *t*_P10 %_).^34^ Comparison with the values from the present study shown in Table 1 demonstrates, that the absolute values, in particular for *t*_P10 %_ are different. This again shows the difficulty in obtaining absolute values, as slight variations in respective methods may change the absolute values. Best statements can be obtained by comparing the relative values within one series of different particle/cell combinations, which have been carried out under exactly the same method within one study. Summarizing this part, pH‐sensitive single‐particle tracking provides quantitative results. For example, how fast the internalization of one particle is, that is, a quantification in terms of time. As only single particles are analyzed, there is no information to be gained about the quantity of particles which are internalized per cell. Geometrical analysis of the particle trajectories in terms of *D* and *ν* did not lead to significant changes for the different signs of capsule charge and serum‐supplemented versus serum‐free culture.

**Table 1:** Experimental data obtained with HeLa cells and polyelectrolyte capsules (2 and 2.5 bilayers of poly(sodium 4‐styrenesulfonate), and poly(allylamine hydrochloride) (PSS/PAH), resulting in positively (“+”) and negatively (“−”) charged capsules, hydrodynamic diameter *d*_h_≈3.5 μm).^[a]^

Variables			Single‐particle tracking results					
charge	serum	*N*_caps/cell_ (added)	*t*_c_ [min]	Δ*t*_A_ [min]	Δ*t*_P50 %_ [min]	Δ*t*_P10 %_ [min]	*t*_50 %_ [min]	(|d(*I*_r_/*I*_y_)/dt|)_max_ [min^−1^]
+	w	5	92	45	257	234	349	0.06
+	w/o	5	49	25	110	95	159	0.10
−	w	5	19	43	305	287	324	0.09
−	w/o	5	24	16	74	64	98	0.24

variables			single particle tracking results					
charge	serum	*N*_caps/cell_ (added)	*D*_out_	*D*_uptake_	*D*_in_	*ν*_out_	*ν*_uptake_	*ν*_in_
+	w	5	1.31	1.29	1.30	0.65	0.68	0.64
+	w/o	5	1.32	1.38	1.38	0.66	0.61	0.51
−	w	5	1.34	1.32	1.35	0.61	0.64	0.60
−	w/o	5	1.32	1.32	1.34	0.63	0.65	0.55

[a] Capsules as model particles of this “big” size have been chosen on purpose, as they can be conveniently resolved by optical microscopy. *N*_caps/cell_(added)=5 were added per HeLa cell under serum‐supplemented (“w”) and serum‐free (“w/o”) culture conditions. The geometrical data analysis of the particle trajectories resulting in the parameters *D* and *ν* was carried out for three different time regions: when the particles were still outside the cells (“out”), during the internalization process (“uptake”), and after internalization (“in”). Experimental details are provided in the Supporting Information.

## 6 Particle Uptake as Analyzed by Particle Counting

**Figure 3:**
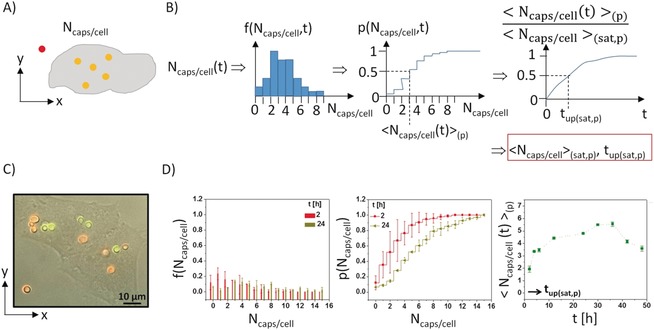
A) The number of internalized particles per cell *N*_caps/cell_ is obtained individually for a number of different cells for each time point of incubation, for example, from overlays of bright field and fluorescence microscopy images. Labelling with pH‐sensitive dyes allows to distinguish internalized particles (shown in yellow) from extracellular particles (shown in red). B) From the counted particles per cell data, a histogram can be made for each time point, whereby *f*(*N*_caps/cell_,*t*) corresponds for the frequency of cells with *N*_caps/cell_ internalized particles. The histograms can be converted into cumulative distribution function *p*(*N*_caps/cell_,*t*). The mean number of internalized particles per cell can be either derived from the histograms (<*N*_caps/cell_(*t*)>_(h)_) or from the cumulative distribution function, where *p*=0.5 (<*N*_caps/cell_(*t*)>_(p)_).19a Plotting the mean number of internalized particles per cell versus time allows the maximum number of internalized particles per cell to be determined (i.e. under saturation conditions; <*N*_caps/cell_(*t*)>_(sat,h)_ and <*N*_caps/cell_(*t*)>_(sat,p)_), as this is the time it takes cells to reach half saturation with the internalized particles (*t*_up(sat,h)_ and *t*_up(sat,p)_). C) HeLa cells incubated with capsules with integrated pH‐sensitive fluorophores as model particles, showing an overlay of phase contrast and the two (*I*_y_, *I*_r_) fluorescence channels in false colors (yellow, green). These data were obtained for positively charged capsules in the presence of serum and for *N*_caps/cell_(added)=10 capsules added per cell after 2 h of incubation. For details see Table 2 and the Supporting Information. D) Histograms and cumulative distribution functions for the positively charged capsules for which an image has been shown in (C), after 2 and 24 h of incubation. The following data were obtained from these graphs: <*N*_caps/cell_(*t*=2 h)>_(h)_=2.9, <*N*_caps/cell_(*t*=24 h)>_(h)_=6.0, <*N*_caps/cell_(*t*=2 h)>_(p)_=1.9, <*N*_caps/cell_(*t*=24 h)>_(p)_=4.8. The time‐dependent data are normalized to the maximum number of internalized capsules per cell. From these graphs the maximum number of internalized capsules per cell and the time it takes a cell to uptake the number of capsules under saturation conditions are derived. The values derived for the shown graphs are <*N*_caps/cell_>_(sat,p)_=5.6, *t*_up(sat,p)_=3 h, *t*_up(1)_=<1 h. The same scaling for the *f*(*N*_caps/cell_,*t*) and *p*(*N*_caps/cell_,*t*) is used to visualize that *p*(*N*_caps/cell_,*t*) is formed by consecutively summing up the *f*(*N*_caps/cell_,*t*) values.

In the case of particles being big enough that they can be laterally resolved, the amount of particles internalized per cell *N*_caps/cell_ can be counted from images, in which the cells as well as the particles can be seen. Such images can be recorded, for example, with transmission electron microscopy (TEM),^46^ optical microscopy (phase contrast, scattering), fluorescence microscopy,1b, 33a, 33b, 47 focused ion beam (FIB)/scanning electron microscopy (SEM),^48^ or other microscopy techniques. Note that here the incubation conditions in terms of particle dose *N*_caps/cell_(added) need to be chosen differently than in the case of single particle tracking. As in this case the amount of internalized particles will be quantified, cells need to be exposed to a larger number of particles. Still, this method can be only applied when the average distance between the internalized particles is significantly larger than the lateral resolution of the used microscopy technique. In addition, as cells are three‐dimensional (3D) objects, in particular for small particles, the number of counted particles in 2D microscopy images is the number of particles per cross section of the cell, rather than the whole number of particles per cell. TEM allows for the best lateral resolution of the above mentioned microscopy techniques, and thus also small nm‐sized (as a result of contrast issues in particular inorganic) particles can be recorded. Cell membranes also can be stained to provide contrast, and because of the high lateral resolution it is possible to distinguish intracellular from extracellular particles. Thus the number of internalized particles per cell can be counted.^49^ As TEM images need to be recorded in vacuum and samples need to be thin (i.e. slices of resin‐embedded cells), it is hard to determine the number of particles across a whole cell, and thus stereological methods can be used.^50^ TEM conveniently allows the intracellular locations of particles to be determined, but for quantitative analysis of the amount of internalized particles per cell it is not the most efficient method. However, future development in automated microtomes for cell slicing, followed by TEM imaging and three‐dimensional image reconstruction, would certainly boost analysis of quantitative particle uptake by TEM. Optical imaging allows for higher throughput. In case of small nm‐sized particles, because of the optical resolution limit it is not possible to resolve single particles with standard microscopy techniques (super‐resolution microscopy could circumvent this problem). Thus, for small particles, instead of counting the number of internalized particles per cell, also the number of intracellular vesicles loaded with particles can be counted.^51^ When particles are big enough, the number of internalized particles per cell *N*_caps/cell_ can be directly counted,^13^, 19a, 33a, 33b, 52 see Figure 3 A. This also allows the percentage of internalized particles *N*_caps/cell_/*N*_caps/cell_(added) to be determined.^53^ As discussed above, the use of pH‐sensitive fluorophores27c or immunostaining^13^ of endosomes/lysosomes helps to distinguish internalized from extracellular particles. Alternatively, internalization of capsules has also been verified by their mechanical deformation.52a, 54 From such histograms, in which the observed frequency *f*(*N*_caps/cell_) that a cell has internalized *N*_caps/cell_ particles is plotted versus *N*_caps/cell_, the cumulative probability/cumulative distribution function (CDFs) *p*(*N*_caps/cell_) can be calculated,^13^, 33a see Figure 3 B. In this case, *p*(*N*_caps/cell_) is the probability that a cell has internalized not more than *N*_caps/cell_ particles per cell. By obtaining the mean number of internalized particles per cell for a given incubation time from the *f*(*N*_caps/cell_,*t*) or *p*(*N*_caps/cell_,*t*) plot, the mean number of internalized particles per cell can be plotted over time, see Figure 3 B.19a For saturation‐like behavior, the maximum number of internalized particles per cell <*N*_caps/cell_>_(sat)_ can be derived. From the kinetics, the time *t*_up(sat)_ it takes cells to incorporate the saturation amount of particles per cell can be determined, see Figure 3 B. From the *p*(*N*_caps/cell_) graphs for different times the average time until cells have internalized at least one capsule *t*_up(1)_ can also be calculated, see the Supporting Information. In Figure 3 D, experimental data for the uptake of polyelectrolyte capsules as model particles is provided as example. The extracted parameters are summarized in Table 2.

**Table 2:** Experimental data obtained with HeLa cells and polyelectrolyte capsules (2 and 2.5 bilayers of PSS/PAH, resulting in positively (“+”) and negatively (“−”) charged capsules, hydrodynamic diameter *d*_h_≈3.5 μm).^[a]^

Variables			Particle‐counting results				
charge	serum	*N*_caps/cell_ (added)	<*N*_caps/cell_>_(sat,p)_	*t*_up(sat,p)_ [h]	<*N*_caps/cell_>_(sat,h)_	*t*_up(sat,h)_ [h]	*t*_up(1)_ [h]
+	w	10	5.6	3	6.5	3	<1
+	w	20	6.9	6	7.9	<2	<1
+	w/o	10	3.5	2	4.5	1	<1
+	w/o	20	5.0	1	6.0	<2	<1
−	w	10	2.3	2	3.2	<2	<1
−	w	20	3.9	1	5.3	<2	<1
−	w/o	10	2.3	2	3.2	<2	<1
−	w/o	20	4.6	1	5.6	<2	<1

[a] *N*_caps/cell_(added) were added per HeLa cell under serum‐supplemented (“w”) and serum‐free (“w/o”) culture conditions. The derived parameters are explained in Figure 3. Note that in the Tables and Figures error bars are sometimes omitted to avoid overloading. However, all error bars are provided in the Supporting Information.

One important internal control of particle uptake experiments is to verify that the particle dose has been selected low enough to be in the “(quasi‐)linear” regime, that is, on doubling the particle concentration *N*_caps/cell_(added) under saturation conditions twice the amount of particles <*N*_caps/cell_>_(sat)_ is internalized. From Table 2 it can be seen that by doubling the capsules’ concentration from *N*_caps/cell_(added)=10 to 20, the maximum amount of internalized capsules <*N*_caps/cell_>_(sat)_ increased. In particular in case of the positively charged capsules it did not double, but there is a clear concentration dependence. Using different metrics, that is, analyzing the numbers from the histograms (<*N*_caps/cell_>_(sat,h)_) or from the cumulative distribution function (<*N*_caps/cell_>_(sat,p)_), does not provide exactly the same absolute values, but the same tendency can be clearly observed. Serum in the medium (“w”) in comparison to serum‐free medium (“w/o”) did not change the maximum number of internalized capsules <*N*_caps/cell_>_(sat)_, in particular for the negatively charged capsules, but it increased the time *t*_up(sat)_ until cells had internalized capsules to 50 % saturation, in particular for the positively charged capsules. More of the positively charged capsules (“+”) were internalized under saturation (i.e. higher <*N*_caps/cell_>_(sat)_ values) as compared to negatively charged ones (“−”). Positively charged capsules were also internalized slower (in terms of *t*_up(sat)_), in particular under serum‐containing conditions.

These findings are comparable to the results from single‐particle tracking in Table 1. Positively charged capsules take longer to stick to a cell (*t*_c_). The actual time needed for the internalization of one capsule (Δ*t*_A_) does not depend on the charge, resulting in total a longer time until one capsule is internalized (*t*_50 %_) as measured from the time point of incubation. The particle counting experiments indicate that for the positively charged capsules it takes longer to saturate a cell (*t*_up(sat)_), that is, uptake is slower, but in total more positively charged capsules can be internalized (<*N*_caps/cell_>_(sat)_). Saturation of cells with capsules occurred in the time scale of a few hours. These findings are also compatible with a previous study, in which in A549 cells, more positively charged capsules were found to be internalized than negatively charged ones,19a but in the case of HeLa cells there was no large effect of charge. In these previous results more added capsules also resulted into more internalized capsules.19a Data of 24 h versus 12 h incubation showed no clear ongoing rise of internalized capsules per cell over time,19a that is, saturation is already reached after a few hours of incubation.

## 7 Particle Uptake Analyzed by Determining the Mean Signal per Cell

This is probably the most commonly used methodology for quantifying the particles’ uptake by cells. It is based on measuring the overall signal originating from particles per cell. There are different options for obtaining “signals” from particles. If particles are fluorescent, then the mean fluorescence per cell <*I*_y_(*t*)> can be recorded. This can be done, for example, with fluorescence microscopy, followed by analyzing the mean fluorescence per cell. Note that in contrast to the previous Section about single‐particle counting, no resolution of individual particles is required. With this technique the mean fluorescence over time can be analyzed, see Figure 4.19b, 55 The trick for comparability of different studies is in the details of the analyzation methodology. Either the fluorescence of individual cells can be determined, and then the mean value is obtained, or the fluorescence of all cells in one image can be determined, and the mean fluorescence per cell is then derived by normalization with the number of cells in the image. The background subtraction plays a crucial role. To quantify the amount of internalized particles by fluorescence, the background fluorescence of the cells and culture substrate need to be subtracted, which if not done properly leads to false positive counts, in particular when dealing with particles with low fluorescence and low exposure doses. Distinguishing between internalized and extracellular particles is possible, for example, as already mentioned for the other methods with pH sensitive dyes,^56^ or by correlation with immunostaining.24b, 55c Here, only the fluorescence of particles which overlaps laterally with the fluorescence of immunostained intracellular organelles, in particular endosomes and lysosomes, will be counted.24b, 55c The degree of co‐localization can be quantified with Manders coefficients.7e, 57 For this correlation, lateral resolution is required because analysis can be only performed on images acquired from the microscope, but not on flow cytometry data (which will be explained below).

**Figure 4:**
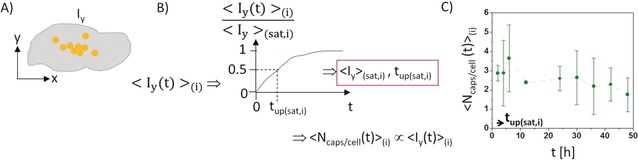
A) The “signal” of particles in a cell, which can be fluorescence (*I*_y_) or mass of element X (*m*_X_) is detected per cell. Individual particles do not need to be laterally resolved for this purpose. B) The particle signal associated with cells (*I*_y_ in case of fluorescence, *m*_X_ in case of the elemental analysis of element X) is recorded for many cells at different time points *t*, leading to the average time‐dependent signal intensity per cell <*I*_y_(*t*)>. From this, the maximum signal intensity <*I*_y_>_(sat)_ under saturation conditions can be obtained, as well as the time *t*_up_(sat) to reach half saturation. If the signal intensity per particle is known, then the particle signal per cell <*I*_y_> can be converted into the number of particles per cell <*N*_caps/cell_>. C) As an example, the internalization of capsules with integrated Au nanoparticles in their shells by HeLa cells is shown. These data were obtained for positively charged capsules in the presence of serum for *N*_caps/cell_(added)=10 capsules added per cell. For details see Table 3 and the Supporting Information. The mean amount of elemental Au per cell <*m*_Au_> was determined with ICP‐MS. As the amount of elemental Au per capsule is known, this could be converted into the mean number of internalized capsules per cell <*N*_caps/cell_>. These data are plotted for different incubation times. The following data were obtained from this graph: <*N*_caps/cell_>_(sat,.i)_=3.6, *t*_up(sat,i)_=<2 h. A summary of data obtained with this method is presented in Table 3.

**Figure 5:**
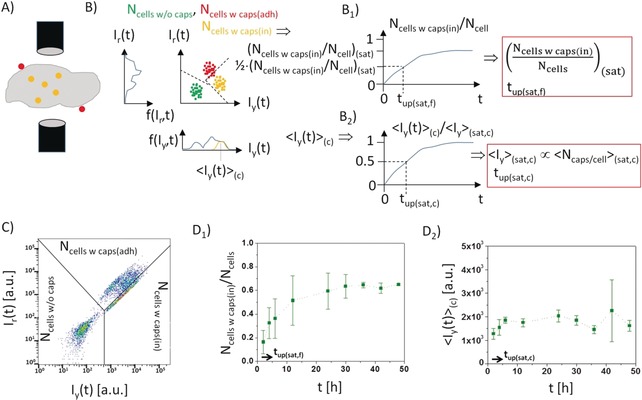
A) Cells in a flow channel pass a fluorescence detector one at a time, and for each event the fluorescence intensity is recorded. For ratiometric pH‐sensitive fluorophores, fluorescence is detected in the yellow (*I*_y_) and in the red (*I*_r_) channel, corresponding to acidic and neutral local pH. B) All events are plotted, and different populations can be identified. Cells without associated particles have low fluorescence in both channels (depicted as “green” population). Cells with adherent and internalized particles have predominant red and yellow fluorescence due to neutral and acidic local pH, respectively, around the particles. The number of cells measured for each of the populations can be extracted as *N*_cells w/o caps_, *N*_cells w caps(adh)_, and *N*_cells w caps(in)_. In practice, the populations may overlap and need to be distinguished by defining proper gatings. From the populations the mean fluorescence intensity of internalized particles per cell <*I*_y_> can also be derived. B_1_) By plotting the percentage of labelled cells versus time (*N*_cells w caps(in)_(*t*)/*N*_cells_) with *N*_cells_=*N*_cells w/o caps_ + *N*_cells w caps(adh)_ + *N*_cells w caps(in)_, the maximum percentage of labelled cells under saturation conditions (*N*_cells w caps(in)_/*N*_cells_)_(sat)_, and the time it needs to reach half saturation *t*_up(sat,f)_ can be calculated. B_2_) The mean fluorescence per cell can also be plotted over time, leading to the fluorescence under saturation conditions <*I*_y_>_(sat,c)_ and the time *t*_up(sat,c)_ it needs to reach half saturation. C) Population plot obtained for HeLa cells incubated with positively charged capsules in the presence of serum for *N*_caps/cell_(added)=10 capsules added per cell. D) Plots of the percentage of labelled cells and the mean fluorescence intensity per cell versus time. From these graphs the following parameters were extracted: (*N*_cells w caps(in)_/*N*_cells_)_(sat,f)_=65 %, *t*_up(sat,f)_=4 h, <*I*_y_>_(sat,c)_=2040, and *t*_up(sat,c)_=<2 h. The parameters of the total data set are presented in Table 3 and Table 4.

In principle, from the single‐particle counting studies, the mean fluorescence per cell can be obtained by measuring the fluorescence of one particle, and by calculating the fluorescence per cell as the number of internalized particles per cell times the fluorescence per particle. Note that in this case there is no background from cells in the calculated fluorescence intensities. Thus, the absolute values may differ from those which are directly calculated by measuring the background‐corrected mean fluorescence per cell. In addition, the mean fluorescence per cell <*I*_y_(*t*)> after different incubation times can be determined by flow cytometry, see Figure 5.^56^ In this case the fluorescence per cell is determined at the level of individual cells and the mean fluorescence per cell is calculated as the mean value of the discrete measurements. Flow cytometry allows for high throughput, that is, mean values based on many cells, and provided statistically more significant data than the analysis of cells by microscopy, in which typically only a few cells are analyzed per data point. As background, the fluorescence from cells without exposure to particles needs to be subtracted. Flow cytometry does not allow for lateral resolution, but internalized particles can be distinguished from extracellular particles by the use of pH‐sensitive fluorophores.^21^, ^56^ Note that flow cytometry data may also be analyzed with a different metrics, as described below.

The “particle signal” in cells can also be determined by elemental analysis. In the case of Au nanoparticles, for example, the amount of internalized particles can be expressed as mean mass of Au “*m*_Au_” which is found per cell, see Figure 4. A typical method used with inorganic particles is inductively coupled plasma mass spectroscopy (ICP‐MS) or related techniques.8b, 55b, 58 As ICP‐MS typically does not implement lateral resolution, in fact the amount of the element from which the particles are composed of is determined within a cell pellet, and this mass is then normalized by the number of cells in the pellet to derive the mean elemental mass *m*_X_ of element X per cell. ICP‐MS requires that the element to be detected can be measured by the ICP‐MS set‐up used, which is possible for most of the metals. If the element naturally occurs in cell, such as iron, then this background needs to be subtracted from the overall signal.

In principle, also any other “particle signal” can be used for this type of study. There is very early work in this direction, for example, based on radioactivity,^59^ which even involved distinguishing between intracellular and extracellular particles based on washing methods.

Again, the results shown in Table 3 are compatible with the data obtained by the other methods shown in Table 1 and Table 2. At the capsule concentrations used, uptake is in the quasi linear regime, that is, doubling the amount of added capsules per cell (*N*_caps/cell_(added) from 10 to 20) leads to almost the double number of internalized capsules (<*N*_caps/cell_>_(sat,i)_ and <*I*_y_>_(sat,c)_). Absence of serum (“w/o” versus “w”), in particular in the case of the negatively charged capsules, leads to a higher number of internalized capsules under saturation conditions. Concerning the comparison of the positively and negatively charged capsules, the statement is more complicated, as the experimental results are based on “signal” derived from the internalized particles, which has to be converted into the number of internalized particles by multiplication with a scaling factor. In case of ICP‐MS measurements, the scaling factor was the mass of elemental Au per capsule. The results show, that under all conditions under saturation there are more positively than negatively charged capsules internalized per cell (<*N*_caps/cell_>_(sat,i)_). For flow cytometry, the scaling factor for converting the fluorescence intensity due to internalized capsules into the number of internalized capsules would be the fluorescence intensity per capsule. In this case the negatively charged capsules were brighter than the positively charged capsules (cf. the Supporting Information), which explains the higher intracellular fluorescence of cells upon exposure to negatively charged capsules (<*I*_y_>_(sat,c)_). The calibration by the fluorescence intensity per capsule is however, prone to errors. There is, for example, pH‐dependent quenching of many fluorophores, and thus the emission intensity of the capsules will depend on their location.^60^ The scaling factor also depends on the set‐up parameters used, such as the intensity of excitation. Quantification of the absolute number of internalized capsules thus has to be interpreted with care.

**Table 3:** Experimental data obtained with HeLa cells and polyelectrolyte capsules (2 and 2.5 bilayers of PSS/PAH, resulting in positively (“+”) and negatively (“−”) charged capsules).^[a]^

Variables			ICP‐MS results		flow cytometer results	
charge	serum	*N*_caps/cell_ (added)	<*N*_caps/cell_>_(sat,i)_	*t*_up(sat,i)_ [h]	<*I*_y_>_(sat,c)_ [a.u.]	*t*_up(sat,c)_ [h]
+	w	10	3.6	<2	2042	<2
+	w	20	6.8	<2	4027	2
+	w/o	10	4.3	<2	2342	<2
+	w/o	20	7.0	<2	5124	3
−	w	10	2.5	<2	7228	3
−	w	20	5.2	<2	10 131	4
−	w/o	10	3.7	<2	8678	<2
−	w/o	20	6.4	<2	9867	<2

[a] *N*_caps/cell_(added) were added per HeLa cell under serum‐supplemented (“w”) and serum‐free (“w/o”) culture conditions. In case of the ICP‐MS data the hydrodynamic diameter was *d*_h_≈3 μm. The capsules were labelled with gold nanoparticles and the elemental mass of Au per capsule was determined by ICP‐MS. Using this calibration allowed the mean number of internalized capsules per cell to be measured under saturation conditions <*N*_caps/cell_>_(sat,i)_ by measuring elemental masses of Au. The derived parameters are explained in Figure 4. For flow cytometry measurements, the capsules were loaded with the pH‐sensitive fluorophore SNARF. The hydrodynamic diameter was *d*_h_≈3.5 μm. Data show the mean fluorescence intensities <*I*_y_>_(sat,c)_ under saturation conditions, of the fluorescence channel corresponding to the capsules which have been internalized (i.e. are surrounded by acidic pH). The derived parameters are explained in Figure 5.

Particle uptake as analyzed by determination of the mean signal per cell works well for quantifying the relative amount of internalized particles, that is, for the comparison of the particle‐mediated intracellular “signal” for different types of particles. Absolute values are more complicated to obtain owing to the need of a conversion factor relating the “signal” of internalized particles to the number of internalized particles. For quantification of the speed of uptake this method is not the best suited, as typically data is collected and quantified only for limited time points (i.e. exposure times). In addition, proliferation and exocytosis dilute the number of internalized particles. To reach the saturation condition, longer time scales (i.e. incubation times) need to be measured, at which proliferation as well as exocytosis come into effect. In the data set presented herein, no useful time parameters (*t*_up(sat)_) could be extracted. The single‐particle tracking experiments on the other hand only consider the uptake of individual particles and thus proliferation and exocytosis do not need to be considered.

## 8 Particle Uptake Analyzed by Percentage of Cellular Labelling

While using flow cytometry for deriving the mean particle‐associated fluorescence per cell (<*I*_y_>) has been discussed in the previous Section (cf. Figure 5 and Table 3), flow cytometry data can also be interpreted differently. Instead of focusing on the particles (i.e. the number of internalized particles), focus is put on the cells in terms of the number of labelled cells *N*_cells w caps(in)_. The essential difference lies in the background correction. Flow cytometry identifies different fluorescence “events” passing the fluorescence detector. In this way different populations can be identified, such as free particles, cells without particles, cells with adherent particles, and cells with internalized particles, see Figure 5 A.^21^ To identify these different populations “gating” is applied. Quantitative analysis is predominantly influenced by the selected gating parameters. Thus, it is paramount that the gating strategy is sufficiently explained for each study. By plotting the percentage of particle‐labelled cells versus time (*N*_cells w caps(in)_/*N*_cells_), the time *t*_up(sat,f)_ can be determined, which is the time it takes for 50 % of the maximum labelling of cells has been reached, see Figure 5 B_1_. Experimental data is shown in Figure 5 and Table 4.

**Table 4:** HeLa cells had been incubated with polyelectrolyte capsules (2 and 2.5 bilayers of PSS/PAH, resulting in positively (“+”) and negatively (“−”) charged capsules).^[a]^

Variables			Flow cytometer results	
charge	serum	*N*_caps/cell_ (added)	(*N*_cells w caps(in)_ /*N*_cells_)_(sat,f)_ [%]	*t*_up(sat,f)_ [h]
+	w	10	65	4
+	w	20	62	6.5
+	w/o	10	64	5
+	w/o	20	56	6
−	w	10	69	4
−	w	20	78	5
−	w/o	10	68	3
−	w/o	20	73	3

[a] *N*_caps/cell_(added) were added per HeLa cell under serum‐supplemented (“w”) and serum‐free (“w/o”) culture conditions. Data were extracted as shown in Figure 5 B_1_.

For many applications the percentage of labelled cells^21^, ^53^ is an important parameter, such as for transfection studies, which are based on an all‐or‐nothing effect, that is, either a cell is sufficiently transfected or not. For quantitative studies of particle uptake this metric has severe limitations, as it does not provide any information about the degree of labelling of cells. As shown in Table 4 under saturation, for all conditions the percentage of labelling of cells with particles is similar. However, as shown in Table 3 the degree of labelling, that is, the amount of intracellular fluorescence within labelled cells, varies. The time it takes labelling to reach saturation is of practical importance. However, to extract a reliable time parameter *t*_up(sat,f)_, many different incubation time points would have to be investigated, and interpretation is complicated by cell proliferation and exocytosis of internalized particles. For quantitative uptake studies thus the methods described in the previous paragraphs are more suitable.

## 9 Discussion

Decades after the popularization of nanotechnology, conclusions in the style of “certain particles are incorporated by certain cells” do no longer contain significant new information. In the meantime, early work^61^ that virtually all different types of particles are endocytosed by cells, has been confirmed by hundreds of groups.33a, 61a, 62 It is also well accepted that a particle entering a cell by a different pathway to the many routes of endocytosis, is an exception. An image of particles endocytosed by cells thus does not provide much information. In fact, novel information regarding the quantification of uptake can only be gained with quantitative analysis. In this Minireview several such methodologies have been discussed. Even such analysis is not new and has already been presented many decades ago.^59^ In addition, as proteins may be considered as colloidal particles, a large data set of protein uptake studies by cells over the last decades must also be taken into account, in which also the influence of different parameters, such as charge have been quantitatively studied.^63^

Having a look at the presentation of the example data in this study is somewhat discouraging. From the data there is the clear statement that working with appropriate particle doses, increase in particle concentration results in increase in the amount of internalized particles. Serum depleted incubation results in faster internalization of particles rather than in an increased number of internalized particles under saturation conditions. More of the positively charged capsules (“+”) were internalized under saturation (i.e. higher <*N*_caps/cell_>_(sat)_ values) as compared to negatively charged ones (“−”). Positively charged capsules were also internalized slower (in terms of *t*_up(sat)_), in particular under serum‐containing conditions, as they take longer to stick to a cell (*t*_c_). Differences in particular related to charge were rather small. This may be due to the fact that the capsules used were micrometer sized and big, and thus differences in their surface properties had less effect on cellular uptake as, for example, for smaller nanoparticles.19b Recently some studies showed that in fact relevance of surface properties is less important for particles of bigger size.^64^ This may be due to the effect that in static uptake studies with two‐dimensional assemblies of adherent cells, in particular basic physical parameters, such as density and size of the particles, dominate as they define the interplay between particle diffusion and sedimentation.2a We want to point out that there are datasets from which more significant conclusions can be drawn, but on purpose we wanted to show a standard data set to point out common limitations.

To extract reliable quantitative parameters the following points should be considered:

detailed description of the metrics used to quantify particle doses
physicochemical characterization which in particular probes the potential agglomeration under the exposure conditions
verification that under the exposure conditions used there is no acute toxicity (e.g. no significant reduction in cell viability)
recording of concentration dependence, that is, demonstrating that higher particle doses leads to higher particle uptake
recording of a time series based on different exposure times
taking into account in the analysis that the respective detection method might wrongly count particles adsorbed to the outer cell membrane as being internalized particles (i.e. false positives)
trying to extract quantitative parameters: a time parameter that describes how fast particles are incorporated and a quantification parameter that describes how many particles are internalized under saturation conditions
attempt of making direct comparisons of particles with different properties under the same experimental condition

The best statements can be obtained in cases in which several methods of quantification are applied. Regarding kinetics, the single‐particle tracking experiments are convenient to perform. Concerning the determination of the maximum amount of internalized particles under saturation, methods which quantify the particles’ associated “signal” per cell, such as fluorescence or elemental mass of particles are convenient to perform. The most significant statements can be obtained when the behavior of different particles is directly compared.

In vitro particle studies are a good tool to demonstrate different particles’ uptake behavior based on different physicochemical properties of the particles. Differences between different types of particles, however, should not be exaggerated. They cannot be determined with absolute precision, as there is significant influence of the experimental settings chosen. Without proper analysis considering time‐ and concentration dependence, the gain of new knowledge by particle uptake studies by cells is very limited.

There is a clear need to improve theoretical models, which would lead to fits of experimental data leading to quantitative parameters.^20^, ^30^, ^65^ Also data‐mining approaches, as used for example for toxicity studies^66^ should help to extract more quantitative data from the overwhelming experimental dataset available in the literature.

## Conflict of interest

The authors declare no conflict of interest.

## Biographical Information

*Sumaira Ashraf obtained her PhD degree from the Quaid‐i‐Azam University*, *Islamabad, Pakistan, with one year at Philipps University of Marburg. She was appointed Assistant Professor at the Pakistan Institute of Engineering and Applied Sciences, Nilore, Islamabad, Pakistan and held two postdoctoral positions in the Biophotonics group in Marburg with Prof. Dr Wolfgang J. Parak (Alexander von Humboldt fellowship), and in Liverpool with Dr Raphaël Lévy (Marie Curie Fellowship). Recently she joined the Institute of Industrial Biotechnology, Government College University, Lahore as Associate Professor*.

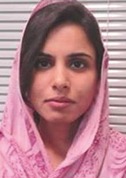

## Biographical Information

*Wolfgang Parak studied physics at the TU München and obtained his PhD with Hermann Gaub at the LMU München, working on cell–semiconductor interfaces. As a postdoc he joined the Chemistry Department of UC Berkeley with Paul Alivisatos, (bioconjugation and applications of colloidal nanoparticles). He started his own group as Emmy Noehter fellow at the CENS at the LMU München. He was a Full Professor in the Physics Department of the Philipps Universität Marburg for 10 years. In 2017 he moved to the Universität Hamburg as Full Professor at CHyH at the Physics Department, with co‐affiliation in Chemistry. He is also a group leader at CIC Biomagune in San Sebastian and Associate Editor of ACS Nano*.

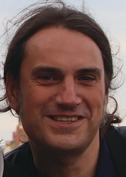

## Supporting information

As a service to our authors and readers, this journal provides supporting information supplied by the authors. Such materials are peer reviewed and may be re‐organized for online delivery, but are not copy‐edited or typeset. Technical support issues arising from supporting information (other than missing files) should be addressed to the authors.

Supplementary
