# Supplementary material for: Quantitative Particle Uptake by Cells as Analyzed by Different Methods
Source: Angew Chem Int Ed Engl. 2019 Dec 13;59(14):5438–53. doi: 10.1002/anie.201906303 (PMC7155048; doi:10.1002/anie.201906303)
Supplement: Supplementary file 1 — Supplementary [file ANIE-59-5438-s001.pdf]

Angewandte Chemie
Eine Zeitschrift der Gesellschaft Deutscher Chemiker

Supporting Information

Quantitative Particle Uptake by Cells as Analyzed by Different Methods

*Sumaira Ashraf, Alaa Hassan Said, Raimo Hartmann, Marcus-Alexander Assmann, Neus Feliu, Peter Lenz, and Wolfgang J. Parak**

anie_201906303_sm_miscellaneous_information.pdf

Supporting Information

I) Particle tracking of SNARF-loaded capsules inside cells
II) Uptake studies of SNARF-loaded capsules by cells using fluorescence microscopy
III) Flow cytometer analysis of SNARF-loaded capsules internalized by cells
IV) Elemental analysis of gold nanoparticle (GNP)-loaded capsules internalized by cells
V) Viability measurements of cells exposed to capsules and inhibitor
VI) References

Note that excerpts of the results shown in the graphs shown in the Supporting Information are also presented in a different representation/compilation in the main manuscript.

I) Particle tracking of SNARF-loaded capsules inside cells

I.1) Materials, reagents, and equipment
I.2) Synthesis and characterization of SNARF-loaded polyelectrolyte micro- (PEM) capsules
I.3) Uptake studies by confocal laser scanning microscope (CLSM)
I.4) Particle tracking by CLSM, image processing, and data evaluation
I.5) Fractal dimension and average end-to-end scaling exponent
I.6) Results

I.1) Materials, reagents, and equipment

Calcium chloride dihydrate ($CaCl_2$, #223506), sodium carbonate (Na_2CO_3, #S7795), sodium chloride (NaCl, # S7653), poly(sodium 4-styrenesulfonate) (PSS, molar weight $M_w \approx 70$ kDa, #243051), poly(allylamine hydrochloride) (PAH, $M_w \approx 58$ kDa, #283223), ethylenediaminetetraacetic acid disodium salt dihydrate (EDTA disodium salt, #E5134), cytochalasin D (#C8273), bafilomycin A1 (#B1793), and sterile dimethyl sulfoxide (DMSO, #D2650) were purchased from Sigma-Aldrich (Germany). Seminaphtharhodafluor-1 dextran (SNARF-1 dextran, $M_w \approx 70$ kDa, #D3304) was obtained from Life Technologies (USA) and were supplied from Roth (Karlsruhe, Germany). Ultrapure double distilled deionized (Milli-Q) water (DDW) having resistivity of 18.2 MΩ.cm was used for all experiments.

Human cervical carcinoma (HeLa) cells were obtained from American type culture collection (ATCC). For cell culture Dulbecco's modified Eagle's medium (DMEM; #D6546), and penicillin streptomycin (P/S, #P4333) were purchased from Sigma-Aldrich. GlutaMAX™ (#35050-038) and 0.05% Trypsin-EDTA (#25300) were purchased from GIBCO (Life Technologies). Phosphate buffered saline (PBS; Biochrom #L 1825) and fetal bovine serum (FBS; Biochrom, Germany, #S0615) were purchased from Merck Millipore. A Neubauer improved counting chamber (haemocytometer) by Marienfeld Laboratory glassware was used for counting cells. µ-slide 8 well plates (Ibidi #80826) were purchased from Ibidi. Cell culture flasks (25 and 75 cm^2; #90025 and #90075, respectively) from TPP were used for culturing cells. Eppendorf (2 mL; # 72.695.500), and falcon tubes (15 and 50 mL; #62.554.502, #62.547.254, respectively) were from Sarstedt. A thermo electron corporation Varifuge 3.0 R by Fisher Scientific was used for centrifuging the cell lines. The hydrodynamic diameter and zeta potential measurements of the capsules were performed in water using a Malvern Zetasizer Nano ZS.

A confocal laser scanning microscope (CLSM 510 Meta) from Zeiss was used for visualizing and live imaging of HeLa cells engulfing SNARF-loaded capsules. For image acquisition the fluorescence was excited at 543 nm using the helium neon laser of the CLSM and samples were observed through a 63X/1.40 oil-immersion DIC M27 objective. ImmersolTM 518F immersion oil (Zeiss) was used during imaging.

I.2) Synthesis and characterization of SNARF-loaded polyelectrolyte micro- (PEM) capsules

PEM capsules with encapsulated SNARF dextran inside their cavities were fabricated by co-precipitation, followed by layer by layer (LBL) assembly of oppositely charged polymeric layers [1]. Briefly, pH-sensitive PEM capsules having 2 and 2.5 bilayers of non-biodegradable polymers composed of PSS and PAH were fabricated based on LBL assembly of oppositely charged polymers around sacrificial template cores containing the pH sensitive dye SNARF-1 conjugated with dextran. For co-precipitation of SNARF-1 dextran with $CaCO_3$ microparticles, solutions of $CaCl_2$ and Na_2CO_3 were mixed under vigorous stirring in the presence of SNARF-1 dextran at room temperature (RT) in aqueous media. In a glass vial, 4.2 mL SNARF-1 dextran (0.5 mg/mL) was added to 3 mL of 0.33 M $CaCl_2$ (0.33 M). Under vigorous magnetic stirring (1100 rpm) 3 mL of Na_2CO_3 (0.33 M) solution was quickly mixed with the above mixture for 30 s, followed by keeping the reaction contents without agitation for 2 min. Calcium carbonate particles were washed three times with DDW and used for LBL assembly of oppositely charged polyelectrolytes (2 mg/mL 0.5 M NaCl). The alternating layers of negatively and positively charged polymers, i.e., PSS and PAH respectively were deposited around the charged sacrificial microparticle templates following a well established protocol [2]. Layer deposition was achieved by alternating immersion of microparticles inside the corresponding polymer solution (5 mL) for 13 min, followed by subsequent washing with DDW to remove excess polymers. Finally the cores of PEM capsules were dissolved by complexion of Ca^{2+} ions with EDTA (5 mL, 0.2 M, pH 6.5) and particles were washed with DDW and stored at 4 °C for further use. In order to minimize the artifacts due to size variation of capsules (mean diameter ≈ 3.5-4 µm), the cores were manufactured on large scale, dried under vacuum after washing with acetone, and stored at 4 °C. Latter in all experiments the same cores were used for LBL deposition of polyelectrolyte shells.

SNARF is a ratiometric pH sensitive dye having pK_a value of 7.5 [3]. After incorporation inside the cavity of capsules, the dye retained its pH sensitive fluorescence characteristics [1d]. Upon excitation at 543 nm it has two emission peaks at 580 nm (yellow fluorescence) and 640 nm (red fluorescence). When the ambient pH is low its emission maxima is at 580 nm, while at high pH the emission maximum is at 640 nm. This pH dependent shift in fluorescence intensity makes SNARF-bearing capsules ratiometric indicators for intracellular sensing without the need of additional reference fluorophores [1d, 4]. The pH dependent fluorescence response of the SNARF capsules as synthesized for the present work was monitored by adjusting the pH of the capsules by means of immersion in commercially available buffers (pH 3 - 10), cf. Figure SI-I.2.1 [1d, 5]. The intensity of red (640 nm) and yellow (580 nm, displayed as "green" in false colors of the microscope)) fluorescence of SNARF was measured using confocal laser scanning microscopy (CLSM) and the ratio of the respective fluorescence intensities was plotted versus pH.

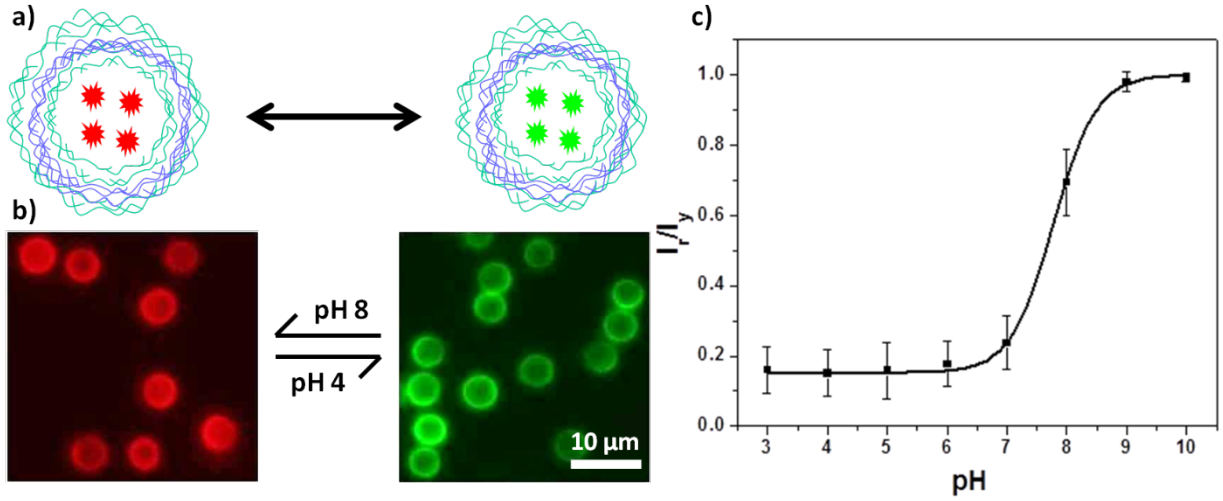

Figure SI-I.2.1: (a) Schematic diagram of a SNARF-1 dextran loaded PEM pH sensor capsule. (b) Fluorescence microscopy images of sensor capsules under acidic (yellow fluorescence, shown in green false colors) and alkaline (red fluorescence) conditions. (c) Dependence of the fluorescence signal of some batches (with 2 and 2.5 bilayers having positive and negative surface charge, respectively) of sensor capsules on ambient pH in terms of the ratio of red to yellow fluorescence intensity I_r/I_y at different pH values.

The hydrodynamic diameters and zeta potentials of SNARF-containing PEM capsules can be seen in Figure SI-I.2.2.

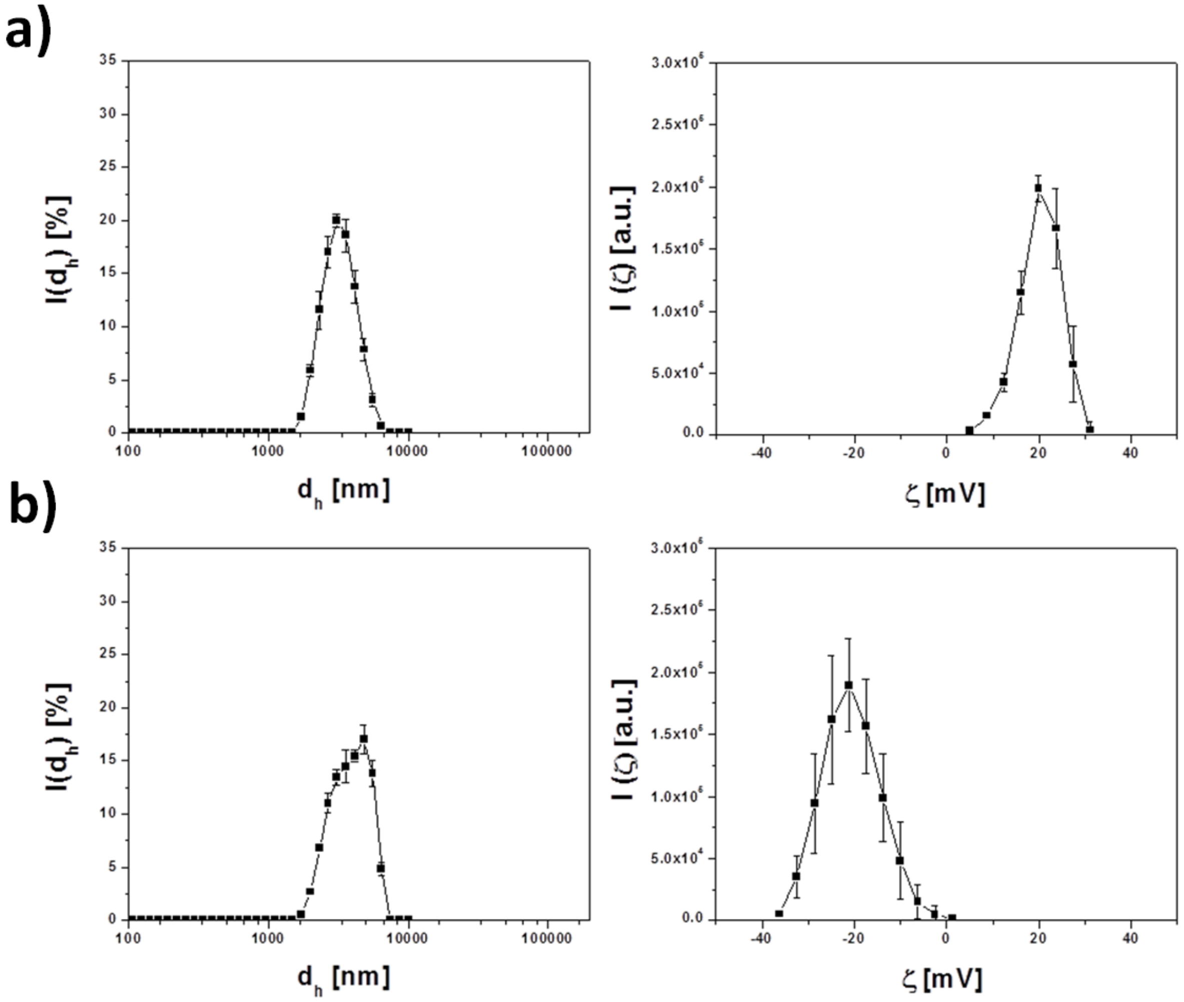

Figure SI-I.2.2: The hydrodynamic diameters d_h in terms of the intensity distributions $I(d_h)$ and zeta potential measurements of SNARF positive (a), and negative (b) capsules recorded in water. The results are presented as mean of 3 measurements ± standard deviations. The corresponding values are provided in the Table SI-I.2.1.

Capsule charge	d_h [µm]	Δd_h [µm]	ζ [mV]	Δζ [mV]
+	3.1	0.006	21.2	2.2
-	4.8	0.001	- 21.2	6.5

Table SI-I.2.1: Colloidal properties of the capsules. Hydrodynamic diameter $d_h \pm \Delta d_h$ and zeta potential $\zeta \pm \Delta\zeta$ as measured in water given as average and standard deviation. These data were derived from Figure SI-I.2.2.

I.3) Uptake studies by confocal laser scanning microscope (CLSM)

HeLa cells were grown in DMEM supplemented with 10% FBS, 1% P/S, and 1% Glutamax. 15,000 cells were seeded per well in 8-well µ-slides having growth area of 1 cm^2 per well. Each

well was filled with 300 μL of growth medium. Cells were kept inside an incubator set to 37 °C and complemented with 5% CO_2 at constant rate. Capsule tracking and uptake experiments were performed in serum supplemented or in serum deprived media, in the presence or absence of inhibitors using CLSM. In order to track the internalization of PEM sensor capsules, the CLSM was equipped with a portable incubator in order to maintain the μ-slides at 37 °C with 5% CO_2. Before starting the experiment the cells were provided fresh serum supplemented/ serum deprived media. In addition, the inhibitors (cytochalasin D, 300 nM & bafilomycin A1, 0.25 μM) were added 1 hour before the addition of PEMs and starting the experiments. PEMs were provided to the cells at a concentration of 5 capsules per seeded cell just at the time of start of the imaging using a Plan-Apochromat 63x/1.40 Oil DIC M27 objective. During imaging, time lapse image series were captured [1f]. In order to minimize photobleaching of SNARF during image acquisition, very low laser excitation power was used in order to detect fluorescence for long term studies. The fluorophore was excited at λ_{ex} = 543 nm and its fluorescence emission in the yellow and in the red region was detected using a 560 - 615 nm band pass filter and a 633 nm long pass filter, respectively. During imaging the lateral resolution (i.e. in the x-y-plane) was adjusted to 0.32 μm, whereas, 120 s temporal resolution was used. In order to acquire two images from the same lateral position, the z-position (i.e. the height of the focus with respect to the substrate) of the maximum scattered photons at the boundary of substrate/medium (μ-dish plate/cell medium) was detected, which helped in determining the absolute axial position of this boundary layer [1f]. Two imaging slices were acquired 3 and 2.2 μm above the substrate in order to resolve the extracellular (attached with the cells) and intracellular (internalized by the cells) capsules, respectively. For imaging of both slices the pinhole was adjusted to capture 2 μm thick image sections. A software based autofocus routine was always set to recover the imaging alignment before each time point of measurement [1f]. Image dimensions were further reduced after determining the projection along the z-axis of each slice at the end of image acquisition.

I.4) Particle tracking by CLSM, image processing, and data evaluation

The experimental protocol and description follows a previous study by Hartmann et al. [1f]. Large CLSM images were cut in small segments covering the whole internalization path of given capsules. A Matlab (Mathworks, USA) based particle analyzing toolbox written by Raimo Hartmann [1f] was used for visualization, identification, particle (capsules) segmentation, tracking, and automated image processing, as described previously [1f]. In this process, by means of median filtering photon shot noise was decreased in the red and yellow (green in false colors) channels of CLSM images and the background was entirely eliminated after defining a threshold. A modified Hough transformation was computed by using the method of Gonzalez, et al., for automatic identification, and masking the PEM capsules from CLSM images. This mask was later used for defining regions of interest (ROIs) around the regions of maximum fluorescence intensity [6]. The dimensions of PEM capsules resembled circles, that is why the centers of all circles were identified by checking the positions of individual pixels

whether they match the origin of a circle of given radius. From the classical Hough transformation the radius of the circles cannot be determined. That is why the parameterization of a donut shaped structure (shell thickness ≈ 0.64 µm) was used in order to identify the positions and dimensions of individual capsules. The radius of this structure was varied for each pixel and the sum of the underlying pixel intensities was recorded as a function of this radius. The capsule radius was determined from the first maximum of this function. Individual capsules were trace as described in Hartmann et al [1f]. A mask for each capsule's ROIs (in the red and yellow fluorescence channel) was created by using their respective x-y coordinates and radii. The average red/yellow ratio designated as I_r/I_y corresponding to the ambient pH values was calculated from the noise-corrected raw data within the masked regions. Taking inspiration by the particle tracking algorithms developed by John Crocker et al [7], which were provided by Danial Blair and Eric Dufresne for Matlab, a capsule tracking function was applied in order to get the progression of I_r/I_y versus time for individual capsules during their uptake and cellular trafficking (i.e., so called trajectories). A brief description of the workflow for the whole image processing is described in a previous study [1f].

Some representative examples of trajectories of SNARF capsules having different surface charge and bilayers in serum-supplemented and serum-deprived media and in the presence and absence of inhibitors are provided in Figure SI-I.4.2 - Figure SI-I.4.6, along with their corresponding CLSM images at certain specified time points. From the CLSM images the time t_C at which the PEM capsule first touches the cell was derived. In addition, from the position data also the capsule velocity v [µm/s] was obtained and was plotted versus time.

The trajectories of individual internalized capsules were used to determine their acidification (Δt_A) and processing (Δt_P; e.g., $\Delta t_{P10\%}$ and $\Delta t_{P50\%}$) times upon internalization [1f]. The acidification time Δt_A was determined from the sigmoidal curve as the time interval describing the duration of acidification, i.e. the duration of the transition from high to low pH. The processing time $\Delta t_{P50\%}$ is defined as the time interval from once a capsule attaches to the cell membrane until it becomes located in acidic endosomes/lysosomes [1f]. $\Delta t_{P10\%}$ is the time interval from the first contact of the PEM capsule with a cell until 10% of the final drop in pH have happened. The time after addition of the capsules to the cells until the first contact of the observed capsule with a cell is defined as time of first contact t_C. Examples are shown in Figure SI-I.4.2 - Figure SI-I.4.6. The following equation was used for fitting the curves in sigmoidal shape from the experimental data, see Figure SI-I.1.4.1 [1f]:

$$(I_r/I_y)(t) = (R_a-R_b)/(1+\exp((t-t_{50\%})/\Delta T))+R_b \qquad \text{(Equation SI-I.4.1)}$$

From each fit 4 fit parameters were obtained:

R_a = $(I_r/I_y)_{max}$, the I_r/I_y ratio when the capsules are still in the cell medium, i.e. at neutral/slightly alkaline pH

R_b = $(I_r/I_y)_{min}$, the I_r/I_y ratio when the capsules are fully internalized ,i.e. at maximum acidic pH in endo/lysosomal compartments

$t_{50\%}$, the time at which upon internalization the pH has dropped, so that 50% of the acidification has been achieved

ΔT, describing the length of the acidification period in which the pH drops

From these fit parameters the following set of parameters is obtained. $t_{10\%}$ and $t_{90\%}$ are the times when upon internalization 10% and 90% of the pH drop have been achieved:

$(I_r/I_y)(t_{10\%}) = (R_a-R_b)/(1+\exp((t_{10\%}-t_{50\%})/\Delta T))+R_b= (0.9\cdot(R_a-R_b) + R_b$

$\Rightarrow 1+\exp((t_{10\%}-t_{50\%})/\Delta T) = (R_a-R_b)/(0.9\cdot(R_a-R_b) = 10/9$

$\Rightarrow \exp((t_{10\%}-t_{50\%})/\Delta T) = 1/9$

$\Rightarrow (t_{10\%}-t_{50\%})/\Delta T = \ln(1/9)$

$\Rightarrow t_{10\%}-t_{50\%} = \ln(1/9)\cdot\Delta T$

In an analogous way one can calculate

$(I_r/I_y)(t_{90\%}) = (R_a-R_b)/(1+\exp((t_{90\%}-t_{50\%})/\Delta T))+R_b= 0.1\cdot(R_a-R_b) + R_b$

$\Rightarrow t_{90\%}-t_{50\%} = \ln(9)\cdot\Delta T$

This leads to the definition of the acidification time Δt_A, which is the time interval in which the pH drop goes from 10% to 90%:

$\Delta t_A = t_{90\%}-t_{10\%} = \ln(0.9)\cdot\Delta T - \ln(1/9)\cdot\Delta T = 2\cdot\ln(9)\cdot\Delta T$ (Equation SI-I.4.2)

Based on this also the processing time $\Delta t_{P10\%}$ and $\Delta t_{P50\%}$ are calculated:

$t_{10\%}= \ln(1/9)\cdot\Delta T + t_{50\%}$

$t_{90\%} = \ln(9)\cdot\Delta T + t_{50\%}$

$\Rightarrow$

$\Delta t_{P10\%} = t_{10\%} - t_C$ (Equation SI-I.4.3)

$\Delta t_{P50\%} = t_{50\%} - t_C$

These are the time intervals needed from the first contact of a capsule with a cell unto 10% and 90% of the pH drop in the locale capsule environment due to internalization has been achieved. $t_{50\%}$ in contrast refers to the start of incubation $t_{50\%} = t_C + \Delta t_{P50\%}$. It refers to the time needed from the start of incubation until 50% acidification has been achieved. In addition, the maximum of the absolute slope of the pH response was determined as $(|d(I_r/I_y)/dt|)_{max}$. Thus, from the fit of each position/pH trace the following 5 parameters were obtained: t_C, t_A, $t_{P10\%}$, $t_{P50\%}$, $(|d(I_r/I_y)/dt|)_{max}$. Some examples are given in the following.

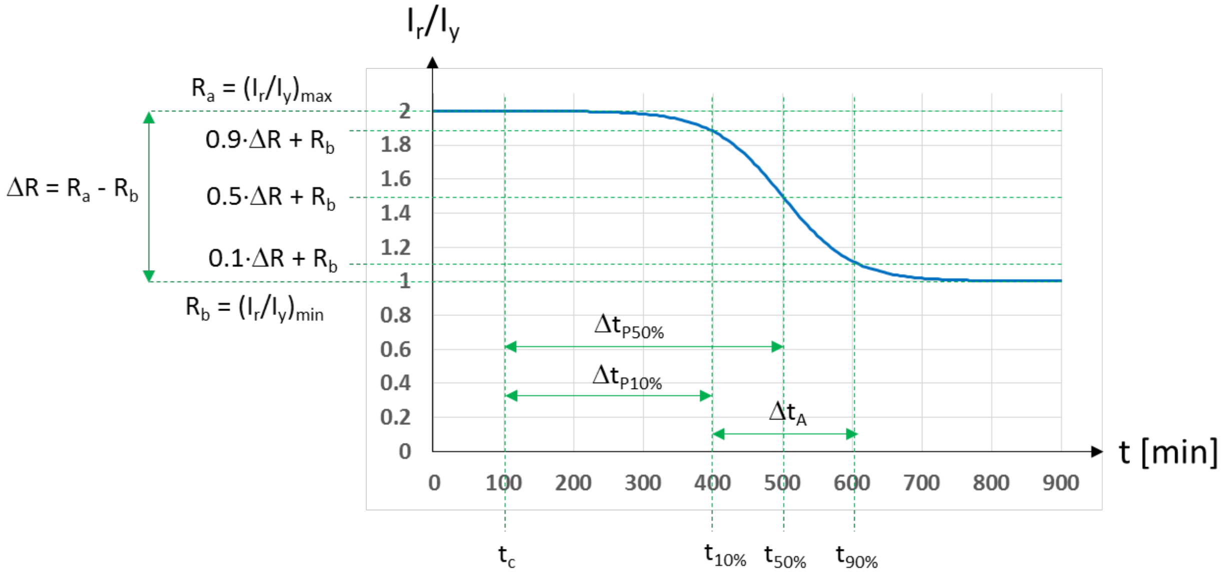

Figure SI-I.4.1: Temporal change of the intensity ratio I_r/I_y (i.e. the pH) of a SNARF-filled PEM capsule upon its internalization by a cell. Capsules are added to cells at time 0. At time t_c the observed capsules touches the cell. This cannot be seen in the I_r/I_y trace, but from the corresponding CLSM image.

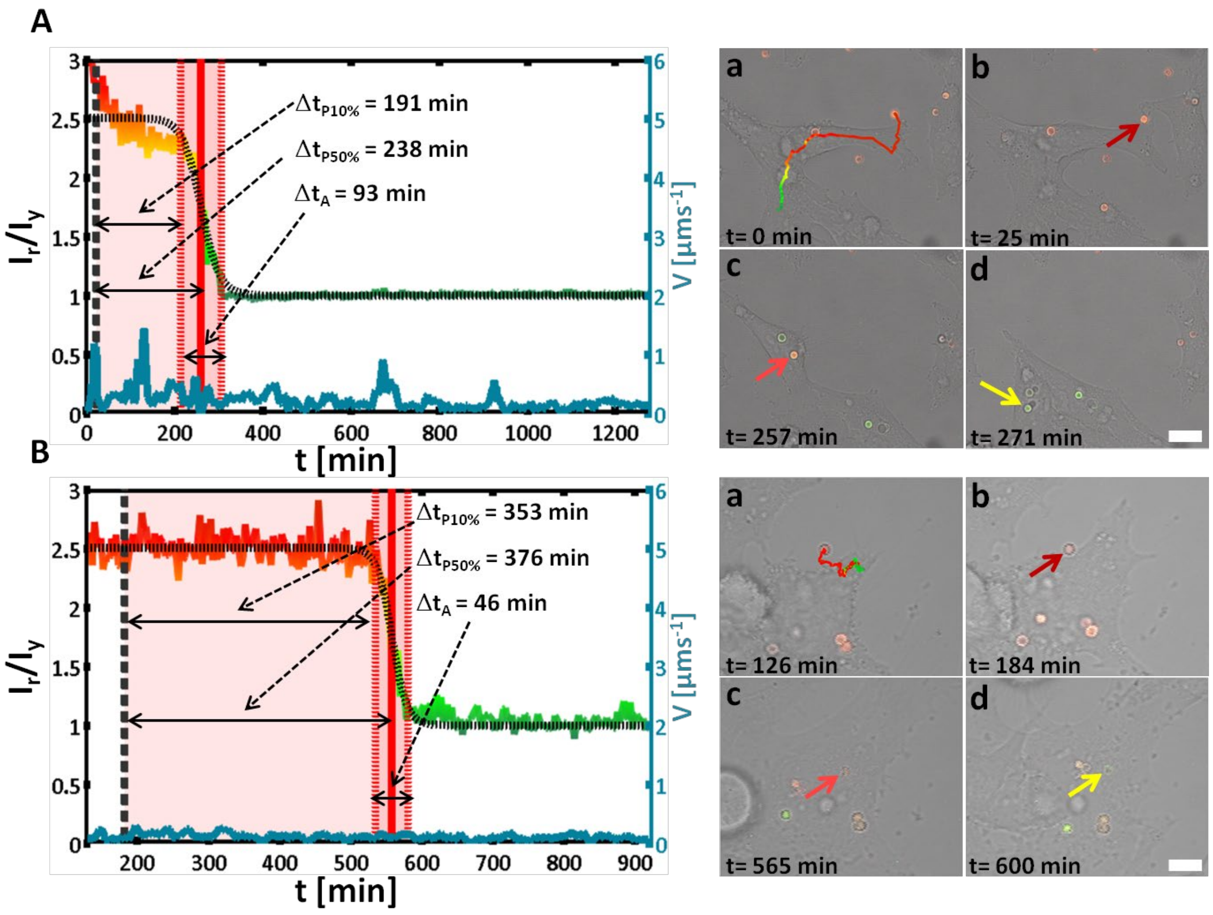

Figure SI-I.4.2: Representative trajectories of SNARF-loaded capsules (positively charged, 2 bilayers) in serum-supplemented culture A) without addition of cytochalasin D, and B) upon

the presence of 300 nM cytochalasin D during internalization by HeLa cells. On the right side representative corresponding CLSM images are provided, showing different time points of capsules internalization: a) in the proximity of cells, b) 1st contact with cells, c) start of conversion of local capsule environment from slightly alkaline to acidic environment, and d) fully acidified intra-lysosomal environment. The scale bars correspond to 10 µm. In the graphs also the capsule velocity v is plotted versus time.

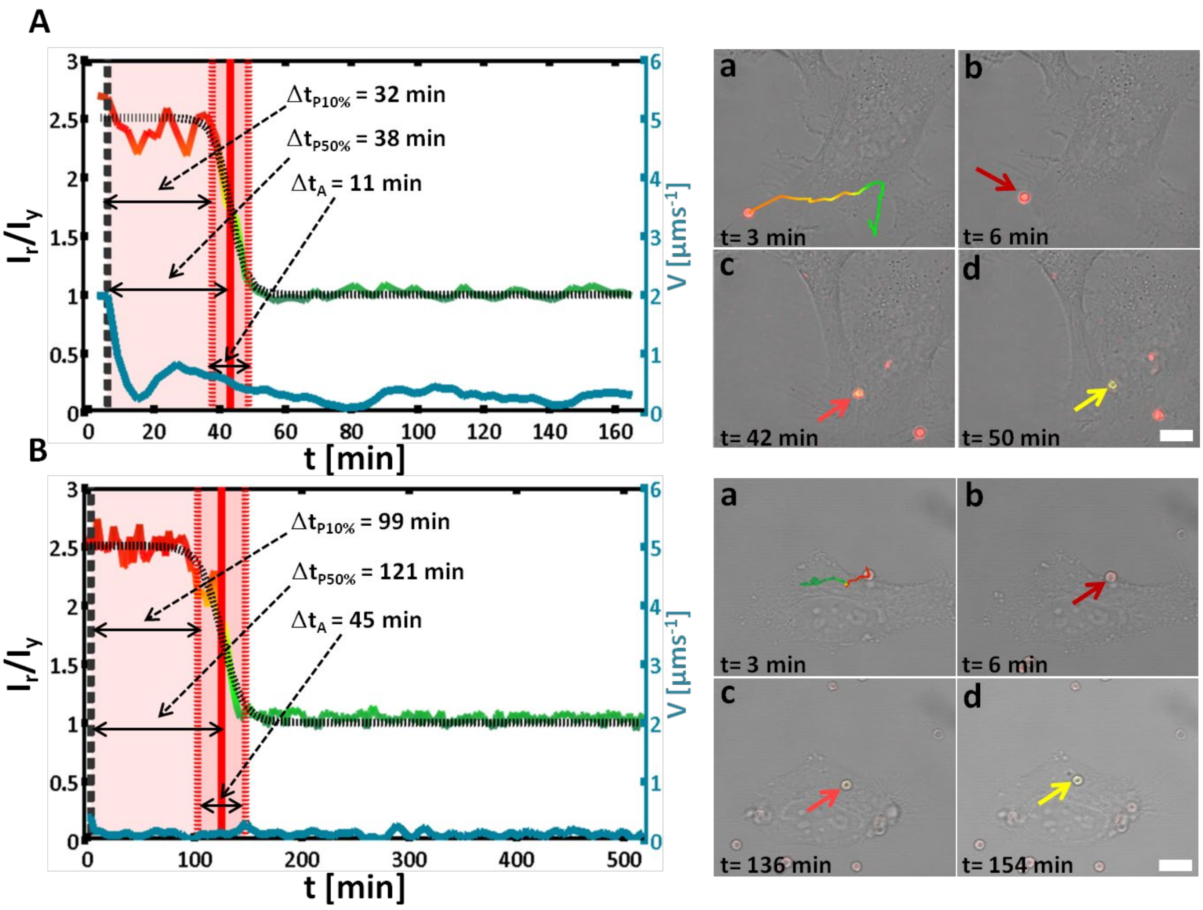

Figure SI-I.4.3: Representative trajectories of SNARF-loaded capsules (positively charged, 2 bilayers) in serum-deprived culture A) without addition of cytochalasin D, and B) upon the presence of 300 nM cytochalasin D during internalization by HeLa cells. On the right side representative corresponding CLSM images are provided, showing different time points of capsules internalization: a) in the proximity of cells, b) 1st contact with cells, c) start of conversion of local capsule environment from slightly alkaline to acidic environment, and d) fully acidified intra-lysosomal environment. The scale bars correspond to 10 µm.

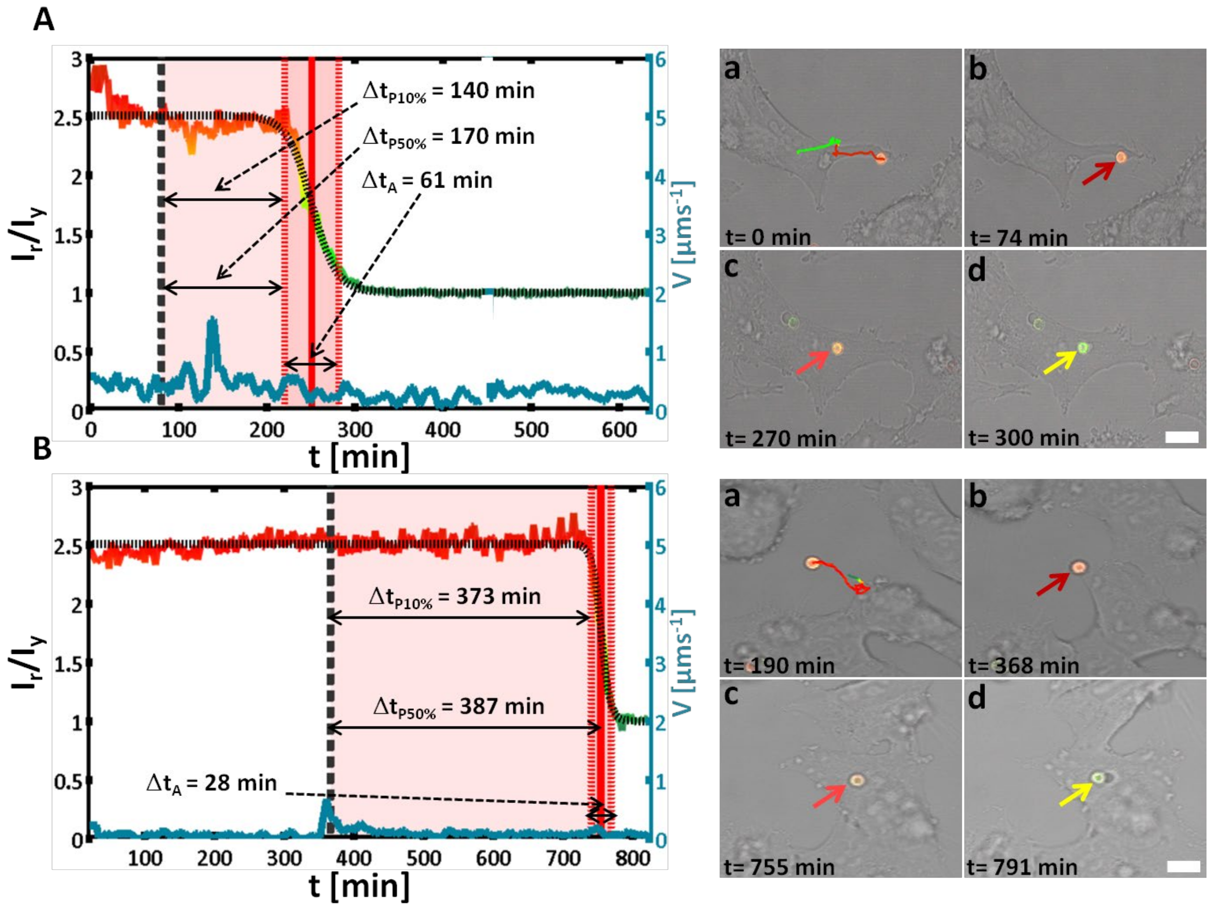

Figure SI-I.4.4: Representative trajectories of SNARF-loaded capsules (negatively charged, 2.5 bilayers) in serum-supplemented culture A) without addition of cytochalasin D, and B) upon the presence of 300 nM cytochalasin D during internalization by HeLa cells. On the right side representative corresponding CLSM images are provided, showing different time points of capsules internalization: a) in the proximity of cells, b) 1st contact with cells, c) start of conversion of local capsule environment from slightly alkaline to acidic environment, and d) fully acidified intra-lysosomal environment. The scale bars correspond to 10 µm.

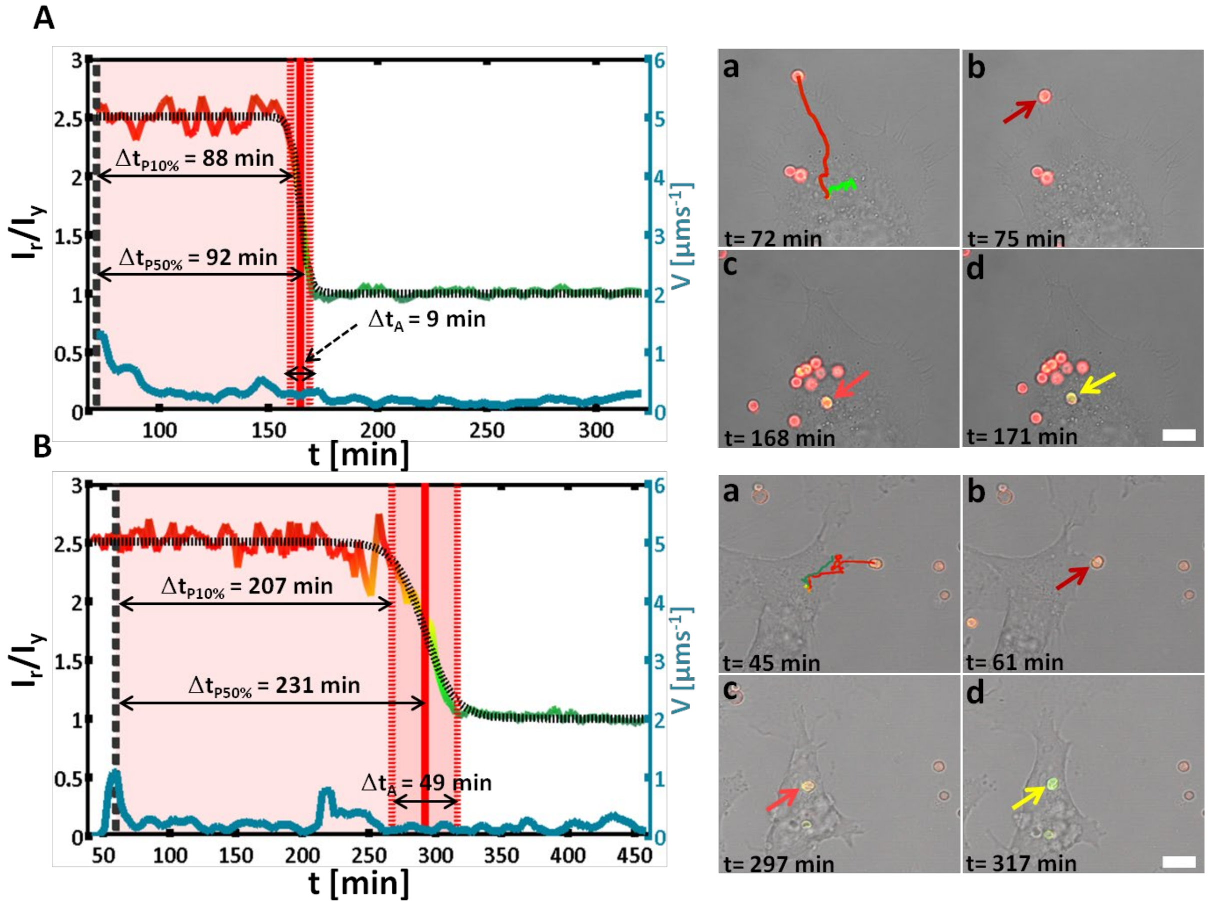

Figure SI-I.4.5: Representative trajectories of SNARF-loaded capsules (negatively charged, 2.5 bilayers) in serum-deprived culture A) without addition of cytochalasin D, and B) upon the presence of 300 nM cytochalasin D during internalization by HeLa cells. On the right side representative corresponding CLSM images are provided, showing different time points of capsules internalization: a) in the proximity of cells, b) 1st contact with cells, c) start of conversion of local capsule environment from slightly alkaline to acidic environment, and d) fully acidified intra-lysosomal environment. The scale bars correspond to 10 µm.

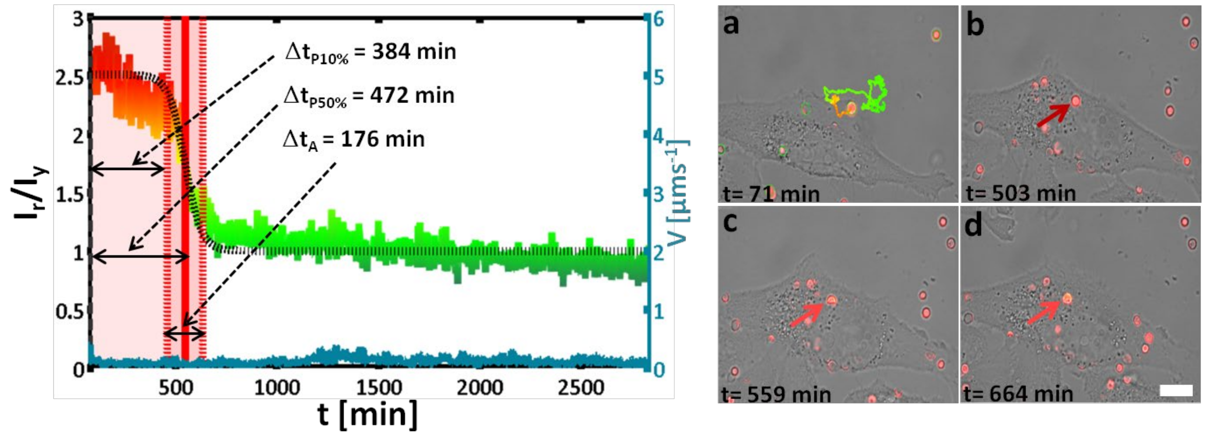

Figure SI-I.4.6: Representative trajectory of SNARF-loaded capsule (positively charged, 2 bilayers) in serum-supplemented culture in the the presence of 0.25 µM bafilomycin A1 during

internalization by HeLa cell. On the right side representative corresponding CLSM images are provided, showing different time points of capsule's internalization: a) in the proximity of cells, b) 1st contact with cells, c) start of conversion of local capsule environment from slightly alkaline to acidic environment, and d) fully acidified intra-lysosomal environment. The scale bars correspond to 10 µm.

Note, that the bafilomycin A1 is lysomotropic reagent which alkalinizes the intra-lysosomal pH and inhibits the internalization of PEM capsules [5, 8]. The experimental concentration of the reagent was selected that it (partly) inhibited capsule internalization, and the intra-lysosomal pH remained less than pH of the extracellular medium. That is the reason why the internalized capsules turn orange instead of yellow (i.e. less acidic environment than without the presence of bafilomycin A1). Moreover, it was hard to identify the point of inflection in the I_r/I_y traces, i.e. the parameter $t_{50\%}$. Thus, only a limited number of experiments were performed with this reagent and the parameters (e.g. acidification, processing time, etc.) are not provided in results.

I.5) Fractal dimension and the average end-to-end scaling exponent

Similarly other parameters, such as fractal dimensions D and the average end-to-end scaling exponent ν, can be determined from this data set. These data are based on the particle trajectories in the CLSM images. The position of each capsule at time t is given by its x- and y-coordinates x(t) and y(t). At images were taken in time intervals of 2 min, discrete coordinates $x_i = x(t_i)$ and $y_i = y(t_i)$ were obtained with $t_{i+1} - t_i = 2$ min.

To characterize the trajectories quantitatively one can make use of the fractal dimension D, which is a measure of self-similarity and, thus, remains unchanged when the scale of measurement is changed [9]. D is also a measure of spatial extent, i.e. the space filling properties, and self-affinity [9]. The correct determination of the fractal dimension of a trajectory is a nontrivial problem, several approaches exist in the literature [9-10]. Adopting the method by Sevcik et al. [9] the fractal dimensions D of the full capsule trajectories were approximated by the fractal dimensions D_M of each capsule trajectory consisting of M sample points. In doing so, the trajectory (x,y)(t) in the two-dimensional plane of the M sample points (each at time t_i, i = 1,…, M) was mapped into a unit square

$$x_i \rightarrow \frac{|x_i - x_{min}|}{|x_{max} - x_{min}|}$$

$$y_i \rightarrow \frac{|y_i - y_{min}|}{|y_{max} - y_{min}|} \qquad \text{(Equation SI-I.5.1)}$$

Where, $x/y_{max/min}$ is the maximum/minimum of x/y_i. Then, the fractal dimension D of the trajectory was approximated by

$$D \approx D_M = 1 + \frac{\log L}{\log(2M-2)} \qquad \text{(Equation SI-I.5.2)}$$

Where, L is the contour length of the trajectory in the unit square:

$$L = \sum_{i=1}^{M} \Delta L_i = \sum_{i=1}^{M} \sqrt{\Delta x_i^2 + \Delta y_i^2} \qquad \text{(Equation SI-I.5.3)}$$

with $\Delta x_i = |x_{i+1} - x_i|$ etc.

The average end-to-end distance $\langle R(L) \rangle$ is a strong characteristic of the spatial structure of polymers [11]. Given a polymer of contour length L, the average end-to-end distance <R(L)> scales as

$$\langle R(L) \rangle \propto L^{\nu} \qquad \text{(Equation SI-I.5.4)}$$

The exponent ν depends on the dimension of the system, taking values from ν = 1 to ν = 0. Similar to this scaling behavior of polymers one can use the average end-to-end distance scaling exponent to characterize the trajectory of a capsule. The scaling exponent ν is then given by

$$\nu = \frac{\log\langle R(L) \rangle}{\log L} \qquad \text{(Equation SI-I.5.5)}$$

with $\langle R(L) \rangle = \sqrt{(x_M - x_0)^2 + (y_M - y_0)^2}$ and $L = \sum_{i=1}^{M} (\Delta x_i^2 + \Delta y_i^2)^{1/2}$, where, x_M and y_M are the last sample points of the trajectories of each capsule along the x- and y- dimension. Note, that for the scaling exponent the trajectories were not mapped into a unit square, but the absolute values were used.

I.6) Results

The results from uptake data of capsules of almost similar sizes in terms of various surface charge, presence and absence of serum, and cytochalasin D inhibitor by determining various parameters from the trajectories of internalized particles are presented in the following. Each data set corresponds to at least 100 different trajectories that were evaluated, the exact number is given in Table SI-I.6.1. Two independent complete set of experiments with two batches of SNARF-loaded capsules were performed in order to validate the effect of cytochalasin D (300 nM, i.e. a potent inhibitor of particle internalization by disrupting actin polymerization [8]) on the uptake and intracellular processing of these capsules. Results are listed individually for both batches. The extracted parameters t_C, Δt_A, $\Delta t_{P50\%}$, $\Delta t_{P10\%}$, $t_{P50\%}$, and $(|d(I_r/I_y)/dt|)_{max}$ are enlisted in Table SI-I.6.2 - Table SI-I.6.7 and displayed in Figure SI-I.6.1.

capsule charge	serum	cytochalasin D	n
+	w	w/o	144
+	w	w^1	99
+	w	w^2	220
+	w/o	w/o	222
+	w/o	w^1	141
+	w/o	w^2	87
-	w	w/o	120
-	w	w^1	125
-	w	w^2	106
-	w/o	w/o	398
-	w/o	w^1	121
-	w/o	w^2	105

Table SI-I.6.1. Number of traces n that were evaluated for SNARF-loaded capsules added to HeLa cells with positive ("+", 2 bilayers) and negative ("-", 2.5 bilayers) charge, with serum-supplemented ("w") and serum deprived ("w/o") conditions, with ("w": two independent experiments "w^1" and "w^2") and without ("w/o") the presence of 300 nM cytochalasin D, as determined from the trajectories of capsules while cellular uptake.

capsule charge	serum	cytochalasin D	t_C [min]	Δt_C [min]
+	w	w/o	92	35
+	w	w^1	84	19
+	w	w^2	54	12
+	w/o	w/o	49	7
+	w/o	w^1	54	11
+	w/o	w^2	65	18
-	w	w/o	19	12
-	w	w^1	84	16
-	w	w^2	98	32
-	w/o	w/o	24	3
-	w/o	w^1	60	13
-	w/o	w^2	42	12

Table SI-I.6.2. Contact time t_C of SNARF-loaded capsules added to HeLa cells with positive ("+", 2 bilayers) and negative ("-", 2.5 bilayers) charge, with serum-supplemented ("w") and serum deprived ("w/o") conditions, with ("w": two independent experiments "w^1" and "w^2") and without ("w/o") the presence of 300 nM cytochalasin D, as determined from the trajectories

of capsules while cellular uptake. The data for the contact time is provided as median values t_C, plus minus the confidence intervals Δt_C.

capsule charge	serum	cytochalasin D	Δt_A [min]	$\Delta\Delta t_A$ [min]
+	w	w/o	45	6
+	w	w^1	56	8
+	w	w^2	66	9
+	w/o	w/o	25	2
+	w/o	w^1	44	9
+	w/o	w^2	61	22
-	w	w/o	43	7
-	w	w^1	35	4
-	w	w^2	37	4
-	w/o	w/o	16	1
-	w/o	w^1	22	2
-	w/o	w^2	38	5

Table SI-I.6.3. Acidification time Δt_A of SNARF-loaded capsules added to HeLa cells with positive ("+", 2 bilayers) and negative ("-", 2.5 bilayers) charge, with serum-supplemented ("w") and serum deprived ("w/o") conditions, with ("w": two independent experiments "w^1" and "w^2") and without ("w/o") the presence of 300 nM cytochalasin D, as determined from the trajectories of capsules while cellular uptake. The data for the acidification time is provided as median values Δt_A, plus minus the confidence intervals $\Delta\Delta t_A$.

capsule charge	serum	cytochalasin D	$\Delta t_{P50\%}$ [min]	$\Delta\Delta t_{P50\%}$ [min]
+	w	w/o	257	35
+	w	w^1	249	27
+	w	w^2	194	18
+	w/o	w/o	110	12
+	w/o	w^1	137	12
+	w/o	w^2	207	24
-	w	w/o	305	61
-	w	w^1	253	25
-	w	w^2	296	42
-	w/o	w/o	74	7
-	w/o	w^1	144	17
-	w/o	w^2	276	25

Table SI-I.6.4. Processing time $\Delta t_{P50\%}$ of SNARF-loaded capsules added to HeLa cells with positive ("+", 2 bilayers) and negative ("-", 2.5 bilayers) charge, with serum-supplemented ("w") and serum deprived ("w/o") conditions, with ("w": two independent experiments "w^1" and "w^2") and without ("w/o") the presence of 300 nM cytochalasin D, as determined from the trajectories of capsules while cellular uptake. The data for the 50% processing time is provided as median values $\Delta t_{P50\%}$, plus minus the confidence intervals $\Delta\Delta t_{P50\%}$.

capsule charge	serum	cytochalasin D	$\Delta t_{P10\%}$ [min]	$\Delta\Delta t_{P10\%}$ [min]
+	w	w/o	234	37
+	w	w^1	216	28
+	w	w^2	147	17
+	w/o	w/o	95	12
+	w/o	w^1	93	13
+	w/o	w^2	137	20
-	w	w/o	287	60
-	w	w^1	222	26
-	w	w^2	268	42
-	w/o	w/o	64	7
-	w/o	w^1	130	15
-	w/o	w^2	249	25

Table SI-I.6.5. Processing time $\Delta t_{P10\%}$ of SNARF-loaded capsules added to HeLa cells with positive ("+", 2 bilayers) and negative ("-", 2.5 bilayers) charge, with serum-supplemented ("w") and serum deprived ("w/o") conditions, with ("w": two independent experiments "w^1" and "w^2") and without ("w/o") the presence of 300 nM cytochalasin D, as determined from the trajectories of capsules while cellular uptake. The data for the 10% processing time is provided as median values $\Delta t_{P10\%}$, plus minus the confidence intervals $\Delta\Delta t_{P10\%}$.

capsule charge	serum	cytochalasin D	$t_{50\%}$ [min]	$\Delta t_{50\%}$ [min]
+	w	w/o	349	35
+	w	w^1	333	23
+	w	w^2	248	15
+	w/o	w/o	159	10
+	w/o	w^1	191	12
+	w/o	w^2	272	21
-	w	w/o	324	37
-	w	w^1	337	21

-	w	w^2	394	37
-	w/o	w/o	98	5
-	w/o	w^1	204	15
-	w/o	w^2	318	19

Table SI-I.6.6. Time $t_{50\%}$ until 50% acidification of SNARF-loaded capsules added to HeLa cells with positive ("+", 2 bilayers) and negative ("-", 2.5 bilayers) charge, with serum-supplemented ("w") and serum deprived ("w/o") conditions, with ("w": two independent experiments "w^1" and "w^2") and without ("w/o") the presence of 300 nM cytochalasin D. The data is provided as median values $t_{50\%}$, plus minus the confidence intervals $\Delta t_{50\%}$.

**capsule charge**	**serum**	**cytochalasin D**	**$(\vert d(I_r/I_y)/dt\vert)_{max}$ [min^{-1}]**	**$\Delta(\vert d(I_r/I_y)/dt\vert)_{max}$ [min^{-1}]**
+	w	w/o	0.06	0.007
+	w	w^1	0.03	0.004
+	w	w^2	0.035	0.003
+	w/o	w/o	0.095	0.008
+	w/o	w^1	0.034	0.006
+	w/o	w^2	0.036	0.006
-	w	w/o	0.09	0.012
-	w	w^1	0.063	0.006
-	w	w^2	0.06	0.006
-	w/o	w/o	0.24	0.014
-	w/o	w^1	0.08	0.009
-	w/o	w^2	0.062	0.008

Table SI-I.6.7. Maximum of the absolute first derivative of the I_r/I_y versus t traces of SNARF-loaded capsules added to HeLa cells with positive ("+", 2 bilayers) and negative ("-", 2.5 bilayers) charge, with serum-supplemented ("w") and serum deprived ("w/o") conditions, with ("w": two independent experiments "w^1" and "w^2") and without ("w/o") the presence of 300 nM cytochalasin D, as determined from the trajectories of capsules while cellular uptake. The data is provided as median values $(|d(I_r/I_y)/dt|)_{max}$, plus minus the confidence intervals, i.e., $\Delta(|d(I_r/I_y)/dt|)_{max}$.

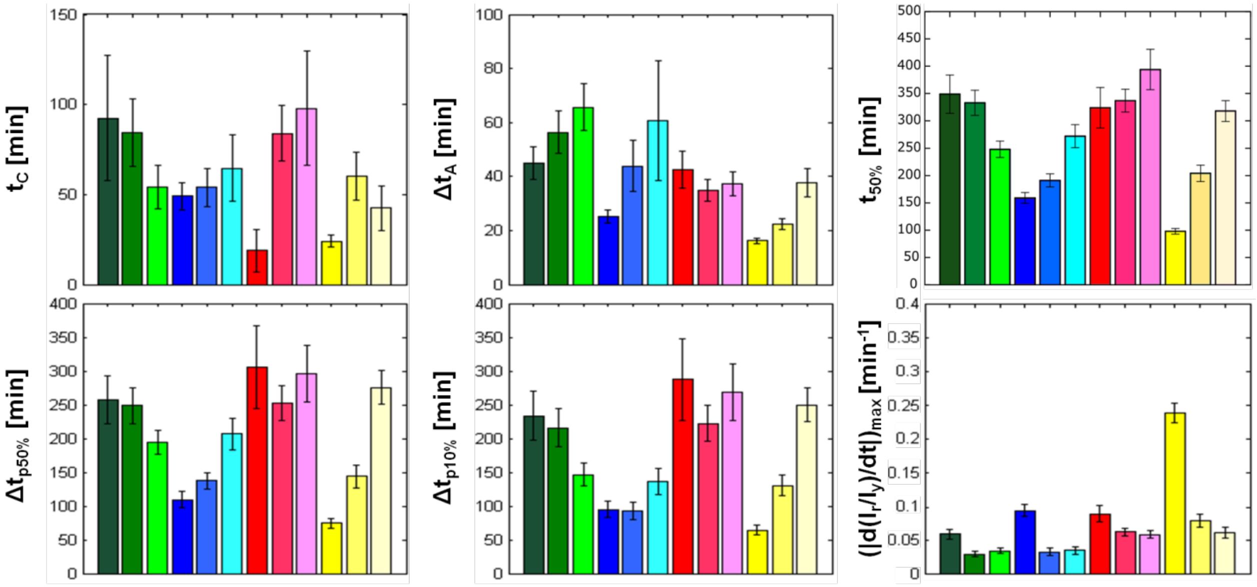

capsule charge	serum	cytochalasin D
+	w	w/o
+	w	w[1]
+	w	w[2]
+	w/o	w/o
+	w/o	w[1]
+	w/o	w[2]
-	w	w/o
-	w	w[1]
-	w	w[2]
-	w/o	w/o
-	w/o	w[1]
-	w/o	w[2]

Figure SI-I.6.1: Representation of various parameters of capsule tracking experiments derived from the trajectories of internalized capsules (after addition of 5 capsules per seeded cell), as taken from Table SI-I.6.2 - Table SI-I.6.7. Results are presented as median values plus/minus confidence intervals.

The fractal dimensions D and end-to-end distance scaling exponents ν were calculated for each trajectory for three different regions of the trajectory: before acidification starts (i.e. capsules are still outside cells "out"), during acidification ("uptake"), and after acidification (i.e. when the capsules are fully internalized "in"). Values are provided in Table SI-I.6.8 and Table SI-I.6.9 and the corresponding graphs in Figure SI-I.6.2 and Figure SI-I.6.3.

capsule charge	serum	cytochalasin D	D_{out}	D_{uptake}	D_{in}
+	w	w/o	1.31 ± 0.006	1.29 ± 0.007	1.307 ± 0.006
+	w	w[1]	1.297 ± 0.009	1.352 ± 0.009	1.34 ± 0.008
+	w	w[2]	1.29 ± 0.006	1.33 ± 0.006	1.34 ± 0.006
+	w/o	w/o	1.32 ± 0.009	1.38 ± 0.01	1.38 ± 0.006

+	w/o	w[1]	1.31 ± 0.012	1.333 ± 0.009	1.38 ± 0.009
+	w/o	w[2]	1.35 ± 0.014	1.37 ± 0.01	1.37 ± 0.01
-	w	w/o	1.34 ± 0.008	1.32 ± 0.01	1.346 ± 0.009
-	w	w[1]	1.296 ± 0.007	1.315 ± 0.01	1.32 ± 0.0065
-	w	w[2]	1.311 ± 0.009	1.32 ± 0.01	1.32 ± 0.009
-	w/o	w/o	1.322 ± 0.006	1.32 ± 0.01	1.344 ± 0.003
-	w/o	w[1]	1.29 ± 0.007	1.305 ± 0.01	1.32 ± 0.008
-	w/o	w[2]	1.322 ± 0.007	1.33 ± 0.01	1.34 ± 0.009

Table SI-I.6.8. Fractal dimensions D of the trajectories of SNARF-loaded capsules added to HeLa cells with positive ("+", 2 bilayers) and negative ("-", 2.5 bilayers) charge, with serum-supplemented ("w") and serum deprived ("w/o") conditions, with ("w": two independent experiments "w[1]" and "w[2]") and without ("w/o") the presence of 300 nM cytochalasin D. Trajectories were subdivided in the parts before acidification (D_{out}), during acidification (D_{uptake}) and after acidification (D_{in}). Results are presented as mean values ± standard deviations.

**capsule charge**	**serum**	**cytochalasin D**	ν_{out}	ν_{uptake}	ν_{in}
+	w	w/o	0.645 ± 0.01	0.678 ± 0.02	0.64 ± 0.02
+	w	w[1]	0.643 ± 0.021	0.506 ± 0.02	0.484 ± 0.02
+	w	w[2]	0.66 ± 0.016	0.512 ± 0.02	0.507 ± 0.02
+	w/o	w/o	0.66 ± 0.017	0.61 ± 0.02	0.51 ± 0.013
+	w/o	w[1]	0.63 ± 0.027	0.553 ± 0.026	0.46 ± 0.027
+	w/o	w[2]	0.57 ± 0.03	0.45 ± 0.03	0.445 ± 0.03
-	w	w/o	0.605 ± 0.01	0.64 ± 0.02	0.6 ± 0.014
-	w	w[1]	0.635 ± 0.019	0.54 ± 0.04	0.541 ± 0.019
-	w	w[2]	0.6 ± 0.02	0.55 ± 0.03	0.56 ± 0.026
-	w/o	w/o	0.63 ± 0.01	0.65 ± 0.04	0.551 ± 0.009
-	w/o	w[1]	0.65 ± 0.02	0.59 ± 0.05	0.556 ± 0.024
-	w/o	w[2]	0.58 ± 0.02	0.47 ± 0.04	0.49 ± 0.03

Table SI-I.6.9. Average end-to-end distance scaling exponents ν of the trajectories of SNARF-loaded capsules added to HeLa cells with positive ("+", 2 bilayers) and negative ("-", 2.5 bilayers) charge, with serum-supplemented ("w") and serum deprived ("w/o") conditions, with ("w": two independent experiments "w[1]" and "w[2]") and without ("w/o") the presence of 300 nM cytochalasin D. Trajectories were subdivided in the parts before acidification (ν_{out}), during acidification (ν_{uptake}) and after acidification (ν_{in}). Results are presented as mean values ± standard deviations.

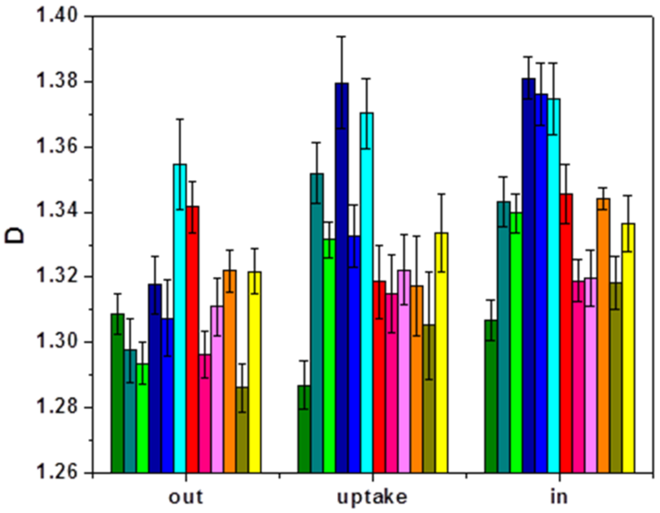

capsule charge	serum	cytochalasin D
+	w	w/o
+	w	w^1
+	w	w^2
+	w/o	w/o
+	w/o	w^1
+	w/o	w^2
-	w	w/o
-	w	w^1
-	w	w^2
-	w/o	w/o
-	w/o	w^1
-	w/o	w^2

Figure SI-I.6.2: Representation of fractal dimensions D of the trajectories from the capsule tracking experiments, as taken from Table SI-I.6.8. Results are presented as mean values ± standard deviations.

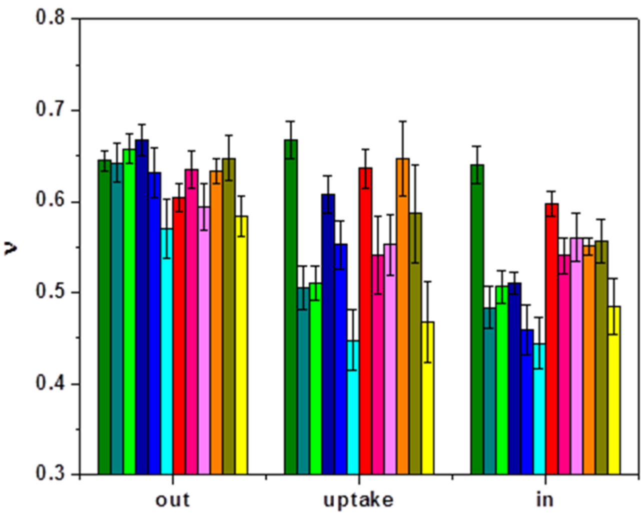

capsule charge	serum	cytochalasin D
+	w	w/o
+	w	w^1
+	w	w^2
+	w/o	w/o
+	w/o	w^1
+	w/o	w^2
-	w	w/o
-	w	w^1
-	w	w^2
-	w/o	w/o
-	w/o	w^1
-	w/o	w^2

Figure SI-I.6.3 Representation of average end-to-end distance scaling exponents ν of the trajectories from the capsule tracking experiments, as taken from Table SI-I.6.9. Results are presented as mean values ± standard deviations.

In the following possible differences in D and ν before, during, and after acidification are discussed, see Table SI-I.6.10.

capsule charge	serum	cytochalasin D	discussion about differences in D and ν
+	w	w/o	D_{out}, D_{in} identical, small difference to D_{uptake}<br>ν equal in all 3 phases
+	w	w^1	D_{uptake}, D_{in} identical, difference to D_{out}<br>ν_{uptake}, ν_{in} identical, difference to ν_{out}
+	w	w^2	D_{uptake}, D_{in} identical, difference to D_{out}<br>ν_{uptake}, ν_{in} identical, difference to ν_{out}
+	w/o	w/o	D_{uptake}, D_{in} identical, difference to D_{out}<br>ν all different
+	w/o	w^1	D increasing from D_{out} towards D_{uptake} to D_{in}<br>ν decreasing from ν_{out} towards ν_{uptake} to ν_{in}
+	w/o	w^2	D_{uptake}, D_{in} identical, difference to D_{out} (not significant)<br>ν_{uptake}, ν_{in} identical, difference to ν_{out}
-	w	w/o	D_{out}, D_{in} identical, difference to D_{uptake} (maybe not significant)<br>ν equal in all 3 phases
-	w	w^1	D_{uptake}, D_{in} identical, difference to D_{out} (maybe not significant)<br>ν_{uptake}, ν_{in} identical, difference to ν_{out}
-	w	w^2	D_{uptake}, D_{in} identical, difference to D_{out} (maybe not significant)<br>ν_{uptake}, ν_{in} identical, difference to ν_{out} (maybe not significant)
-	w/o	w/o	D_{out}, D_{uptake} identical, difference to D_{in}<br>ν_{out}, ν_{uptake} identical, difference to ν_{in}
-	w/o	w^1	D increasing from D_{out} towards D_{uptake} to D_{in} (large error in D_{uptake})<br>ν decreasing from ν_{out} towards ν_{uptake} to ν_{in} (large error in ν_{uptake})
-	w/o	w^2	D increasing from D_{out} towards D_{uptake} to D_{in} (maybe not significant)<br>ν decreasing from ν_{out} towards ν_{uptake} to ν_{in}

Table SI-I.6.10. Discussion about D and ν in the different regions before, during, and after acidification, of the trajectories.

An important question is what a statistical relevant difference in the measurement parameters is. The (average of the) fractal dimensions D differs for all data only between ~1.28 and 1.38, i.e. a difference far below 10%, which is not a very robust statement to distinguish between the different phases (i.e. before, during, and after acidification).

However, the scaling exponent ν differs between ~0.67 and 0.45, which is more significant, because it corresponds to a difference of almost 50%. Thus, if one takes into account the

correlation between the fractal dimension D and the scaling exponent ν (in general one would expect that when D $\rightarrow$ 1 (straight line) then also v $\rightarrow$ 1 and vice versa, which is in general true for the experimental data), then one might be able to extract some information about the different phases and between the different experiments using the data analysis. In that case, one might be able to make the statement that in some cases the existence of the different phases can be predicted from D and ν. It is however open to discussion whether differences are statistically significant. Considering the fractal dimension without the scaling exponent seems to result in rather non significant statements. However, taking both parameters into account may provide a chance to distinguish between the different phases before, during, and after acidification.

II) Uptake studies of SNARF-loaded capsules by cells using fluorescence microscopy

II.1) Materials, reagents, and equipment
II.2) Synthesis and characterization of SNARF-loaded polyelectrolyte micro- (PEM) capsules
II.3) Uptake studies based on capsule counting
II.4) Results

II.1) Materials, reagents, and equipment

Paraformaldehyde (8%; #157-8-100) was purchased from Electron Microscopy Sciences. Saponin (#S7900) and Hank's balanced salt solution (HBSS, #H8264) were purchased from Sigma-Aldrich. Glycin (#3908.1) was obtained from Roth. Bovine serum albumin (BSA; #001-000-161) and DyLight 649 donkey anti-mouse IgG (H+L, secondary antibody; #715-605-150) were purchased from Jackson ImmunoResearch Laboratories. Lysosomal-associated membrane protein 1 (LAMP 1; mouse anti-human IgG1; developmental studies hybridoma bank #H4A3, supernatant) was obtained from the University of Iowa, Department of Biology, USA. Phalloidin, oregon green 488 (# O7466) and tetramethylrhodamine dextran (TRITC dextran, $M_w \approx 70$ kDa, # D1818) were purchased from Thermo Fisher Scientific. Hoechst 33342, trihydrochloride, trihydrate (#H1399) was purchased from Life technologies. Fluoromount G was obtained from Southernbiotech (#0100-01). 24-well polystyrene cell culture plates (#83.3922.300) were purchased from SARSTEDT (Germany). Sterilized round cover slips (10 mm diameter, 0.17 ± 0.005 mm thickness, # YX02.1), glass slides (26 × 76 mm, #2299.2), and parafilm (#H951.1) were purchased from Carl Roth (Germany). All other chemicals/reagents for PEM capsules fabrication, cell culture, and microscopic studies were same as described in section §I.1.

An Axiovert 200M fluorescence microscope from Zeiss was used for capturing the images of SNARF-loaded capsules during their uptake by HeLa cells. Its tripod was equipped with a mercury arc lamp (N HBO 103) and a CCD camera (Axiocham HRc). The Axiovision 4.8 software was used to generate and process the fluorescence images. The microscope was equipped with an incubator (XL) in order to maintain a constant temperature (TempControl 37-2 digital) of 37 °C, humidity, and carbon dioxide supply (5%) to living cells. A 63x/1.40 (oil DIC ∞/ 0.17) oil immersion objective was used for observing capsules during intracellular trafficking. Filter sets were from AHF analysentechnik. In order to detect the fluorescence of SNARF, a D515/30 excitation filter was used (515 nm, 30 nm bandwidth), while the emission (of yellow and red fluorescence) was observed at 580 nm and 640 nm by D580/25, and D640/25 emission filters respectively. A 535 nm DCLP beam splitter was used.

For visualization the uptake of TRITC-loaded capsules by HeLa cells (immunostained samples) a CLSM 510 Meta from Zeiss was used. For sample visualization and image acquisition the CLSM was equipped with diode, argon, and helium neon lasers and the fluorophores were

excited at 405, 488, 543 and 633 nm respectively. Samples were observed through a 63X/1.40 oil-immersion DIC M27 objective.

II.2) Synthesis and characterization of SNARF-loaded polyelectrolyte micro- (PEM) capsules

The protocol for capsule fabrication is same as described in section §I.2. In addition to SNARF-modified dextran, TRITC-modified dextran was also used as fluorophore, due to the much lower price as compared to SNARF. TRITC-loaded capsules were prepared following the same methodology, by using TRITC-dextran instead of SNARF-dextran at the same concentration. The hydrodynamic diameter and zeta potential measurement results of TRITC-loaded PEM capsules in water are provided in Figure SI-II.2.1.

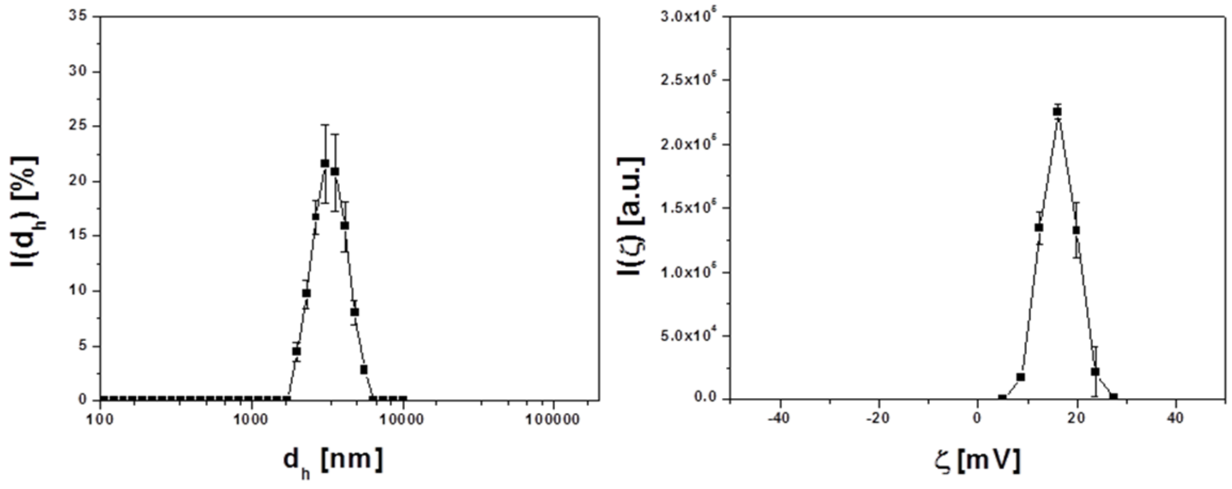

Figure SI-II.2.1: The hydrodynamic diameter d_h in terms of the intensity distributions $I(d_h)$ and zeta potential measurements of TRITC-loaded positively charged capsules as recorded in water. The results are presented as mean of 3 measurements ± standard deviations. The corresponding values are provided in the Table SI-II.2.1.

Capsule charge	d_h [µm]	Δd_h [µm]	ζ [mV]	$\Delta\zeta$ [mV]
+	3.1	0.003	16.2	0.7

Table SI-II.2.1: Colloidal properties of the capsules. Hydrodynamic diameter $d_h \pm \Delta d_h$ and zeta potential $\zeta \pm \Delta\zeta$ as measured in water given as average and standard deviation. These data were derived from Figure SI-II.2.1.

For SNARF-loaded capsules the uptake study was carried out at various time points. HeLa cells were seeded into 8 well µ-ibidi plates (surface area 1 cm^2/well) in 0.3 mL of complete growth medium at a density of 20,000 cells per well. After 18 h the growth media was exchanged to fresh growth media. Capsules were then added to cells. Uptake experiments were performed with two types of growth media, either supplemented with 10% FBS or without serum.

Positively and negatively charged capsules were added with two different concentrations, (i) at 10 capsules added per seeded cell, and (ii) at 20 capsules added per seeded cell. Cells were incubated inside an incubator at 37 °C with 5% CO_2 supply. Fluorescence microscopic images were recorded after 2, 4, 6, 12, 24, 30, 36, 42, and 48 hours of incubation with the help of an Axiovert fluorescence microscope using a 63x oil immersion objective. The fluorescence of SNARF was excited at 540 nm and its emission was recorded at 580 nm and 640 nm to detect the yellow and red fluorescence, respectively. Per sample and time point 10 images were captured, covering an average of 80 - 100 cells per condition. Experiments were performed in duplicate. A representative fluorescence microscope image of HeLa cells after 2 h incubation with SNARF-loaded capsules is shown in Figure SI-II.2.2a. Internalized capsules could be identified by their yellow fluorescence, whereas capsules remaining outside cells were fluorescent in red [12].

In order to evaluate the internalization of TRITC-loaded capsules, HeLa cells were seeded on small cover slips placed inside 24-well plates (surface area 1.82 cm^2 /well) in 0.6 mL complete growth medium at a density of 20,000 cells per well. After 22 h when the cells were firmly attached to the cover slips the growth media was aspirated and fresh growth media containing TRITC-loaded capsules was added. Uptake experiments were performed with two types of growth media, either supplemented with 10% FBS or without serum. Cells were incubated with positively and negatively charged capsules at a concentration of 10 capsules added per seeded cell and were incubated for 24 h inside an incubator at 37 °C with 5% CO_2 supply. In a series of experiments Bafilomycin A1 (0.25 µM) was added in order to investigate its effect as inhibitor on capsule uptake [5]. Bafilomycin A1 was added to cells with fresh growth media 1 h before the addition of capsules. The capsules were subsequently immunostained (cellular cytoskeleton, nuclei, and lysosomes), so that the intracellular localization of TRITC capsules could be detected by means of CLSM images [13]. In this way internalized capsules surrounded by lysosomal membrane, could be distinguished from capsules remaining in the extracellular medium [13]. For the immunostaining procedure, the cells were washed with PBS and fixed with 4% paraformaldehyde solution in PBS (20 min incubation at room temperature), followed by washing with PBS. The cells were washed thrice with Hanks' balanced salt solution (HBSS; PBS would have served the same purpose), and permeabilized (addition of glycin 5 mg/mL and saponin 0.5 mg/mL in PBS, in the following referred to as permeabilization solution, 5 min incubation). Then the cells were incubated at 37 °C in an incubator under 5% CO_2 supply with blocking solution (20 mg/mL BSA in permeabilization solution) for 30 min. Next, the lysosomes were immunostained by LAMP 1 (primary antibody; 5 µg/mL blocking sloution, 1 h incubation at 37 °C followed by 3 times washing with blocking solution) and Dylight 649 (secondary antibody; 1.25 µg/mL PBS, 1 h incubation at 37 °C followed by 3 times washing with PBS). In order to save time the staining agents phalloidin labelled with oregon green 488 (20 µg/mL) and Hoechst reagent (for staining of the cytoskeleton and nuclear membranes, respectively) were added within the secondary antibody solution in PBS to cells. The immunostained cells were washed with PBS, followed by water, dried, and fixed on glass slides by means of fluoromount G. The samples were kept in dark at room temperature for 48 h before analyzing

by CLSM. CLSM images were captured using a 63x/1.40 oil-immersion DIC M27 lens. For visualization of different stained cellular compartments and internalized capsules, the fixed samples were excited at 405, 488, 543 and 633 nm, respectively. Hoechst 33342 stained nuclei were excited at 405 nm, and emission of the dye was observed between 420 and 480 nm. The cytoskeleton stained with phalloidin, oregon green 488 was visualized by exciting the fluorophore at 488 nm, and emission was observed between 505 and 550 nm. The fluorescence of TRITC was excited at 543 nm, and emission was recorded using a 560 nm long pass filter. The antibody-labeled lysosomes were excited at 633 nm, and their emission was recorded using a 650 nm long pass filter. Per sample 25 images were captured, covering an average of 100 cells per condition. Experiments were performed in triplicate. A representative CLSM image of TRITC capsules after 24 h incubation with HeLa cells (after immunostaining) is shown in Figure SI-II.2.2b.

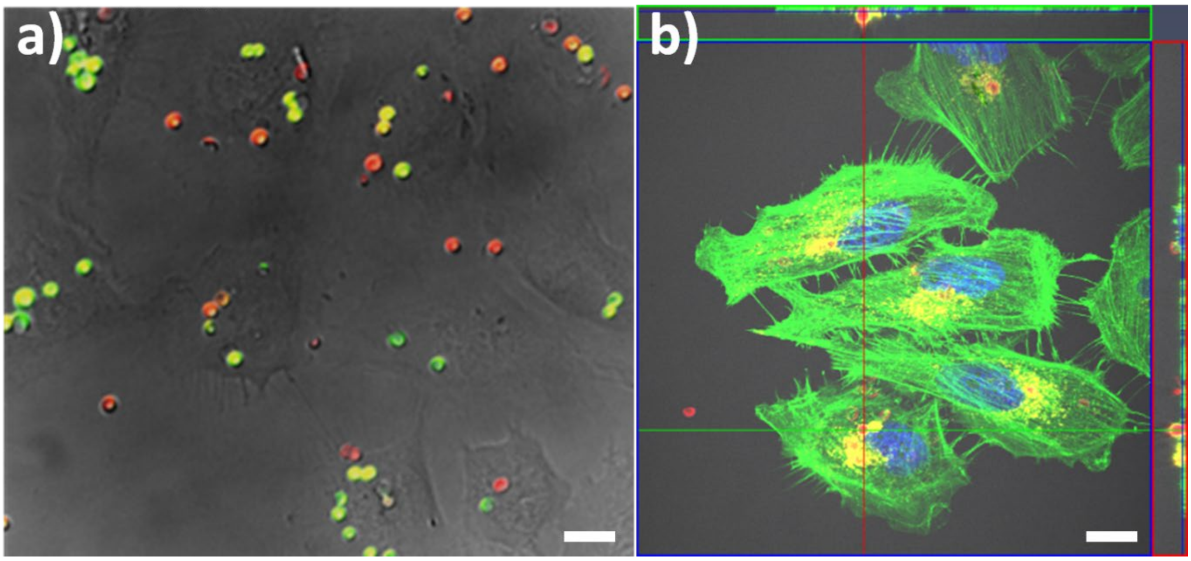

Figure SI-II.2.2: Representative a) fluorescence microscopy and b) CLSM images of HeLa cells exposed to SNARF-loaded and TRITC-loaded capsules, respectively. The image in a) was captured after 2 h of incubation with SNARF-loaded negatively charged capsules at 10 capsules added per seeded cell in serum supplemented media. From the color change of the SNARF-loaded capsules (red to yellow (shown in green false-colors)), the extracellular capsules can be discriminated from the intracellular capsules. The scale bar corresponds to 10 µm. In b) the HeLa cells were incubated with TRITC-loaded capsules, negatively charged capsules at 10 capsules added per seeded cell in serum supplemented media, and the image was taken after 24 h incubation. From the orthogonal view the presence of TRITC-loaded capsules inside the stained lysosomes can be seen. The scale bar corresponds to 10 µm.

II.3) Uptake studies based on capsule counting

In order to evaluate the uptake of capsules inside HeLa cells, first the microscopy images were exported and their format was changed from ".ZVI" to ".JPG". The number of internalized capsules per cell $N_{caps/cell}$ was then manually counted for each time point of incubation for each condition [14]. In case of SNARF-loaded capsules the color change from red to yellow fluorescence was used as indicator of internalization [15]. The internalization of TRITC-loaded capsules verified in the orthogonal CLSM images of immunostained samples by the presence of capsules within stained lysosomal compartments [8, Parakhonskiy, 2015 #32632]. For each condition by counting, a histogram in which the number of cells $f(N_{caps/cell})$ which had internalized $N_{caps/cell}$ capsules was made. From this, cumulative probability / cumulative distribution function (CDFs) $p(N_{caps/cell})$ was calculated [8, 13], see Figure SI-II.3.1:

$$p(N_{caps/cell}) = \frac{\sum_{i=0}^{N_{caps/cell}} f(i)}{\sum_{i=0}^{\infty} f(i)}\,;\ 0 \leq p(N_{caps/cell}) \leq 1 \qquad \text{(Equation SI-II.3.1)}$$

$p(N_{caps/cell})$ is the probability that a cell has internalized not more than $N_{caps/cell}$ capsules per cell (i.e. less than $N_{caps/cell}$ + 1 capsule). 1 - $p(N_{caps/cell})$ is the probability that a cell has internalized more than $N_{caps/cell}$ capsules per cell (i.e. at least $N_{caps/cell}$ + 1 capsule per cell).

From the histograms the mean number of internalized capsules per cell $\langle N_{caps/cell}\rangle_{(h)}$ (t) at each time point t can be calculated by summing up the intensities of all capsules.

The normalized fluorescence intensity I inside one cell due to the fluorescence of capsules is proportional to $\langle N_{caps/cell}\rangle_{(h)}$ (t).

$$\langle I(t)\rangle_{(h)} \propto \langle N_{caps/cell}\rangle_{(h)}(t) = \sum_{i=0}^{\infty} f(i,t) \cdot i \qquad \text{(Equation SI-II.3.2)}$$

Alternatively, from the CDFs the mean number of capsules $\langle N_{caps/cell}\rangle_{(p)}$ (t) that were internalized with 50% probability (i.e. $p(\langle N_{caps/cell}\rangle_{(p)}(t)) = 0.5$) after incubation time t was derived. The way to calculate this number was explained in Figure SI-III.4.1 from Zyuzin et al [16].

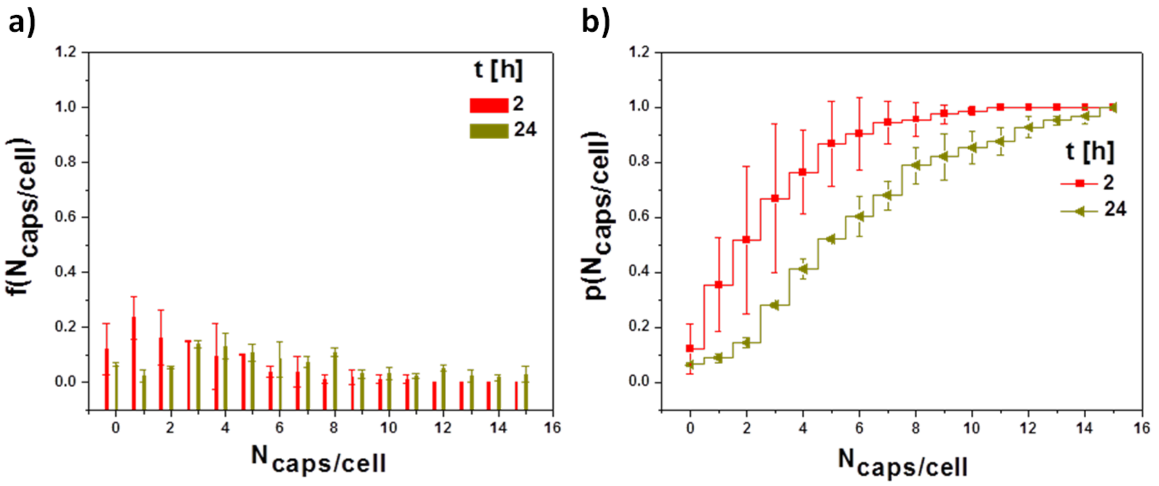

Figure SI-II.3.1: Calculation of cumulative distribution functions from the histograms of capsule uptake. a) Internalization of SNARF-loaded positively charged capsules in terms of histograms $f(N_{caps/cell})$, and b) cumulative probability plots $p(N_{caps/cell})$ in serum supplemented cell culture media as recorded at 2 and 24 hrs of incubation. Cells were incubated with 10 capsules per seeded cell. The data is presented as mean of 2 independent experiments ± standard deviations.

II.4) Results

The uptake results of PEM capsules by HeLa cells for all conditions are presented in the form of histograms (Figure SI-II.4.1, Figure SI-II.4.3) and as well as CDFs obtained as mean of 2 independent experiments ± standard deviations (Figure SI-II.4.2, Figure SI-II.4.4).

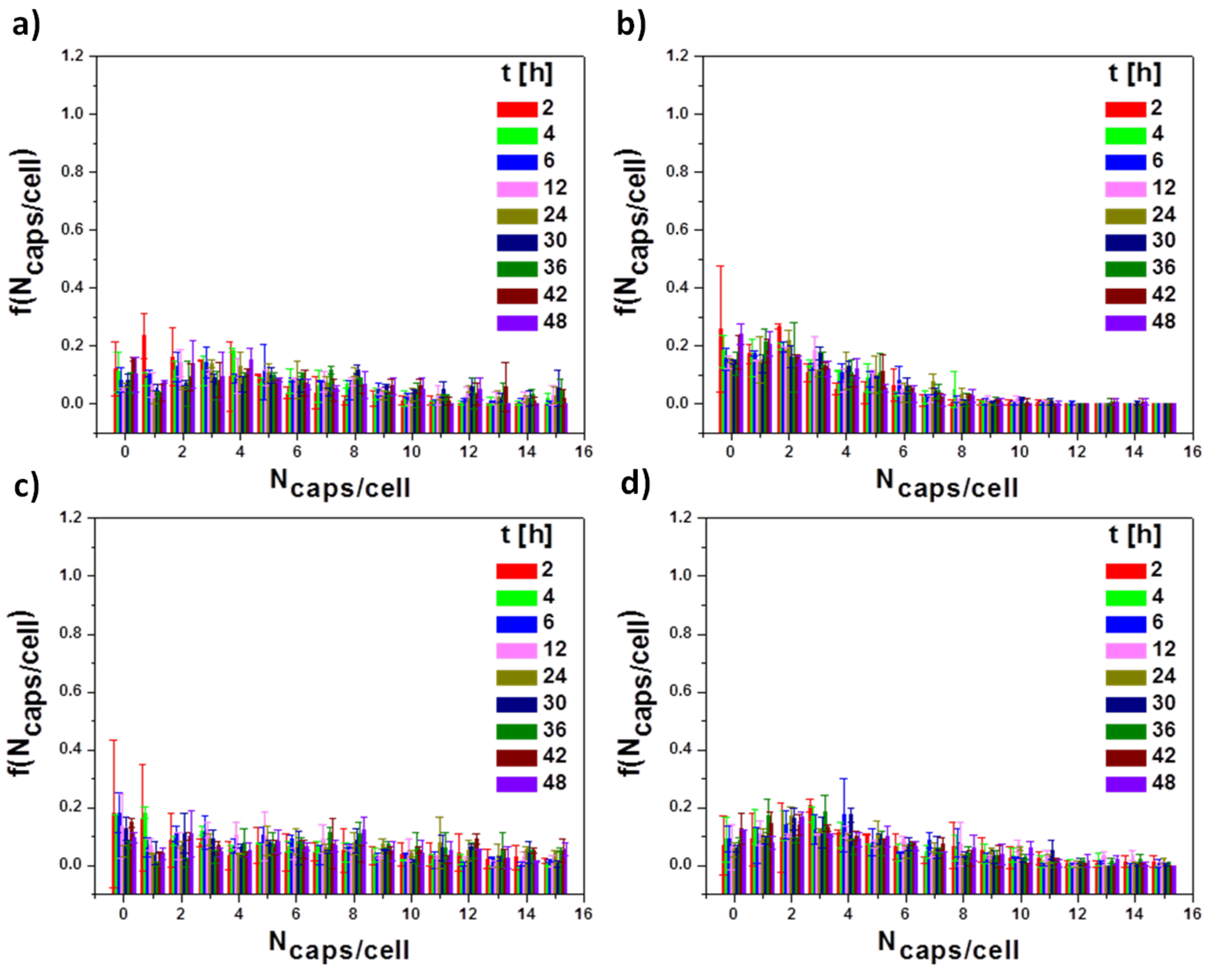

Figure SI-II.4.1: Internalization of SNARF-loaded capsules in terms of histograms $f(N_{caps/cell})$ in serum supplemented cell culture media as recorded at different time points t. a, c) Positively charged capsules. b, d) Negatively charged capsules. a, b) Cells were incubated with 10 capsules per seeded cell. c, d) Cells were incubated with 20 capsules per seeded cell. The data for different incubation times t is presented as mean of 2 independent experiments ± standard deviations.

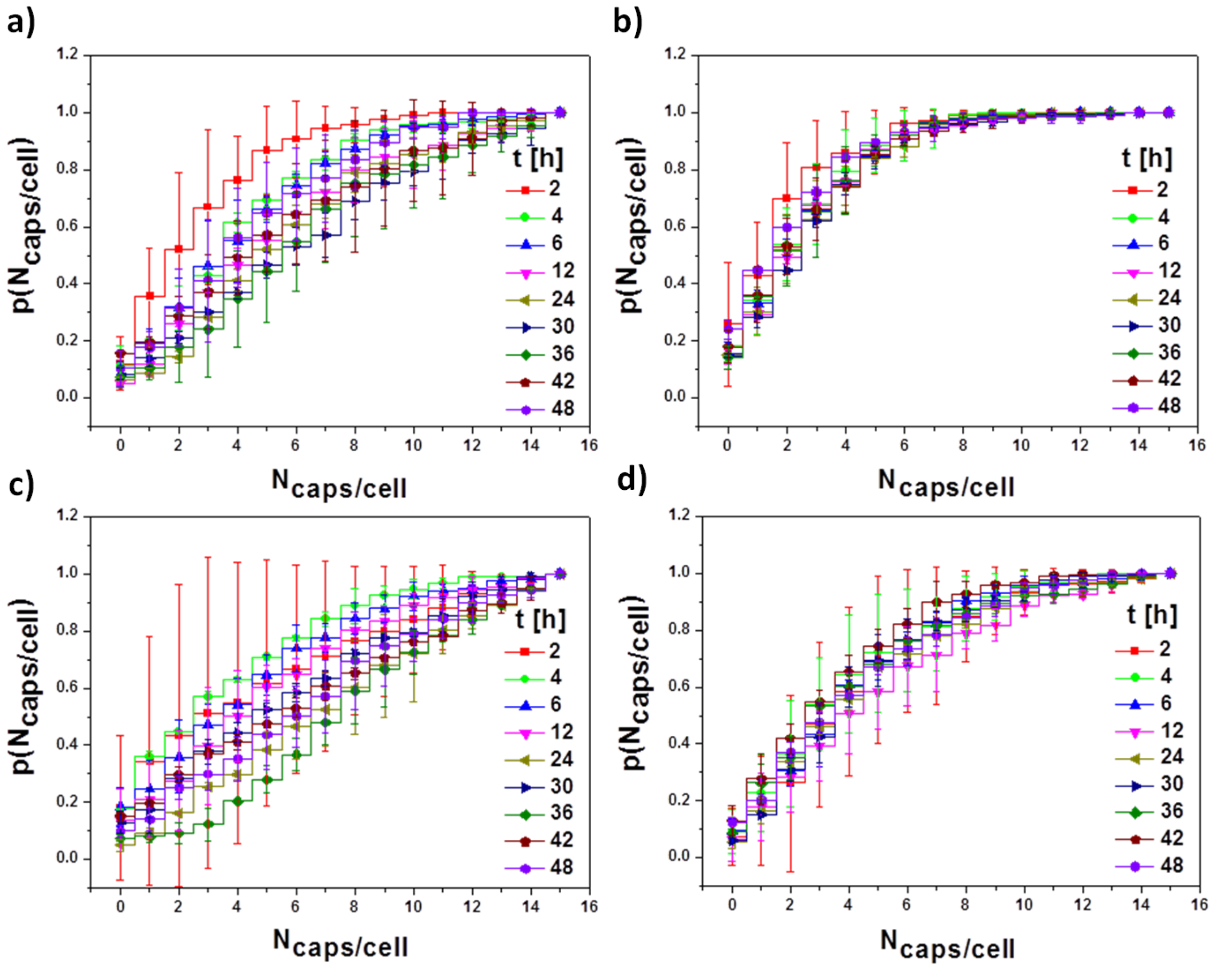

Figure SI-II.4.2: Internalization of SNARF-loaded capsules in terms of cumulative probability plots $p(N_{caps/cell})$ in serum supplemented cell culture media. Data correspond to the histograms shown in Figure SI-II.4.1. a, c) Positively charged capsules. b, d) Negatively charged capsules. a, b) Cells were incubated with 10 capsules per seeded cell. c, d) Cells were incubated with 20 capsules per seeded cell. The data for different incubation times t is presented as mean of 2 independent experiments ± standard deviations.

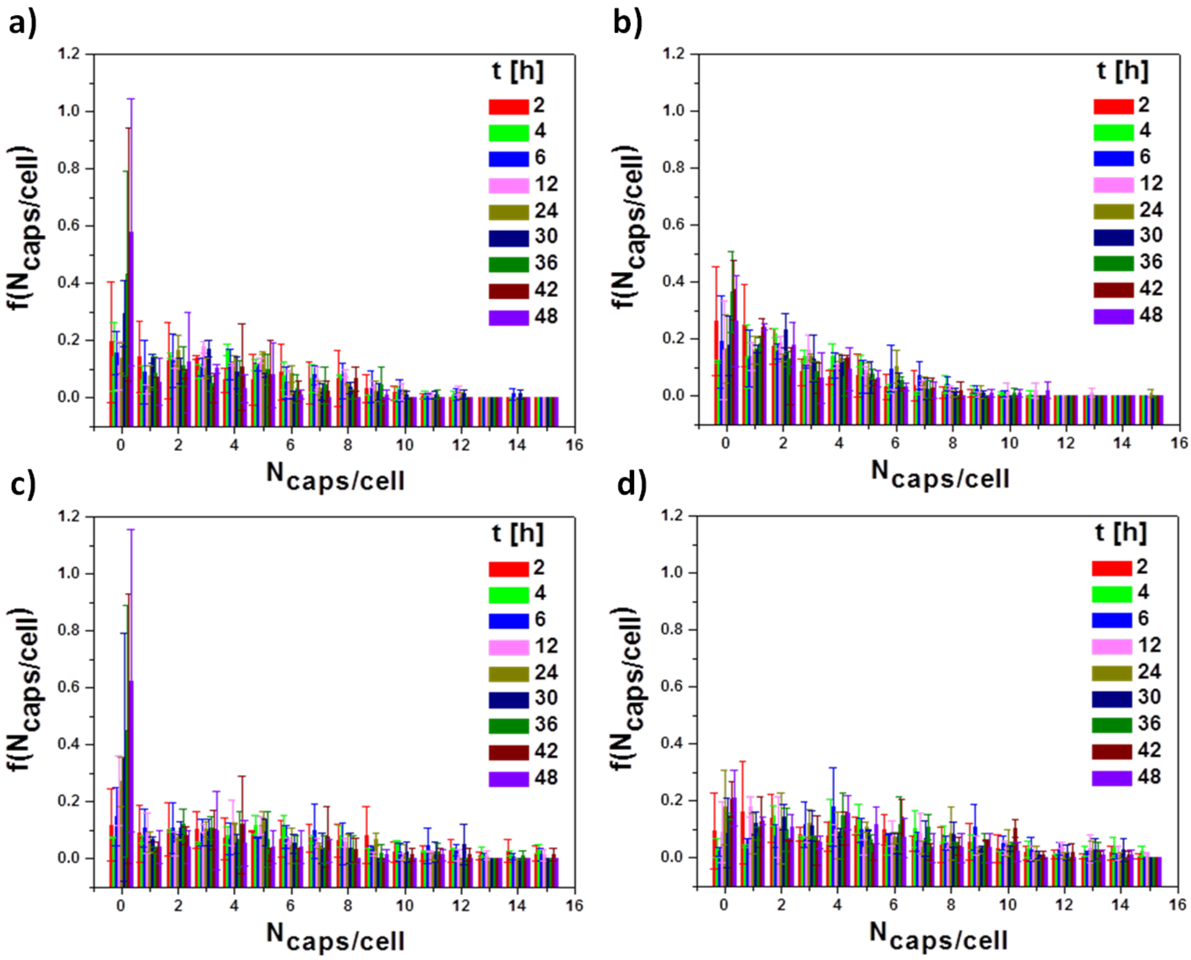

Figure SI-II.4.3: Internalization of SNARF-loaded capsules in terms of histograms f($N_{caps/cell}$) in serum-free cell culture media as recorded at different time points t. a, c) Positively charged capsules. b, d) Negatively charged capsules. a, b) Cells were incubated with 10 capsules per seeded cell. c, d) Cells were incubated with 20 capsules per seeded cell. The data for different incubation times t is presented as mean of 2 independent experiments ± standard deviations.

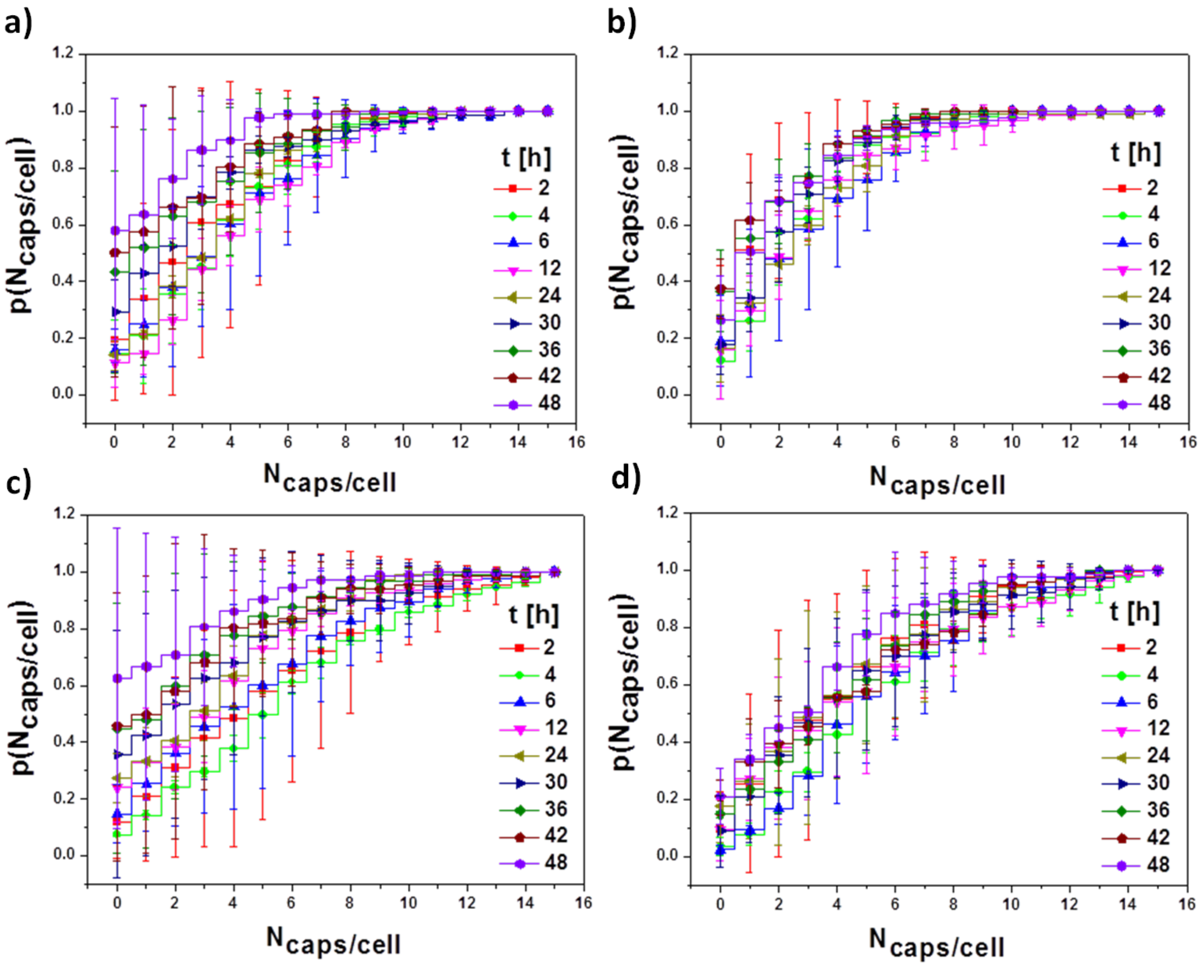

Figure SI-II.4.4: Internalization of SNARF-loaded capsules in terms of cumulative probability plots $p(N_{caps/cell})$ in serum-free cell culture media. Data correspond to the histograms shown in Figure SI-II.4.3. a, c) Positively charged capsules. b, d) Negatively charged capsules. a, b) Cells were incubated with 10 capsules per seeded cell. c, d) Cells were incubated with 20 capsules per seeded cell. The data for different incubation times t is presented as mean of 2 independent experiments $\pm$ standard deviations.

The time dependence of the mean number of capsules internalized per cell $\langle N_{caps/cell}\rangle$ (t) as calculated with the different methods is plotted in Figures SI-II.4.5 and SI-II.4.6 [16] and summarized in Tables SI-II.4.1 and SI-II.4.4. From these curves the maximum number of internalized capsules per cell under saturation conditions $\langle N_{caps/cell}\rangle_{(sat)}$ can be obtained, i.e. the mean value of $\langle N_{caps/cell}\rangle$ (t) for times t at which the number of capsules per cell is saturated, see Tables SI-II.4.2 and SI-II.4.5. Furthermore, the mean time $t_{up(sat)}$ can be determined from Figures SI-II.4.5 and SI-II.4.6, that it takes until cells have internalized $50\% \cdot \langle N_{caps/cell}\rangle_{(sat)}$ capsules per cell.

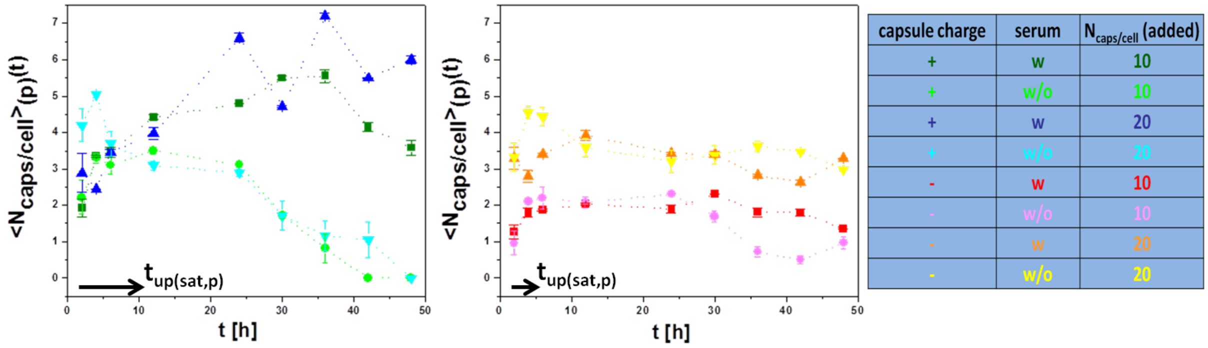

Figure SI-II.4.5: Internalization of SNARF-loaded capsules in terms of the mean numbers of capsules per cell $<N_{caps/cell}>_{(p)}$ (t) that have been internalized with 50% probability (i.e. $p(N_{caps/cell})) = 0.5$), versus incubation times t in serum supplemented and serum deprived cell culture media. Data was derived from the CDFs in Figure SI-II.4.2 and Figure SI-II.4.4. The data is presented as mean of 2 independent experiments ± standard deviations.

charge	+	+	+	+	-	-	-	-
serum	w	w/o	w	w/o	w	w/o	w	w/o
$N_{caps/cell}$ (added)	10	10	20	20	10	10	20	20
t [h]	$<N_{caps/cell}>_{(p)}$	$<N_{caps/cell}>_{(p)}$	$<N_{caps/cell}>_{(p)}$	$<N_{caps/cell}>_{(p)}$	$<N_{caps/cell}>_{(p)}$	$<N_{caps/cell}>_{(p)}$	$<N_{caps/cell}>_{(p)}$	$<N_{caps/cell}>_{(p)}$
2	1.9 ± 0.3	2.5 ± 0.5	2.9 ± 0.5	4.2 ± 0.5	1.3 ± 0.2	1.0 ± 0.3	3.3 ± 0.3	3.3 ± 0.4
4	3.4 ± 0.0	3.3 ± 0.14	2.5 ± 0.0	5.0 ± 0.1	1.8 ± 0.1	2.1 ± 0.1	2.8 ± 0.2	4.6 ± 0.2
6	3.5 ± 0.1	3.1 ± 0.25	3.5 ± 0.1	3.7 ± 0.3	1.9 ± 0.03	2.2 ± 0.3	3.4 ± 0.0	4.4 ± 0.3
12	4.4 ± 0.1	3.5 ± 0.1	4.0 ± 0.2	3.1 ± 0.1	2.0 ± 0.0	2.1 ± 0.1	3.9 ± 0.1	3.6 ± 0.2
24	4.8 ± 0.1	3.1 ± 0.0	6.6 ± 0.1	2.9 ± 0.1	1.9 ± 0.1	2.3 ± 0.1	3.4 ± 0.0	3.2 ± 0.3
30	5.5 ± 0.1	1.7 ± 0.1	4.7 ± 0.0	1.7 ± 0.4	2.3 ± 0.0	1.7 ± 0.2	3.4 ± 0.1	3.4 ± 0.3
36	5.6 ± 0.2	0.8 ± 0.4	7.2 ± 0.1	1.2 ± 0.	1.8 ± 0.1	0.7 ± 0.1	2.8 ± 0.0	3.6 ± 0.2
42	4.2 ± 0.1	0.0 ± 0.0	5.5 ± 0.0	1.1 ± 0.5	1.8 ± 0.1	0.5 ± 0.1	2.6 ± 0.1	3.5 ± 0.0
48	3.6 ± 0.2	0.0 ± 0.0	6.0 ± 0.1	0.0 ± 0.0	1.4 ± 0.0	1.0 ± 0.2	3.3 ± 0.0	3.0 ± 0.0

Table SI-II.4.1: Values for $<N_{caps/cell}>_{(p)}$ (t) as displayed in Figure SI-II.4.5.

charge	+	+	+	+	-	-	-	-
serum	w	w/o	w	w/o	w	w/o	w	w/o
$N_{caps/cell}$ (added)	10	10	20	20	10	10	20	20
$<N_{caps/cell}>_{(sat,p)}$	5.6	3.5	6.9	5.0	2.3	2.3	3.9	4.6

Table SI-II.4.2: Mean number of capsules per cell under saturation conditions $<N_{caps/cell}>_{(sat,p)}$, as derived from Figure SI-II.4.5.

charge	+	+	+	+	-	-	-	-
serum	w	w/o	w	w/o	w	w/o	w	w/o
$N_{caps/cell}$ (added)	10	10	20	20	10	10	20	20
$t_{up(sat,p)}$ [h]	3	2	6	1	2	2	1	1

Table SI-II.4.3: Time $t_{up(sat)}$ until 50% of the mean number of capsules per cell under saturation conditions $0.5 \cdot \langle N_{caps/cell} \rangle_{(sat,p)}$ has been internalized, as derived from Figure SI-II.4.5.

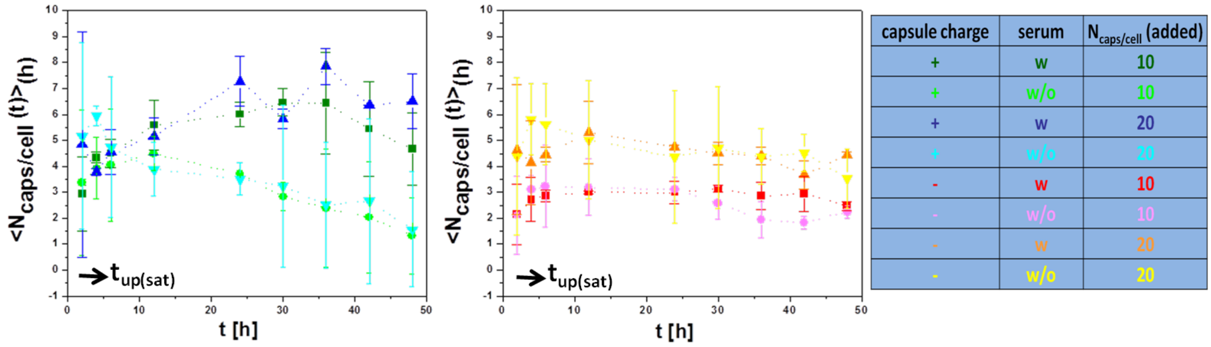

Figure SI-II.4.6: Internalization of SNARF-loaded capsules in terms of the mean numbers of capsules per cell $\langle N_{caps/cell} \rangle_{(h)}$ (t) as calculated from the histograms by using Equation SI-II.3.2. Data was derived from the histograms in Figure SI-II.4.1 and Figure SI-II.4.3. The data is presented as mean of 2 independent experiments ± standard deviations.

charge	+	+	+	+	-	-	-	-
serum	w	w/o	w	w/o	w	w/o	w	w/o
$N_{caps/cell}$ (added)	10	10	20	20	10	10	20	20
t [h]	$\langle N_{caps/cell} \rangle_{(h)}$	$\langle N_{caps/cell} \rangle_{(h)}$	$\langle N_{caps/cell} \rangle_{(h)}$	$\langle N_{caps/cell} \rangle_{(h)}$	$\langle N_{caps/cell} \rangle_{(h)}$	$\langle N_{caps/cell} \rangle_{(h)}$	$\langle N_{caps/cell} \rangle_{(h)}$	$\langle N_{caps/cell} \rangle_{(h)}$
2	2.9 ± 1.4	3.4 ± 2.8	4.8 ± 4.3	5.2 ± 3.6	2.2 ± 1.2	2.1 ± 1.5	4.6 ± 2.5	4.4 ± 3.0
4	4.3 ± 0.2	3.9 ± 1.2	3.8 ± 0.1	6.0 ± 0.4	2.7 ± 0.8	3.1 ± 0.5	4.1 ± 1.6	5.8 ± 1.4
6	4.5 ± 0.6	4.1 ± 2.2	4.5 ± 0.9	4.7 ± 2.7	2.9 ± 0.2	3.2 ± 1.6	4.5 ± 0.3	5.6 ± 1.6
12	5.6 ± 1.0	4.5 ± 0.7	5.3 ± 0.7	3.9 ± 1.0	3.0 ± 0.1	3.2 ± 1.1	5.3 ± 1.2	5.0 ± 2.3
24	6.0 ± 0.5	3.7 ± 0.1	7.3 ± 0.9	3.5 ± 0.6	3.0 ± 0.4	3.1 ± 0.4	4.7 ± 0.1	4.3 ± 2.6
30	6.5 ± 0.5	2.8 ± 0.5	5.9 ± 0.4	3.2 ± 3.1	3.2 ± 0.1	2.6 ± 0.6	4.5 ± 0.4	4.7 ± 2.4
36	6.4 ± 1.9	2.4 ± 2.3	7.9 ± 0.7	2.5 ± 2.4	2.9 ± 0.5	1.9 ± 0.7	4.4 ± 0.4	4.4 ± 1.1
42	5.4 ± 1.8	2.0 ± 2.1	6.4 ± 0.0	2.7 ± 3.2	2.9 ± 0.7	1.8 ± 0.2	3.7 ± 0.6	4.5 ± 0.7
48	4.7 ± 1.4	1.3 ± 1.5	6.5 ± 1.1	1.6 ± 2.2	2.5 ± 0.2	2.3 ± 0.2	4.5 ± 0.1	3.5 ± 1.2

Table SI-II.4.4: Values for $\langle N_{caps/cell} \rangle_{(h)}$ (t) as displayed in Figure SI-II.4.6.

charge	+	+	+	+	-	-	-	-
serum	w	w/o	w	w/o	w	w/o	w	w/o
$N_{caps/cell}$ (added)	10	10	20	20	10	10	20	20
$<N_{caps/cell}>_{(sat,h)}$	6.5	4.5	7.9	6.0	3.2	3.2	5.3	5.6

Table SI-II.4.5: Mean number of capsules per cell under saturation conditions $<N_{caps/cell}>_{(sat,h)}$, as derived from Figure SI-II.4.6.

charge	+	+	+	+	-	-	-	-
serum	w	w/o	w	w/o	w	w/o	w	w/o
$N_{caps/cell}$ (added)	10	10	20	20	10	10	20	20
$t_{up(sat,h)}$ [h]	3	1	< 2	< 2	< 2	< 2	< 2	< 2

Table SI-II.4.6: Time $t_{up(sat)}$ until 50% of the mean number of capsules per cell under saturation conditions $0.5 \cdot <N_{caps/cell}>_{(sat,h)}$ has been internalized, as derived from Figure SI-II.4.6.

In Figure SI-II.4.7, the time-dependent probability that each cell has at least internalized one capsule: 1 - p($N_{caps/cell}$= 0). Here the time $t_{up(1)}$ until 1 - p($N_{caps/cell}$= 0) = 50% can be defined as time required for the uptake of one capsule. Values for $t_{up(1)}$ are given in Table SI-II.4.7.

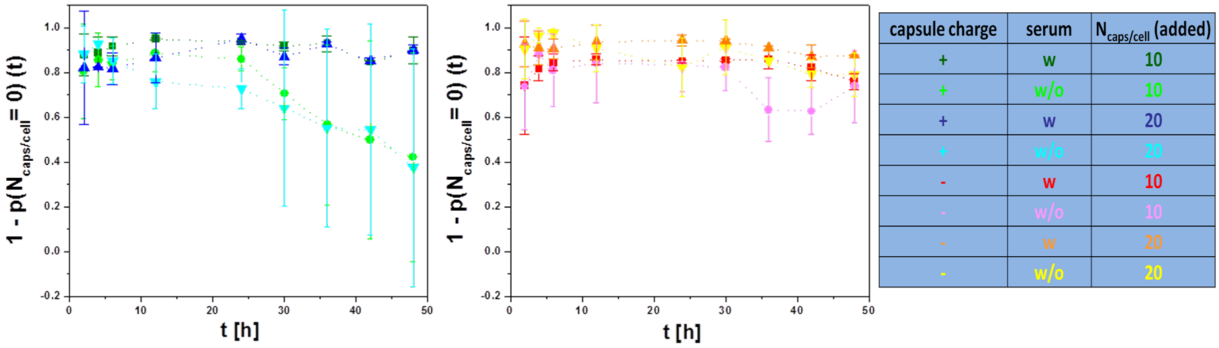

Figure SI-II.4.7: 1 - p($N_{caps/cell}$= 0) (t) for SNARF-loaded capsules. Data taken from Figure SI-II.4.2 and SI-II.4.4.

charge	+	+	+	+	-	-	-	-
serum	w	w/o	w	w/o	w	w/o	w	w/o
$N_{caps/cell}$ (added)	10	10	20	20	10	10	20	20
$t_{up(1)}$ [h]	< 1	< 1	< 1	< 1	< 1	< 1	< 1	< 1

Table SI-II.4.7: Values for $t_{up(1)}$ as derived from Figure SI-II.4.7.

Finally in Figure SI-II.4.8 data for TRITC-loaded (instead of SNARF-loaded) capsules are shown.

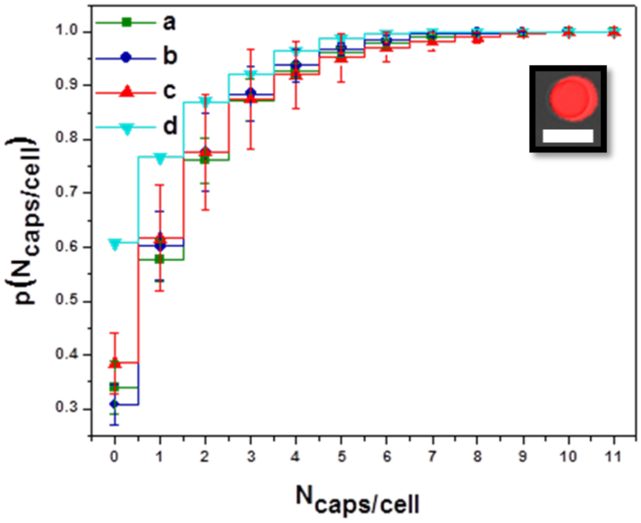

Figure SI-II.4.8: Internalization of negatively and positively charged TRITC-loaded capsules (2 bilayers) in terms of cumulative probability plots $p(N_{caps/cell})$ after t = 24 h incubation, having added 10 capsules per seeded cell. The data is presented as mean of 3 independent experiments ± standard deviations (only the data involving Bafilomycin A1 are based on one single experiment). a) Positively charged capsules, serum-supplemented medium. b) Positively charged capsules, serum-free medium. c) Negatively charged capsules, serum-supplemented medium. d) Positively charged capsules, serum-supplemented medium, with addition of Bafilomycin A1. In inset a CLSM image of one TRITC-loaded capsule (positively charged, 2 bilayers) is provided. The scale bar corresponds to 5 µm. The data demonstrate that presence of Bafilomycin A1 reduces uptake of capsules by cells.

III) Flow cytometry analysis of SNARF capsules internalized by cells

III.1) Materials, reagents, and equipment
III.2) Synthesis and calibration of SNARF-loaded polyelectrolyte micro- (PEM) capsules
III.3) Uptake studies by flow cytometry
III.4) Results

III.1) Materials, reagents, and equipment

4',6-diamidino-2-phenylindole, dilactate (DAPI, #D3571) was purchased from Invitrogen (Germany). All other reagents, chemicals, consumables were the same as described in sections §I.1 and §II.1.

For flow cytometry a BD LSRFortessa™ cell analyzer flow cytometer (Becton, Dickinson and Company, USA) was used. The fluorescence of the SNARF was excited at 561 nm and its emission was captured using 586 nm/15 nm and 670 nm /30 nm band pass filters for detecting the intensity of yellow and red fluorescence, respectively. Data was analyzed with FlowJo, single cell analysis software (Ashland, OR, USA).

III.2) Synthesis and calibration of SNARF-loaded polyelectrolyte micro- (PEM) capsules

The method for the fabrication of SNARF-loaded capsules was the same as described in section §I.2.

Calibration curves of capsules immersed in different pH were recorded with flow cytometry, in order to distinguish between populations of capsules surrounded by medium with different pH [15]. For this positively and negatively charged SNARF-loaded capsules were mixed with buffers having a range of pH values from 3 - 10. Solutions were analyzed with flow cytometry and 10,000 events per sample were recorded by exciting SNARF at 561 nm and recording its emissions at 586 nm and 670 nm. Results are plotted in different presentations. In Figure SI-III.2.1c,d and Figure SI-III.2.2c,d the distribution of the fluorescence intensities $f(I_y)$ and $f(I_r)$ as recorded in the yellow and the red channel are shown. As expected, the yellow and the red fluorescence decreases and increases upon raising the pH. In Figure SI-III.2.1b and Figure SI-III.2.2b the distribution of the capsule populations are plotted in a 2-dimensional graph (density plot), which corresponds to a convolution of the fluorescence distributions of the yellow and red channel. The data demonstrate, that capsules immersed in different pH can be identified as distinct populations in the I_y versus I_r density plots. Finally in Figure SI-III.2.3 the ratio of the mean intensities $\langle I_r \rangle / \langle I_y \rangle$ as derived from the intensity distributions $f(I_y)$ and $f(I_r)$ given in Figure SI-III.2.1c,d and Figure SI-III.2.2c,d is shown. This results in the typical red-to-yellow calibration curve of SNARF. In comparison to the CLSM study (i.e. Figure SI-I.2.) the absolute values are different, due to the different filter-sets which have been used. As additional control in Figure SI-III.2.1a and Figure SI-III.2.2a, the capsule populations are shown

in the density plot of forward scattering (FSC) versus side scattering (SSC) intensities I_{FSC} and I_{SSC}.

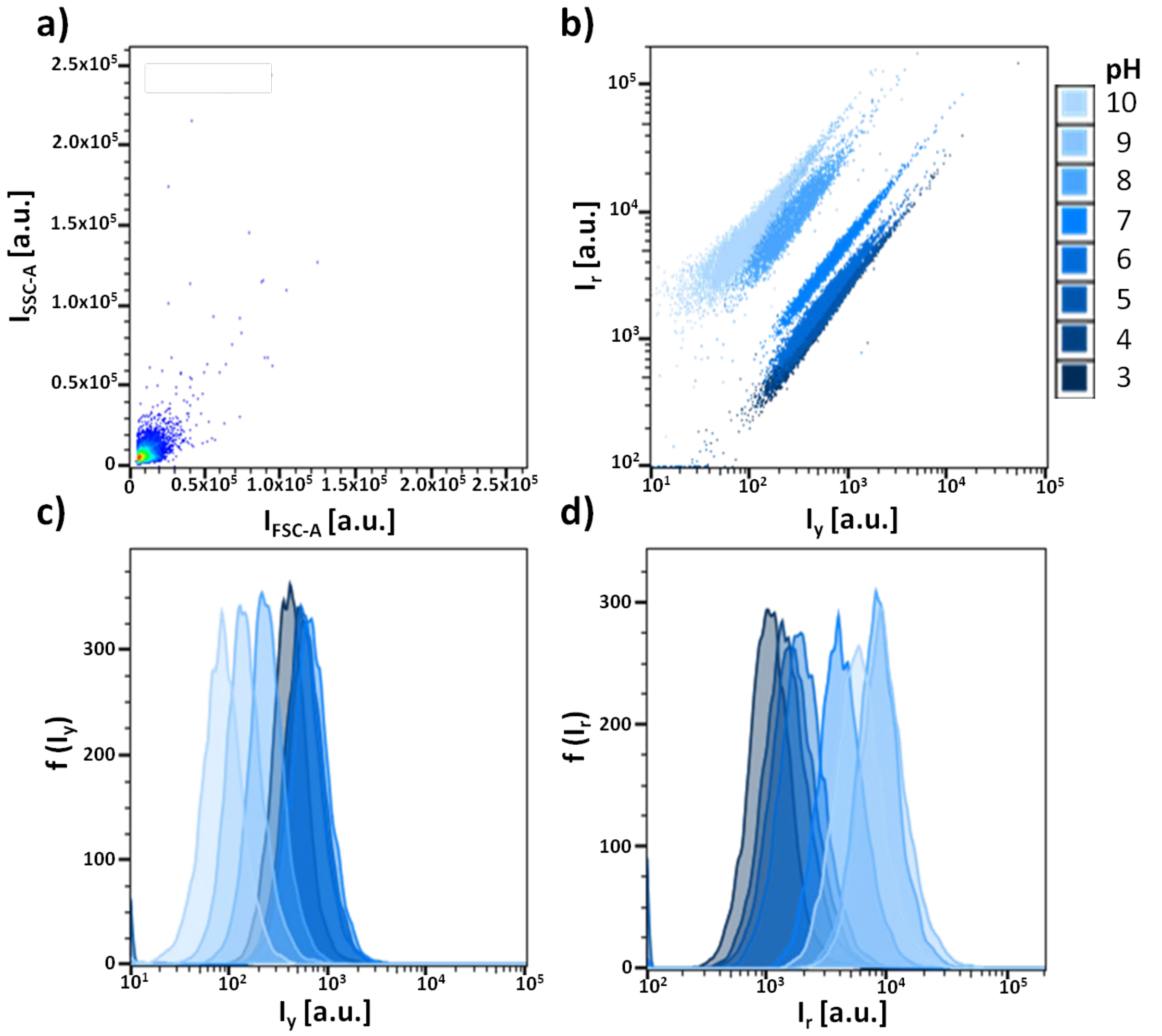

Figure SI-III.2.1: Flow cytometer investigation of positively charged SNARF-loaded capsules immersed in various pH buffers. a) The capsule distribution sorted by their forward scattering (FSC-A; A = area) and side scattering (SSC-A; A = area) intensities I_{FSC} and I_{SSC} recorded at pH = 7. Each dot corresponds to one recorded capsule detection event. b) The capsule distribution sorted by their fluorescence intensities in the red and yellow channel, I_r and I_y, respectively, as recorded at different pH values. Each dot corresponds to one recorded capsule detection event. c) Distribution $f(I_y)$ of the capsule fluorescence in the yellow, recorded as counts per fluorescence intensity, as obtained at different pH values. d) Distribution $f(I_r)$ of the capsule fluorescence in the red, recorded as counts per fluorescence intensity, as obtained at different pH values.

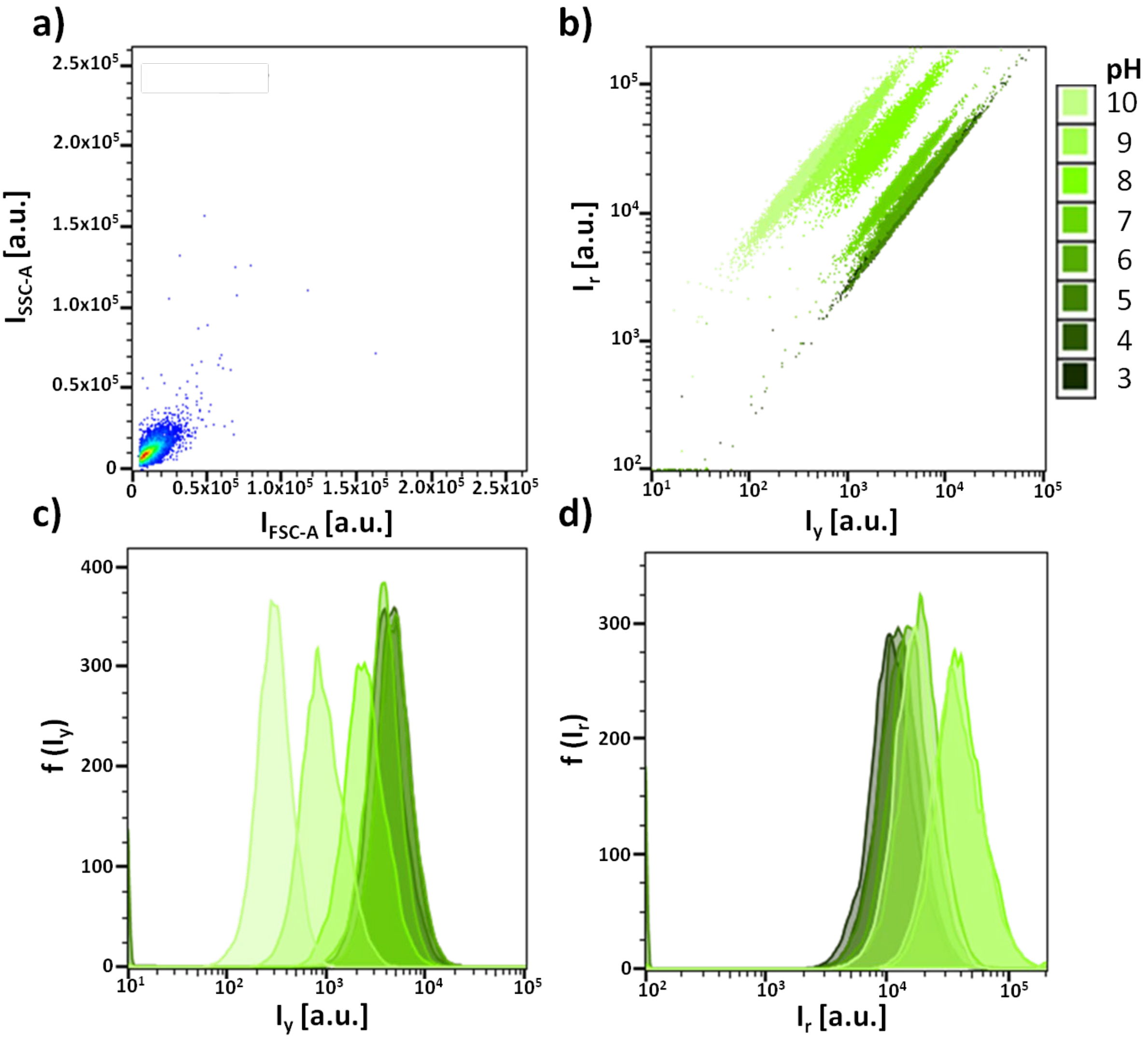

Figure SI-III.2.2: Flow cytometer investigation of negatively charged SNARF-loaded capsules immersed in various pH buffers. a) The capsule distribution sorted by their forward scattering (FSC-A) and side scattering (SSC-A) intensities I_{FSC} and I_{SSC} recorded at pH = 7. Each dot corresponds to one recorded capsule detection event. b) The capsule distribution sorted by their fluorescence intensities in the red and yellow channel, I_r and I_y, respectively, as recorded at different pH values. Each dot corresponds to one recorded capsule detection event. c) Distribution $f(I_y)$ of the capsule fluorescence in the yellow, recorded as counts per fluorescence intensity, as obtained at different pH values. d) Distribution $f(I_r)$ of the capsule fluorescence in the red, recorded as counts per fluorescence intensity, as obtained at different pH values.

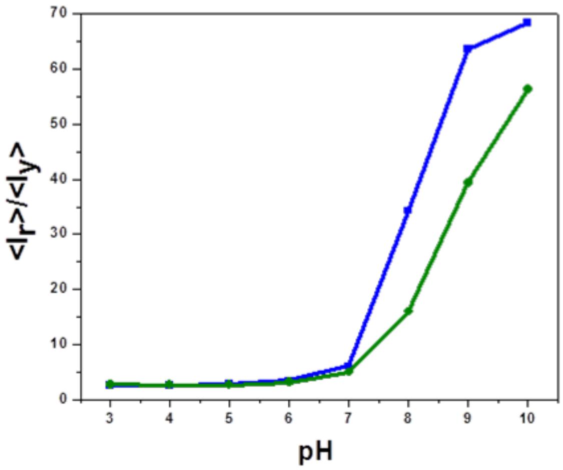

Figure SI-III.2.3: Ratio of the mean fluorescence intensities $<I_r>/<I_y>$ as derived from the intensity distributions $f(I_r)$ and $f(I_y)$ for the different pH values. Data are shown for positively (blue) and negatively (green) charged capsules.

III.3) Uptake studies by flow cytometry

For investigation capsule uptake by cells, HeLa cells were seeded in 24 well plates (growth area 1.82 cm^2 /well) at 40,000 cells seeded per well in 0.6 mL complete cell growth media. Cells were kept inside an incubator set to 37 °C with 5% CO_2. After 24 h the cells were provided fresh growth media (either serum supplemented or serum deprived) containing positively or negatively charged SNARF-loaded capsules at a density of 10 or 20 capsules added per seeded cell. Cells which were not exposed to capsules served as control. The cells were incubated with the capsules for different times, i.e., 2, 4, 6, 12, 24, 30, 36, 42, and 48 h. After incubation cells were washed with PBS, trypsinized, and resuspended in PBS. For viability assessment, DAPI (2 µL, 1.09 mM) was added to each sample. The samples were then investigated with flow cytometry. The forward (FSC-A) and sideward (SSC-A) scattering signals were used to gate events involving cells, i.e. only events with sufficient scattering signal were further regarded. This gating, which in Figure SI-III.3.1 is referred to as G1, excludes event due to cellular debris and free capsules, which have a much lower scattering signal than cells (compare with Figure SI-III.2.1a). By means of a second gating (G2), cell doublets were removed and excluded from the analysis. This was done in the forward scatter plot in which the area (FSC-A) was plotted against width (FSC-W). In order to detect a single cell population, 5000 events/sample were recorded in G2 and were used for further data processing. Using both gates G1 and G2, 2-dimensional density plots of the red and yellow fluorescence signals I_r and I_y were created. In these plots populations of cells with adherent and internalized SNARF-loaded capsules can be distinguished, as internalized capsules are located in acidic environment [15]. Events without sufficient fluorescence were attributed to cells which had neither capsules adherent to their outer wall, nor internalized capsules. In this way from each plot three different cell populations were identified: cells without associated capsules, cells with adherent capsules, and cells with internalized capsules. From the density plots the fractions of the respective

populations were derived according to the amount of detected respective events, see Figure SI-III.3.1.

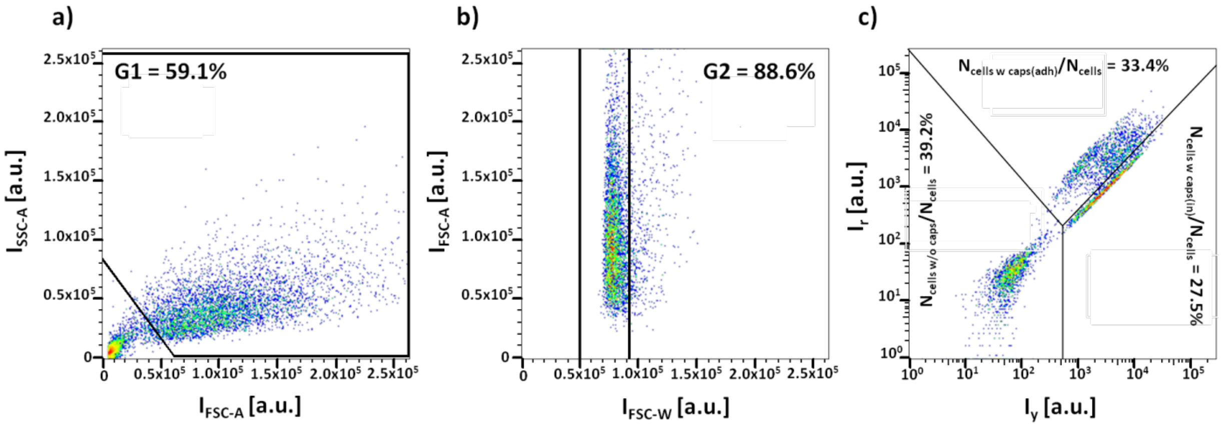

Figure SI-III.3.1: Gating strategy for flow cytometry based uptake studies of SNARF-loaded capsules by HeLa cells as exemplified after 4 h incubation in serum supplemented cell culture media upon the addition of negatively charged capsules at 10 capsules added per seeded HeLa cell. a) The area plots of SSC versus FSC enables the exclusion of cell debris and free capsules by detector voltage adjustment and gating of cell population, i.e., G1. b) Cell doublets were removed by the area versus width plots of FSC and gating of single cell suspension (G2) was performed. In order to collect the single cell population, 5000 events/sample were recorded in G2, which were used for further data processing. c) 2-dimensional density plots of red and yellow fluorescence signals enable to distinguish between the populations of cells with adherent ($N_{cells\ w\ caps(adh)}/N_{cells}$) and internalized capsules ($N_{cells\ w\ caps(in)}/N_{cells}$), by integrating the events above and below the separation line, respectively [15]. The part showing only cells without involving fluorescent SNARF-loaded capsules ($N_{cells\ w/o\ caps}/N_{cells}$) gives information about the population of cells without capsules. Individual gating was adjusted to separate the three different cell populations.

By integrating the number of events in the various regions of the density plots the populations of the fraction of cells without associated capsules ($N_{cells\ w/o\ caps}/N_{cells}$), the fraction of cells with internalized capsules ($N_{cells\ w\ caps(in)}/N_{cells}$), and the fraction of cells with adherent capsules ($N_{cells\ w\ caps(adh)}/N_{cells}$) was determined for each sample per each time point. The number of cells associated with capsules $N_{cells\ w\ caps}$ (either internalized or adherent) is

$$N_{cells\ w\ caps} = N_{cells\ w\ caps(in)} + N_{cells\ w\ caps(adh)} \qquad \text{(Equation SI-III.3.1)}$$

The total number of counted cells N_{cells} is

$$N_{cells} = N_{cells\ w\ caps} + N_{cells\ w/o\ caps} \qquad \text{(Equation SI-III.3.2)}$$

III.4) Results

In Figure SI-III.4.1, the standard uptake curve in which fluorescence intensity per cell due to internalized capsules is plotted versus time is shown. This curve does not used any gating strategy. From this curve the mean intensity $<I_y>_{(sat,c)}$ per cell under saturation and the mean time $t_{up(sat,c)}$ until the mean fluorescence intensity $<I_y>_{(c)}$ per cell has reached 50% of the saturation value can be determined, see Table SI-III.4.1.

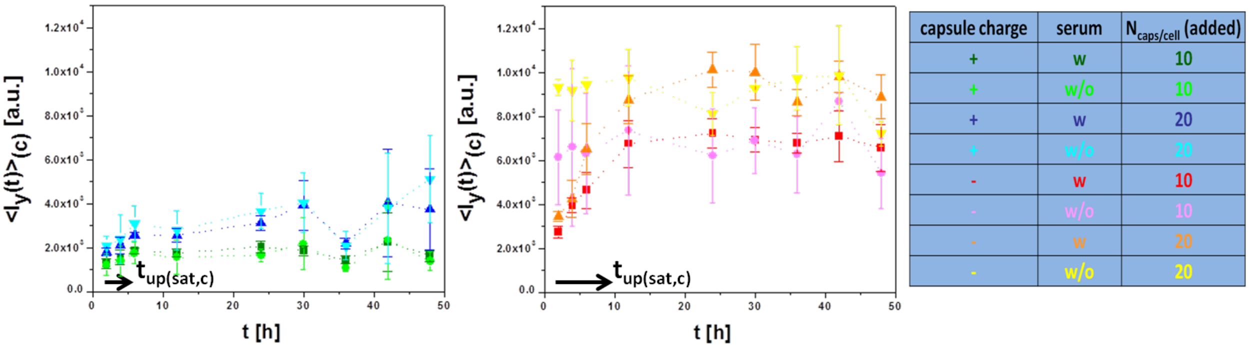

Figure SI-III.4.1: Time dependent mean fluorescence $<I_y(t)>_{(c)}$ in the yellow channel upon uptake of capsules.

charge	+	+	+	+	-	-	-	-
serum	w	w/o	w	w/o	w	w/o	w	w/o
$N_{caps/cell}$ (added)	10	10	20	20	10	10	20	20
$<I_y>_{(sat)}$	2042	2342	4027	5124	7228	8678	10131	9867

Table SI-III.4.1: Mean intensity under saturation conditions $<I_y>_{(sat,c)}$, as derived from Figure SI-III.4.1.

charge	+	+	+	+	-	-	-	-
serum	w	w/o	w	w/o	w	w/o	w	w/o
$N_{caps/cell}$ (added)	10	10	20	20	10	10	20	20
$t_{up(sat,c)}$ [h]	< 2	< 2	2	3	3	< 2	4	< 2

Table SI-III.4.2: Time $t_{up(sat,c)}$ until 50% of the saturation intensity upon capsule internalization has been reached as derived from Figure SI-III.4.1.

In Figure SI-III.4.2, the fractions of cells with internalized, adherent, and without cells are plotted versus time. Experiments were performed in triplicate and data are provided as mean values ± standard deviations. From Figure SI-III.4.2, the percentage of cells with internalized capsules under saturation condition $(N_{cells\ w\ caps(in)}/N_{cells})_{(sat)}$, and the time $t_{up(sat,f)}$ which it takes until (as compared to saturation conditions) half of the amount of cells has internalized capsules can be derived, see Table SI-III.4.3 and Table SI-III.4.4.

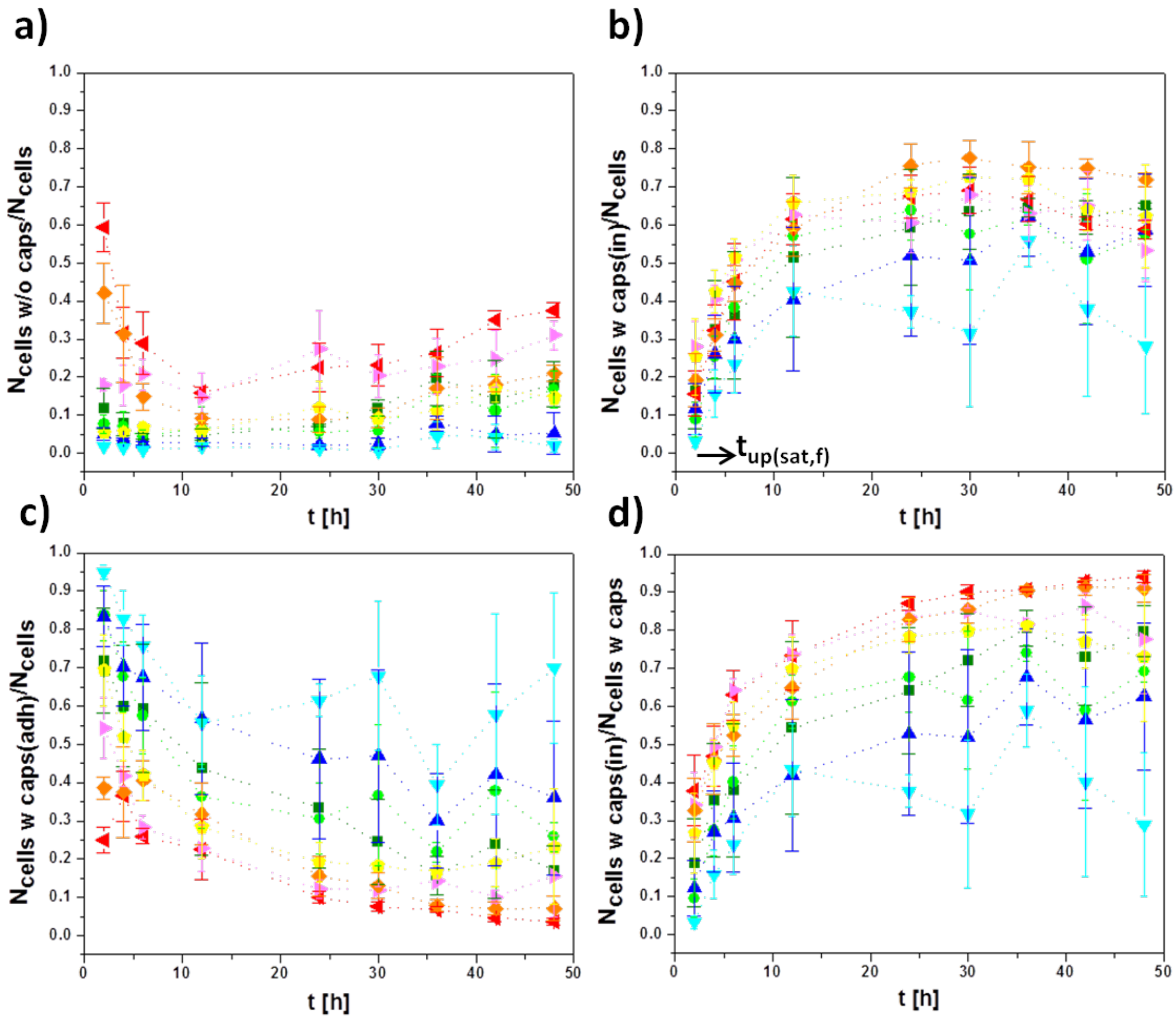

capsule charge	serum	$N_{caps/cell}$ (added)
+	w	10
+	w/o	10
+	w	20
+	w/o	20
-	w	10
-	w/o	10
-	w	20
-	w/o	20

Figure SI-III.4.2: Fraction of cell populations with a) without capsules $N_{cells\ w/o\ caps}/N_{cells}$, b) internalized capsules $N_{cells\ w\ caps(in)}/N_{cells}$, c) adherent capsules $N_{cells\ w\ caps(adh)}/N_{cells}$, and, d) $N_{cells\ w\ caps(in)}/N_{cells\ w\ caps}$ at different time points is shown.

charge	+	+	+	+	-	-	-	-
serum	w	w/o	w	w/o	w	w/o	w	w/o
$N_{caps/cell}$ (added)	10	10	20	20	10	10	20	20
$(N_{cells\ w\ caps(in)}/N_{cells})_{(sat)}$ [%]	65.1	63.83	62.3	55.97	69.23	67.77	77.7	72.7

Table SI-III.4.3: Percentage of cells with internalized capsules under saturation conditions as derived from Figure SI-III.4.2.

charge	+	+	+	+	-	-	-	-
serum	w	w/o	w	w/o	w	w/o	w	w/o
$N_{caps/cell}$ (added)	10	10	20	20	10	10	20	20
$t_{up(sat,f)}$ [h]	4	5	6.5	6	4	3	5	3

Table SI-III.4.4: Time $t_{up(sat,f)}$ until 50% of the saturation intensity upon capsule internalization has been reached as derived from Figure SI-III.4.2.

IV) Elemental analysis of gold nanoparticle (GNP)-loaded capsules internalized by cells

IV.1) Materials and reagents
IV.2) Synthesis and characterization of gold nanoparticle (GNP) -loaded polyelectrolyte micro- (PEM) capsules
IV.3) Uptake studies by inductively coupled plasma mass spectroscopy (ICP-MS)
IV.4) Results

IV.1) Materials and reagents

6 well cell culture plates (# 83.1839.300), eppendorf tubes (2 mL; # 72.695.500), and falcon tubes (15 and 50 mL; #62.554.502, #62.547.254, respectively) from Sarstedt were used. Hydrochloric acid (HCl; 35 wt%, ultra-pure, #7647010) and nitric acid (HNO_3; 67 wt%, ultra pure, #7697372) were purchased from Fisher Chemicals. Cell lysis buffer (5X reagent; #2018-02-12) was used from Promega Corporation. A total protein determination kit (micro-Lowry, Peterson's modification; TPO300-KT, batch# SLBF6513) was purchased from Sigma-Aldrich, consisting of, Lowry reagent powder (2 g; L3540-1VL, SLBD9543), and Folin and Ciocalteu's phenol reagent (F9252-1EA, Lot#SHBB8897V, Pcode: 1001449215). Commercially available gold nanoparticles (GNPs; 15 nm, #EM.GC15) were obtained from BBI solutions. Amino dextran (70 kDa, #D1862) was purchased from Thermo Fisher Scientific. All other reagents, chemicals, and consumables, were the same as described in sections §I.1 and §II.1.

A UV-vis absorption spectrometer (8453 UV-visible spectrophotometer) from Agilent was used for obtaining the absorption values of the protein content in the Lowry tests. An inductively coupled plasma mass spectrometer (ICP-MS) from Agilent 7700 Series was used to determine the concentrations of elemental gold and hence the GNP concentrations.

IV.2) Synthesis and characterization of gold nanoparticle (GNP) -loaded polyelectrolyte micro- (PEM) capsules

In order to fabricate GNP-loaded PEM capsules comparable to SNARF-loaded capsules, the basic strategy for the synthesis was same as that for SNARF-loaded capsules (cf. § I.2). Briefly, amino dextran was co-precipitated with $CaCO_3$ particles, by mixing the solutions of $CaCl_2$ and Na_2CO_3 under vigorous stirring in the presence of amino dextran at room temperature (RT). In a glass vial, 1.4 mL amino dextran (0.5 mg/mL) was added to 1 mL of 0.33 M $CaCl_2$ (0.33 M). Under vigorous magnetic stirring (1100 rpm) 1 mL of Na_2CO_3 (0.33 M) solution was quickly mixed with the above mixture for 30 s followed by keeping the reaction contents intact for 2 min. The $CaCO_3$ particles were washed three times with DDW and used for LBL assembly of oppositely charged polyelectrolytes (2 mg/mL, 0.5 M NaCl). The alternating layers of negatively and positively charged polymers, i.e., PSS and PAH, respectively, were deposited

around the charged sacrificial $CaCO_3$ particle templates. Layer-by-layer deposition was achieved by alternating immersion of the particles inside the respective polyelectrolyte solutions (3 mL) for 13 min, followed by subsequent washing with DDW to remove excess of polymers. Negatively charged GNPs (2.7 mL of the solution as purchased mixed with 0.3 mL 0.5 M NaCl just before layer deposition) were incorporated inside the capsules after the first bilayer (PSS/PAH), followed by the deposition of additional 1 (PSS/PAH) and 1.5 (PSS/PAH/PSS) bilayers for positively and negatively charged capsules, respectively. Finally the cores of the PEM capsules were dissolved by complexion of Ca^{2+} ions with EDTA (3 mL, 0.2 M, pH 6.5) and the resulting capsules were washed with DDW and stored in water at 4 °C for further use. Their hydrodynamic diameter and zeta potentials were recorded and are shown in Figure SI-IV.2.1.

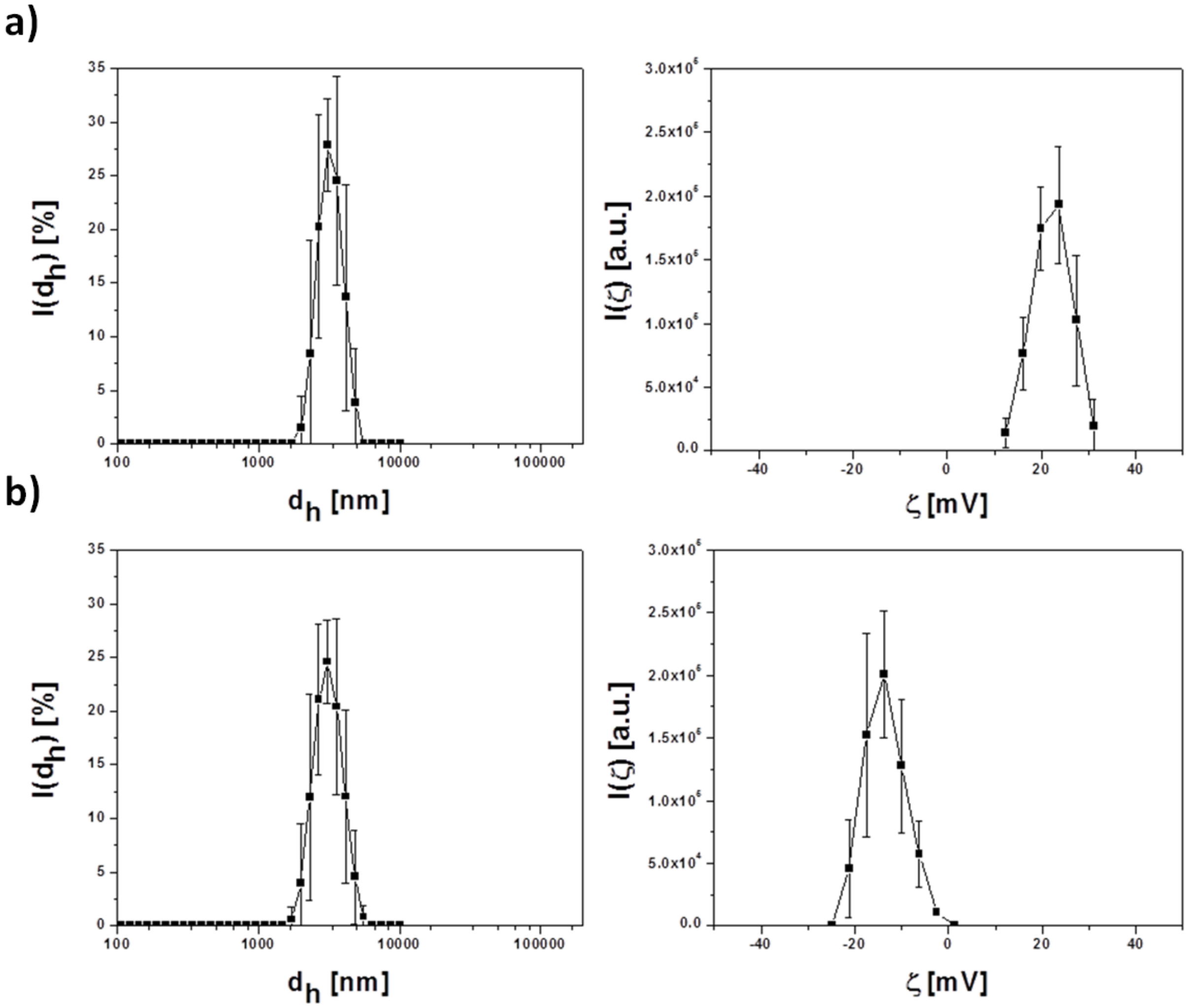

Figure SI-IV.2.1: The hydrodynamic diameters d_h in terms of the intensity distributions and zeta potentials ζ of GNP-loaded a) positively and b) negatively charged capsules as recorded in water. The results are presented as means of 3 measurements ± standard deviations. The corresponding values are provided in the Table SI-IV.2.1.

Capsule charge	d_h [µm]	Δd_h [µm]	ζ [mV]	$\Delta\zeta$ [mV]
+	3.1	0.004	22.33	2.165
-	3.1	0.004	- 13.7	2.165

Table SI-IV.2.1: Colloidal properties of the capsules. Hydrodynamic diameter $d_h \pm \Delta d_h$ and zeta potential $\zeta \pm \Delta\zeta$ as measured in water given as average and standard deviation. These data were derived from Figure SI-IV.2.1.

IV.3) Uptake studies by inductively coupled plasma mass spectroscopy (ICP-MS)

HeLa cells were seeded in 6 well plates (8.95 cm^2 surface area/well, 3 mL medium) at a density of 100,000 cells per well. After 24 h incubation fresh growth media, either serum supplemented or serum free, containing GNP-loaded capsules was provided to the cells. Both types of capsules (positively and negatively charged) were added in two different concentrations, at 10 or 20 capsules added per cell. HeLa cells were incubated with capsules for different times (2, 4, 6, 12, 24, 30, 36, 42, and 48 h) inside an incubator set at 37 °C with 5% CO_2 supply. Afterwards, the cells were washed with PBS and detached from the bottom of the plates by means of 0.05% trypsin-EDTA. The cells were centrifuged with growth media and the pellets were resuspended in PBS. The pellets of cells were washed again with PBS and the maximum of the supernatant was removed (note that remaining trypsin in solution would interfere with determining the protein concentration of the samples as described later). Then, 1x lysis buffer in water (100 µL per sample) was added to each cell pellet and the samples were incubated at room temperature for 30 minutes in order to complete cell lysis. Samples were sonicated and stored at -20 °C for further analysis, which involved determination of the amount of cells (by measuring the protein content) and the amount of capsules (by measuring the amount of elemental gold) in the cell lysates, as described in the following.

In order to determine the number of capsules per cell, the number of cells and the number of capsules in the cell lysate has to be determined. Cells were quantified by detecting their protein content by means of a commercial protein determination kit (Lowry assay; TPO300-KT). Lowry reagent solution was prepared by dissolving 2 g of Lowry reagent powder in 40 mL of water. The labeling solution was prepared by transferring 18 mL of Folin and Ciocalteu's phenol reagent into an amber glass, followed by the addition of 90 mL of water (in order to achieve the working concentration of labeling reagent). First, a calibration curve was plotted, relating the amount of the detected proteins to the number of cells. HeLa cells were detached from cell culture flasks by means of 0.05% trypsin-EDTA, followed twice by washing with PBS. The cells were then dispersed in a small volume of PBS and their number in terms of cells per volume of solution was determined by counting thrice with a haemocytometer. Then 1x lysis buffer was added. After cell lysis serial dilutions of the cell lysates in lysis buffer was performed in order to achieve samples with subsequently smaller cell concentrations. Lysis buffer was used for blank measurements. In sample tubes, 5 µL of cell lysates were added into 100 µL of

Lowry reagent solution, mixed well, followed by waiting for 20 minutes for completion of complex formation between proteins of the cell lysates and the Lowry reagent solution. Later, 50 µL of labeling reagent solution was added to the above mixture to develop blue color depending on protein content. After waiting for 30 minutes the absorption of samples was immediately measured by means of UV-visible absorption spectrometry. Spectra were recorded from 550 - 800 nm and from the spectra the absorption (A_{750}) at 750 nm was determined, cf. Figure SI-IV.3.1. The spectrum of lysis buffer without cells has been subtracted as blank. Then the absorption values A_{750} (corresponding to the protein contents in the samples) were plotted against the number of cells, leading to a calibration curve that correlates the absorption at 750 nm to the number of cells N_{cells}, cf. Figure SI-IV.3.1.

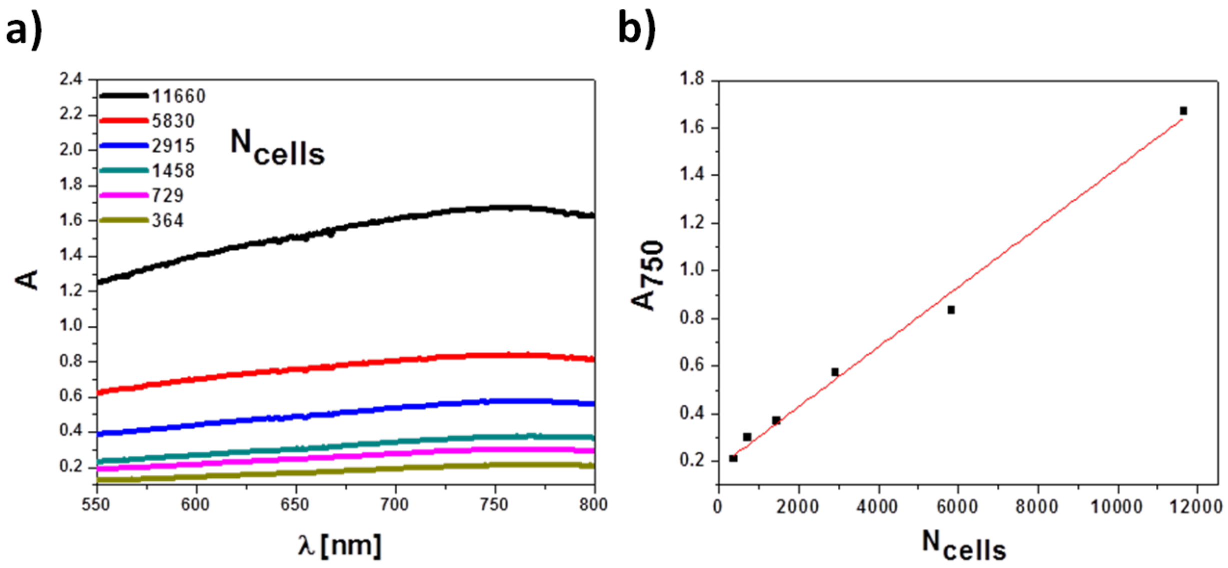

Figure SI-IV.3.1: a) Absorption spectra A of cell lysates (protein content) versus λ recorded at 550 - 800 nm derived from different number of cells (per µL of cell lysates). For each spectrum the absorption A_{750} at 750 nm was determined. b) Absorption values (A_{750}) of cell lysates at 750 nm plotted versus the number of cells N_{cells} per µL of samples. The slope of the fitted line was used for determining the number of cells in unknown samples from their absorption values at 750 nm.

The number of cells N_{cells} in the cell lysates obtained from cells with incorporated GNP-loaded capsules was then determined by the Lowry assays, and absorption of the lysates at 750 nm was converted into cell numbers using the calibration curve shown in Figure SI-IV.3.1b.

The amount of capsules in the cell lysates was determined in terms of elemental Au (from the GNPs) as measured with ICP-MS. The cell lysates containing the internalized GNP-loaded capsules were first digested with aqua regia. For this first the stock solution for each sample was prepared by adding 50 µL of sample suspension into freshly prepared 150 µL aqua regia (1 HNO_3 : 3 HCl) inside 6 mL perfluoroalkoxy alkane tubes (PFA), followed by mixing for at least 8 h under constant agitation. By doing this the GNP-loaded capsules and remaining organic

cell fragments were digested and broken down into small molecular / atomic components. In the second step, 4.6 mL HNO_3 solution (2%) as low matrix was introduced to each digested sample to prevent the aqua regia from digesting the ICP-MS machinery, as well as to provide an ion stable environment with constant background conditions for all samples. During measurements, 5 repetitions/ sample, and 100 sweeps were performed and a peak pattern of 3 peaks was used. The diluted samples were introduced to the ICP-MS set-up through an integrated autosampler coupled to a peltier cooling spray chamber where the samples were nebulized and taken up by the argon gas flow at a speed of ½ m/s. The concentration determination was performed using a calibration curve for Au consisting of 9 measurement points (0 - 2500 µg/L) of freshly prepared Au concentrations derived from gold standard solutions from Agilent (1000 mg/L). Results were obtained as the mean of five measurements in parts per billion or µg/L (ppb = µg/L). First, calibration was performed in which the Au content in the samples of GNP-loaded capsules of given number (as determined with a haemocytometer) was determined. From this the mass of elemental gold per capsule was determined. Then the amount of gold in the cell lysates of cells with incorporated GNP-loaded capsules was determined by ICP-MS. Using the calibration curve the number of capsules N_{caps} in the lysate was determined. Finally, the number of internalized capsules per cell was determined as $N_{caps/cell} = N_{caps}/N_{cells}$.

IV.4) Results

The data obtained for the determined number of capsules per cell $N_{caps/cell}$ is summarized in Figure SI-IV.4.1, and Table SI-IV.4.1 and Table SI-IV.4.2. From Figure SI-IV.4.1 it can be seen that the internalization of positively charged GNP-loaded capsules was higher than for their negatively charged counterparts. Uptake in serum deprived culture as compared to serum supplemented cell culture was also higher, which is in agreement with some previous findings [8, 17]. In addition, the uptake was dose dependent, i.e., more capsules were found internalized when cells were incubated with 20 capsules per cell as compared to the addition of 10 capsules per cell, which is also in agreement with previous work [16]. Note that for interpretation of the results it is important to realize that the number of capsules added per cell refers to the number of cells seeded. However, the number of internalized capsules per cell, refers to the number of cells which have been found with the Lowry assay after incubation with capsules. After approximately 24 h HeLa cells start to proliferate, which complicates the quantitative analysis [18]. Cell proliferation results in increase in cell number after capsule uptake as compared to the number of seeded cells.

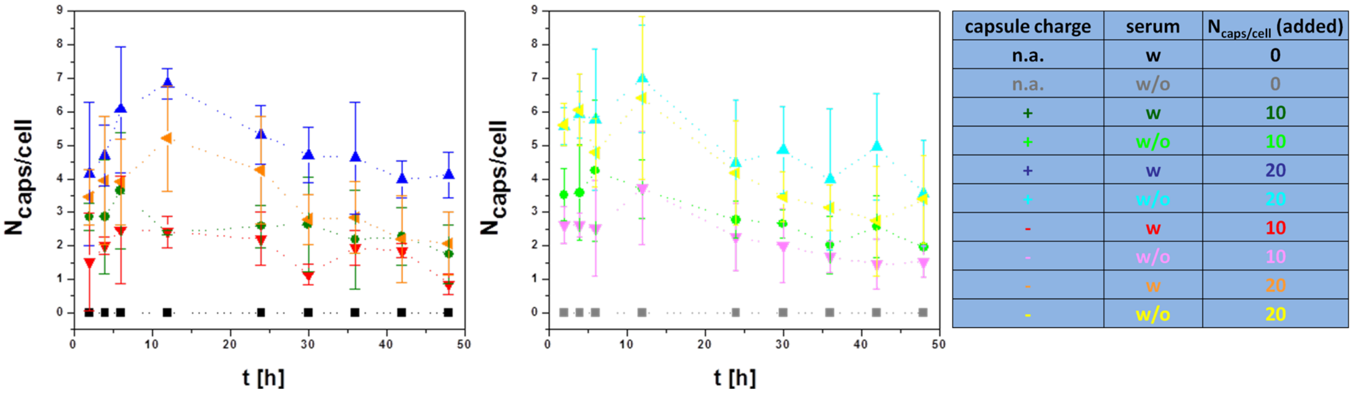

Figure SI-IV.4.1: Number of internalized capsules per cell $N_{caps/cell}$ after HeLa cells had been incubated for different times t in serum supplemented and serum deprived cell culture in the presence of positively and negatively charged GNP-loaded capsules which were provided at a concentration of $N_{caps/cell}$ (added). Data are presented as mean of 3 independent experiments ± standard deviations. The data are enlisted in Table SI-IV.4.1 and Table SI-IV.4.2. "n.a." = not applicable.

charge / t [h]	$N_{caps/cell}$ (added) = 0	$N_{caps/cell}$ (added) = 10		$N_{caps/cell}$ (added) = 20	
	n.a.	+	-	+	-
2	0.01 ± 0.006	2.87 ± 0.41	1.51 ± 1.46	4.16 ± 2.15	3.45 ± 0.82
4	0.004 ± 0.003	2.87 ± 1.7	2.01 ± 0.25	4.7 ± 0.91	3.96 ± 1.92
6	0.004 ± 0.002	3.64 ± 1.73	2.48 ± 1.6	6.1 ± 1.9	3.9 ± 1.27
12	0.005 ± 0.002	2.4 ± 0.08	2.43 ± 0.47	6.83 ± 0.45	5.21 ± 1.57
24	0.004 ± 0.002	2.6 ± 0.63	2.21 ± 0.79	5.31 ± 0.9	4.26 ± 1.61
30	0.004 ± 0.003	2.64 ± 1.4	1.15 ± 0.31	4.71 ± 0.83	2.8 ± 0.75
36	0.002 ± 0.000	2.2 ± 1.47	1.93 ± 0.52	4.62 ± 1.67	2.86 ± 1.06
42	0.003 ± 0.002	2.28 ± 0.86	1.85 ± 0.20	4 ± 0.55	2.2 ± 1.31
48	0.002 ± 0.000	1.75 ± 0.88	0.86 ± 0.32	4.12 ± 0.69	2.07 ± 0.93

Table SI-IV.4.1: Number of internalized capsules per cell $N_{caps/cell}$ after HeLa cells have been incubated for different times t in serum supplemented cell culture upon capsule incubation with $N_{caps/cell}$ (added). Data are presented as mean of 3 independent experiments ± standard deviations. The data row with charge = "n.a." (not applicable) corresponds to the control in which no capsules had been added to the cells.

charge / t [h]	$N_{caps/cell}$ (added) = 0	$N_{caps/cell}$ (added) = 10		$N_{caps/cell}$ (added) = 20	
	n.a.	+	-	+	-
2	0.006 ± 0.006	3.53 ± 0.79	2.62 ± 0.56	5.56 ± 0.54	5.62 ± 0.64
4	0.005 ± 0.003	3.6 ± 1.43	2.62 ± 0.35	5.91 ± 0.68	6.05 ± 1.07
6	0.006 ± 0.002	4.25 ± 2.12	2.52 ± 1.42	5.77 ± 2.12	4.78 ± 1.01
12	0.009 ± 0.005	3.7 ± 0.89	3.73 ± 1.69	6.99 ± 1.61	6.42 ± 2.43
24	0.008 ± 0.005	2.78 ± 0.56	2.26 ± 1.01	4.48 ± 1.87	4.18 ± 1.55
30	0.005 ± 0.000	2.66 ± 0.42	2.01 ± 1.1	4.85 ± 1.29	3.46 ± 0.74
36	0.005 ± 0.001	2.02 ± 0.87	1.67 ± 0.47	3.98 ± 2.11	3.15 ± 0.68
42	0.004 ± 0.000	2.57 ± 0.92	1.45 ± 0.74	4.95 ± 1.59	2.74 ± 1.63
48	0.005 ± 0.002	1.96 ± 0.09	1.53 ± 0.48	3.57 ± 1.58	3.39 ± 1.32

Table SI-IV.4.2: Number of internalized capsules per cell $N_{caps/cell}$ after HeLa cells had been incubated for different times [t] in serum free cell culture upon capsule incubation with $N_{caps/cell}$ (added). Data are presented as mean of 3 independent experiments ± standard deviations.

In addition, from Figure SI-IV.4.1 the maximum amount of capsules internalized per cell under saturation conditions $N_{caps/cell\ (sat,i)}$, and the time $t_{up(sat,i)}$ it took until cells had incorporated 50% of the maximum number of capsules per cell are reported in Table SI-IV.4.3 and Table SI-IV.4.4, respectively.

charge	+	+	+	+	-	-	-	-
serum	w	w/o	w	w/o	w	w/o	w	w/o
$N_{caps/cell}$ (added)	10	10	20	20	10	10	20	20
$N_{caps/cell\ (sat,i)}$	3.64	4.25	6.83	6.99	2.48	3.73	5.21	6.42

Table SI-IV.4.3: Number of capsules internalized per cell under saturation conditions $N_{caps/cell\ (sat,i)}$, as derived from Figure SI-IV.4.1.

charge	+	+	+	+	-	-	-	-
serum	w	w/o	w	w/o	w	w/o	w	w/o
$N_{caps/cell}$ (added)	10	10	20	20	10	10	20	20
$t_{up(sat,i)}$ [h]	< 2	< 2	< 2	< 2	< 2	< 2	< 2	< 2

Table SI-IV.4.4: Time $t_{up(sat,i)}$ until 50% of the saturation intensity upon capsule internalization has been reached as derived from Figure SI-III.4.1.

V) Viability measurements of cells exposed to capsules and inhibitor

V.1) Materials and reagents
V.2) Viability measurements
V.3) Results

V.1) Materials and reagents

96 well assay plates (clear bottom, # 3603) were purchased from Corning. Resazurin solution (alamar blue; #TOX-8) was purchased from Sigma-Aldrich. All other reagents, chemicals, and consumables were the same as described in sections §I.1 and §II.1.

Fluorescence measurements were performed with a Fluorolog®-3 spectrofluorometer from HORIBA JOBIN YVON. For plate reading a Micromax 384 microwell-plate reader compatible with the Fluorolog® was used.

V.2) Viability measurements

Resazurin based cytotoxicity assays were performed to determine the impact of GNP-loaded PEM capsules and cytochalasin D (which was used in the studies presented in section §I) exposure on cell viability. The test is based on mitochondrial activity of living cells [19]. Active mitochondria of living cells cause bioreduction of the dye, i.e. they convert the non-fluorescent blue dye (resazurin) into its reduced form resorufin which fluoresces in red. In order to perform the studies, HeLa cells were seeded in 96 well transparent bottom plates (7500 cells/well, 0.32 cm^2 area/well) in 100 µL of complete growth media (DMEM supplemented with 10% FBS, 1% glutaMAX^{TM} and 1% P/S) and were incubated for 24 h at 37 °C with a constant supply of CO_2 (5%). After 24 h, the old growth media was replaced by fresh growth media containing capsules or cytochalasin D at different concentrations, i.e. $N_{caps/cell}$ (added), and c(Cytochalasin D). Capsules were added at $N_{caps/cell}$ (added) 10 or 20 capsules per cell for each condition (positive and negative capsules in serum supplemented and serum deprived cell culture media). In case of cytochalasin D serial dilution was performed to examine its toxic effect for a broad range of concentrations (2400 - 4.5 × 10^{-06} µM). Experiments for each dose (for capsules and cytochalasin D) were performed in triplicate. As negative control in a few wells of the assay plates, fresh growth media was added to the cells without capsules or cytochalasin D. In case of capsules fresh serum supplemented and serum deprived media was provided to the cells as negative control for each sample/condition. Whereas, for cytochalasin D as negative control fresh serum supplemented cell growth media was provided to the cells in the absence of cytochalalsin D. Cells were incubated with capsules for different time points, i.e., 2, 4, 6, 12, 24, 30, 36, 42, 48 h, while 24 h the incubation time with cytochalasin D was used. After incubation for defined time points, the cells were washed

with PBS and 10% resazurin solution in growth media was added (100 µL/well) to the cells. In some wells of the assay plate only resazurin solution (10%) was added (without cells) which served as blank and the assay plates were incubated for 4 h under the same conditions as described above. Then, the fluorescence spectra of each well of the assay plates were recorded by means of a microplate reader attached to a spectrofluorometer. Spectra were recorded using an excitation wavelength of 560 nm acquiring the emission spectra from 572 to 650 nm [20]. Background emission of the blank sample containing only resazurin solution was subtracted from all spectra. For getting the viability V of cells the mean of fluorescence value of each sample was normalized with respect to fluorescence of the control samples in which cells were not exposed to capsules or cytochalasin D [20]. Experiments were performed in triplicate and values are expressed as means of 3 independent experiments ± standard deviations.

V.3) Results

The viability (V) of HeLa cells upon exposure to cytochalasin D and PEM capsules with incorporated GNPs is presented in Figure SI-V.3.1 and Figure SI-V.3.2, respectively.

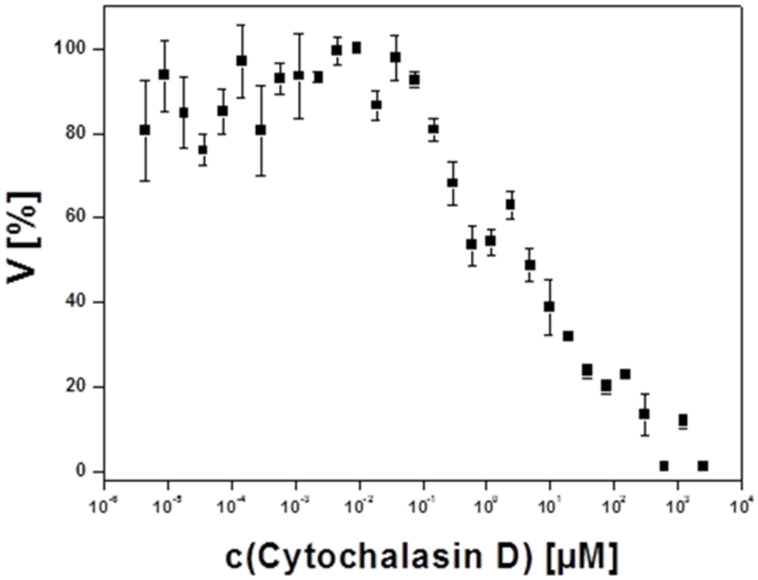

Figure SI-V.3.1: Cell viability V[%] of HeLa cells upon exposure (24 h) to different doses of cytochalasin D under serum supplemented condition. Data is presented as mean of three measurements with corresponding standard deviations. The dose used in experiments presented in section §I (0.3 µM) was thus at a level where some reduction of cell viability already occurred.

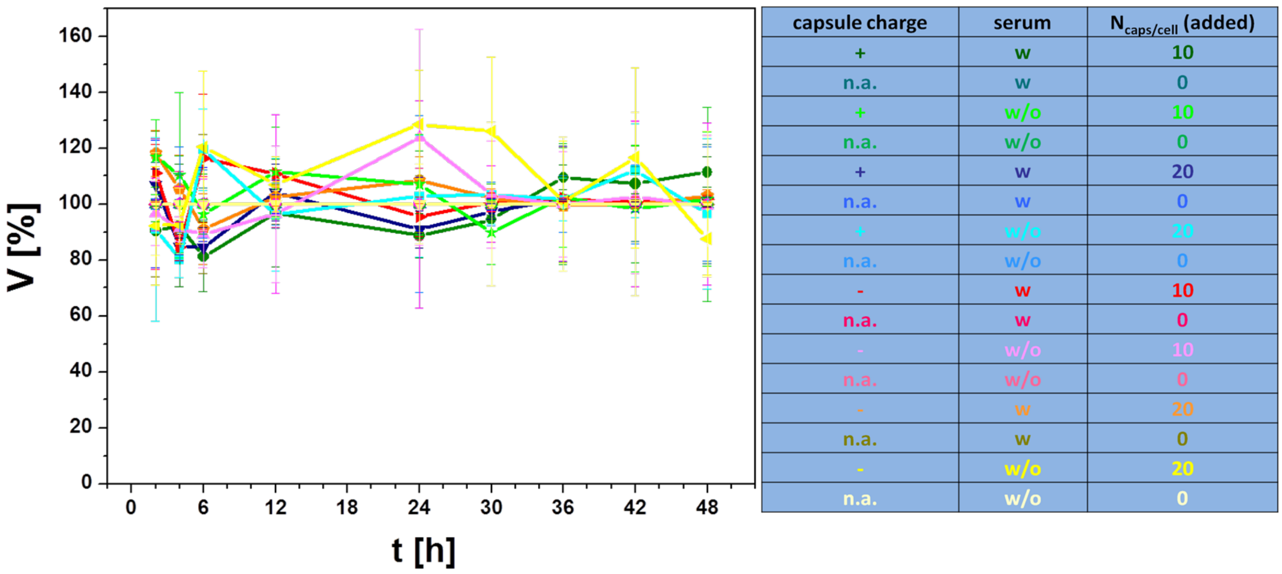

capsule charge	serum	$N_{caps/cell}$ (added)
+	w	10
n.a.	w	0
+	w/o	10
n.a.	w/o	0
+	w	20
n.a.	w	0
+	w/o	20
n.a.	w/o	0
-	w	10
n.a.	w	0
-	w/o	10
n.a.	w/o	0
-	w	20
n.a.	w	0
-	w/o	20
n.a.	w/o	0

Figure SI-V.3.2: Cell viability V[%] of HeLa cells upon exposure to different doses of capsules under serum supplemented and serum deprived conditions. Data is presented as mean of three measurements with corresponding standard deviations. The doses of capsules used in the experiments are non-toxic under the given experimental conditions.

VI) References

[1] aA. P. Petrov, D. V. Volodkin, G. B. Sukhorukov, *Biotechnol. Progr.* **2005**, *21*, 918-925; bP. Rivera_Gil, S. D. Koker, B. G. D. Geest, W. J. Parak, *Nano Lett.* **2009**, *9*, 4398-4402; cL. J. De Cock, S. De Koker, B. G. De Geest, J. Grooten, C. Vervaet, J. P. Remon, G. B. Sukhorukov, M. N. Antipina, *Angew. Chem. Int. Ed.* **2010**, *49*, 6954-6973; dL. L. del Mercato, A. Z. Abbasi, W. J. Parak, *Small* **2011**, *7*, 351-363; eS. De Koker, R. Hoogenboom, B. G. De Geest, *Chem. Soc. Rev.* **2012**, *41*, 2867-2884; fR. Hartmann, M. Weidenbach, M. Neubauer, A. Fery, W. J. Parak, *Angew. Chem. Int. Ed.* **2015**, *54*, 1365-1368.

[2] aS. De Koker, B. G. De Geest, C. Cuvelier, L. Ferdinande, W. Deckers, W. E. Hennink, S. De Smedt, N. Mertens, *Adv. Funct. Mater.* **2007**, *17*, 3754-3763; bS. Carregal-Romero, M. Ochs, P. Rivera Gil, C. Ganas, A. M. Pavlov, G. B. Sukhorukov, W. J. Parak, *J. Control. Release* **2012**, *159*, 120-127.

[3] J. Y. Han, K. Burgess, *Chem. Rev.* **2010**, *110*, 2709-2728.

[4] A. Masuda, M. Oyamada, T. Nagaoka, N. Tateishi, T. Takamatsu, *Brain Res.* **1998**, *807*, 70-77.

[5] P. Rivera Gil, M. Nazarenus, S. Ashraf, W. J. Parak, *Small* **2012**, *8*, 943-948.

[6] R. C. Gonzalez, R. E. Woods, *Digital Image Processing*, Pearson Education, Upper Saddle River, NJ, **2008**.

[7] J. C. Crocker, D. G. Grier, *J. Colloid Interface Sci.* **1996**, *179*, 298-310.

[8] L. Kastl, D. Sasse, V. Wulf, R. Hartmann, J. Mircheski, C. Ranke, S. Carregal-Romero, J. A. Martínez-López, R. Fernández-Chacón, W. J. Parak, H.-P. Elsaesser, P. Rivera Gil, *ACS Nano* **2013**, *7*, 6605-6618.

[9] C. Sevcik, *Complexity International* **2010**, *5*, 1-19.

[10] aM. J. Katz, *Comput. Biol. Med.* **1988**, *18*, 145-156; bR. Esteller, G. Vachtsevanos, J. Echauz, B. Litt, *IEEE Transactions on Circuits and Systems I: Fundamental Theory and Applications* **200**, *48*, 177-183.

[11] P. G. de_Gennes, *Science* **1997**, *276*, 1999.

[12] O. Kreft, A. Muñoz Javier, G. B. Sukhorukov, W. J. Parak, *J. Mater. Chem.* **2007**, *17*, 4471-4476.

[13] B. Parakhonskiy, M. V. Zyuzin, A. Yashchenok, S. Carregal-Romero, J. Rejman, H. Mohwald, W. J. Parak, A. G. Skirtach, *J Nanobiotechnology* **2015**, *13*, 53.

[14] aG. B. Sukhorukov, A. L. Rogach, B. Zebli, T. Liedl, A. G. Skirtach, K. Köhler, A. A. Antipov, N. Gaponik, A. S. Susha, M. Winterhalter, W. J. Parak, *Small* **2005**, *1*, 194-200; bB. Zebli, A. S. Susha, G. B. Sukhorukov, A. L. Rogach, W. J. Parak, *Langmuir* **2005**, *21*, 4262-4265; cA. Muñoz Javier, O. Kreft, A. Piera Alberola, C. Kirchner, B. Zebli, A. S. Susha, E. Horn, S. Kempter, A. G. Skirtach, A. L. Rogach, J. Rädler, G. B. Sukhorukov, M. Benoit, W. J. Parak, *Small* **2006**, *2*, 394-400.

[15] M. Semmling, O. Kreft, A. Muñoz Javier, G. B. Sukhorukov, J. Käs, W. J. Parak, *Small* **2008**, *4*, 1763-1768.

[16] M. V. Zyuzin, P. Díez, M. Goldsmith, S. Carregal-Romero, C. Teodosio, J. Rejman, N. Feliu, A. Escudero, M. J. s. Almendral, U. Linne, D. Peer, M. Fuentes, W. J. Parak, *Bioconjugate Chem.* **2017**, *28*, 556−564.

[17] L. Treuel, M. Malissek, S. Grass, J. Diendorf, D. Mahl, W. Meyer-Zaika, M. Epple, *J. Nanopart. Res.* **2012**, *14*.

[18] aJ. A. Kim, C. Aberg, A. Salvati, K. A. Dawson, *Nature Nanotechnology* **2012**, *7*, 62-68; bC. Aberg, J. A. Kim, A. Salvati, K. A. Dawson, *Epl* **2013**, *101*.

[19] aJ. O'Brien, I. Wilson, T. Ortaon, F. Pognan, *Toxicology* **2001**, *164*, 132-132; bJ. O'Brien, I. Wilson, T. Orton, F. o. Pognan, *Eur. J. Biochem.* **2000**, *267*, 5421-5426.

[20] X. Ma, R. Hartmann, D. J. d. Aberasturi, F. Yang, S. J. H. Soenen, B. B. Manshian, J. Franz, D. Valdeperez, B. Pelaz, N. Feliu, N. Hampp, C. Riethmüller, H. Vieker, N. Frese, A. Gölzhäuser, M. Simonich, R. L. Tanguay, X.-J. Liang, W. J. Parak, *ACS Nano* **2017**, *11*, 7807−7820.
